# An Orally Administered Misuse Deterrent Opioid Prodrug for Treatment of Acute Pain

**DOI:** 10.1021/jacsau.5c01126

**Published:** 2025-10-15

**Authors:** Douglas A. Rose, Joseph W. Treacy, Karina Seth, Anthony F. Tanzillo, Allison Li, Kyle Tamshen, Lily K. Sloan, Natalie Boehnke, Christopher J. Evans, Catherine M. Cahill, Heather D. Maynard

**Affiliations:** † Department of Chemistry and Biochemistry, University of California, Los Angeles, 607 Charles E. Young Drive East, Los Angeles, California 90095-1569, United States; ‡ Department of Psychiatry and Behavioral Science, Shirley and Stefan Hatos Center for Neuropharmacology, Semel Institute for Neuroscience and Human Behavior, University of California, Los Angeles, 760 Westwood Plaza, Los Angeles, California 90095-8353, United States; § Department of Chemical Engineering and Materials Science, 5635 University of Minnesota Twin Cities, Minneapolis, Minnesota 55455, United States; ∥ California NanoSystems Institute, University of California, Los Angeles, 570 Westwood Plaza, Los Angeles, California 90095, United States

**Keywords:** Peptide prodrug, oxycodone, misuse deterrence, self-immolative

## Abstract

Opioids are a powerful class of medicines due to their ability to alleviate acute pain. However, the use of prescription opioids has led to an epidemic in the United States. Many efforts to combat this are ongoing, including the preparation of misuse deterrent opioid formulations, some of which can be subverted using common household chemicals. Herein, the development of an oxycodone-containing peptide prodrug is reported that contains three covalent misuse deterrent protective layers. This prodrug is resistant to degradation in the presence of acidic and basic pH conditions, common household chemicals, and enzyme supplements. After optimization of the peptide sequence, the lead prodrug is composed of a branched lysine residue coupled to a *tert*-butyl-protected tyrosine that is not naturally recognized by digestive enzymes. There is a necessary sequence of protecting group removal and then subsequent enzymatic peptide cleavages that trigger cyclization of a self-immolative linker to release the opioid agonist, oxycodone. The prodrug has analgesic properties *in vivo* in mice only after oral administration and not by intraperitoneal injection, which suggests that this prodrug may have misuse deterrent properties. The specificity for dual-enzyme release and the resulting *in vivo* studies warrant further examination of this prodrug scaffold for its potential as a misuse deterrent alternative to treat acute pain.

## Graphical abstract

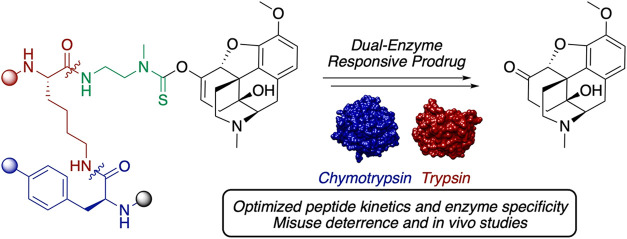

## Introduction

The United States has a tumultuous past with opioids dating back to the late 19th and early 20th centuries after the first chemical isolation of heroin from opium in 1898.^1^ The consumption and production of semisynthetic and synthetic opioids have evolved over time, but they still remain highly addictive substances. In 2021, the CDC reported more than 16,500 prescription opioid-associated overdose deaths in the United States, and in 2023, 8.9 million people were reported to have misused opioids—8.2 million of whom misused prescription opioids.^2,3^ The increase in the prescription opioid use in the United States over the past few decades has been traced back to multiple factors,^4–6^ and the use of prescription opioids can initiate the transition of users to injection-based opioids such as heroin and fentanyl.^7–12^ In 2017, the US Department of Health and Human Services formally recognized the opioid epidemic as a national health crisis. Consequently, a plan of action was implemented to reverse the damage done by this class of pharmaceuticals.^13^ As a result, there has been an increase in efforts to develop new drugs or drug delivery systems to combat this crisis. Specifically, one area of research is the study of nonopioid-based treatments that target proteins involved in nociception or inflammation signaling pathways.^14,15^ Recently, the FDA approved the highly selective Na_V_1.8 pain signal inhibitor JOURNAVX (suzetrigine) for the treatment of acute pain.^16^ Another alternative is the development of opioid formulations that contain misuse deterrent engineering controls, which are designed to mitigate the typical forms of misuse by increasing the difficulty of extracting large amounts of active opioid for instant release in pursuit of the euphoric effects.^17–20^

The FDA has described five general strategies for developing misuse deterrent formulations including physical or chemical barriers to mechanical alteration, agonist/antagonist combinations, coformulation with aversive substances released upon tampering, unconventional delivery systems (e.g., subcutaneous implants), and prodrugs that are only activated following oral administration.^21^ There are currently four formulations with FDA-approved labeling describing misuse deterrent properties, three with oxycodone and one with hydrocodone as the opioid.^17,22^ All of these formulations rely on a noncovalent encapsulation strategy, wherein the opioid is sequestered within a porous polymeric network and slowly released to prevent the rapid onset of euphoria while still maintaining the analgesic properties of the opioid. However, many of these engineering controls can be circumvented by knowledgeable users employing household supplies, thereby reducing their effectiveness.^23,24^

A promising alternative to these noncovalent misuse deterrent strategies is to employ covalent modifications in the form of a prodrug. Prodrug strategies have been widely used in cancer drug delivery and antimicrobials,^25–30^ whereas fewer examples have been developed in opioid delivery and prevention of opioid overdose.^31–34^ However, these prodrugs could provide targeted and controlled release of the opioid to prevent a burst release that may lead to an adverse health event. Designing the prodrug to target opioid release within the gastrointestinal tract may safeguard against intravenous and intranasal routes of misuse. This covalent prodrug strategy is currently being pursued by two separate companies, all of which are in phase three clinical trials.^35^ Two of these prodrugs take advantage of the acidic conditions in the stomach to cleave either an ester or phosphoester modification of the opioid. However, these strategies can be circumvented through chemical manipulations using household supplies, allowing users to bypass the engineering controls. The third prodrug relies on trypsin, a protease found in the small intestine, to enzymatically cleave the prodrug and release oxycodone.^36,37^ This prodrug has an enhanced misuse deterrence profile,^36^ but it still relies on one stimulus to release the active opioid.

To address these shortcomings and add further layers of protection,^38^ we have designed a multiple stimuli-responsive, misuse deterrent opioid prodrug (Figure 1A). This strategy relies upon a covalent linkage of oxycodone to an enzymatically labile peptide, whereupon sequential incubation with acid and the digestive enzymes trypsin and chymotrypsin, the native opioid is released. Critically, this strategy is only amenable to oral delivery of the prodrug, and it should not provide analgesic effects when delivered intravenously, thus discouraging users from misusing the opioid. We envisioned that the acidic conditions in the stomach would remove the *tert*-butyl ether group from tyrosine, allowing chymotrypsin to recognize and cleave the isopeptide bond between the tyrosine residue and the ε-amine of a lysine residue.^39^ Once cleaved, the lysine residue can further be digested by trypsin, which cleaves the lysine *C*-terminally and releases a primary amine.^40,41^ This primary amine can then rapidly cyclize^42,43^ and release the active opioid, forming 1-methylimidazolidine-2-thione as a nontoxic byproduct.^44^

**Figure 1:**
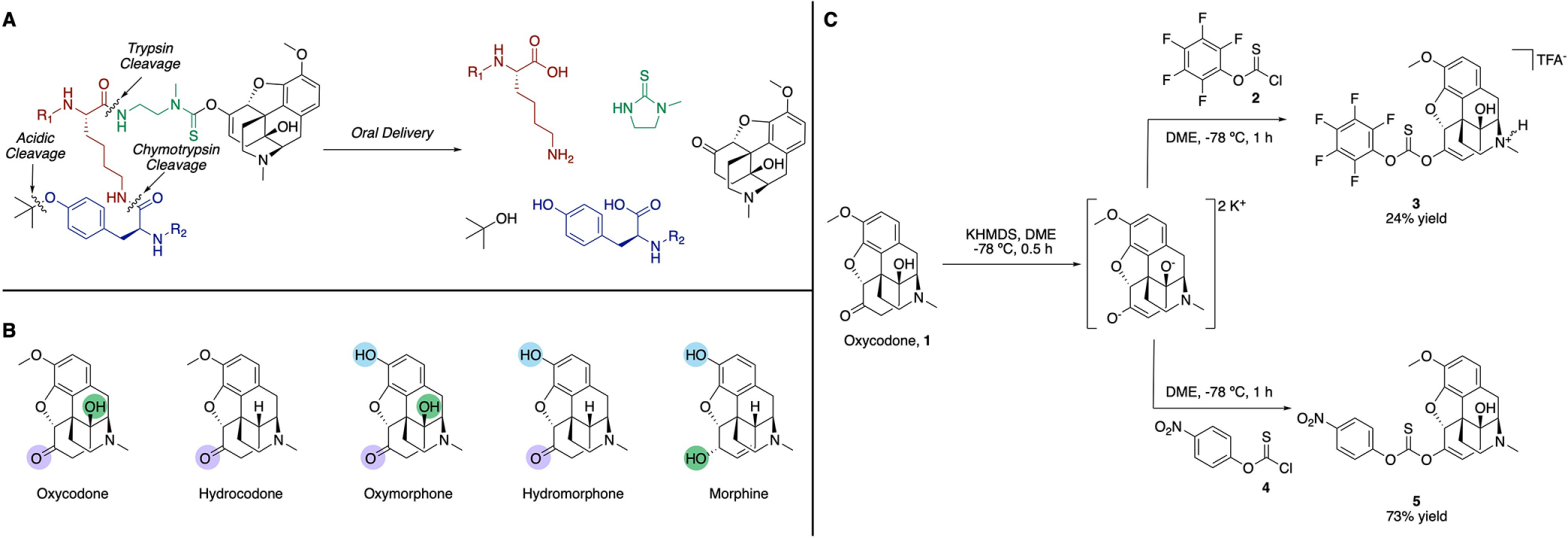
(A) Proposed scheme of misuse deterrent opioid prodrug design relying on three protective layers. (B) Evaluation of functional groups amenable to reversible derivatization among five of the most commonly prescribed opioids. (C) Synthesis of oxycodone electrophiles **3** and **5** for coupling to the peptide prodrug scaffold.

## Results and Discussion

### Evaluation of Common Opioids for Reversible Modification

Examining the structures of five of the most commonly prescribed opioids reveals multiple points for derivatization and subsequent attachment to the peptide scaffold.^45^ Conserved across four of these opioids is the ketone motif, which we identified as a potential reversible linkage point for the peptide (Figure 1B). By forming the enolate, we could generate a nucleophilic moiety and couple it to a thionochloroformate or chloroformate electrophile. This electrophile could then react with an amine to form a stable thionocarbamate or carbamate linkage and the covalent prodrug. With oxycodone being the most commonly used opioid in the misuse deterrent formulations with FDA-approved labeling, we attempted a selective modification of the ketone of oxycodone (**1**). An initial investigation showed that the tertiary alcohol of oxycodone was unreactive toward modifications, enhancing our optimism that the enolate strategy would be ideally suited for selective modification of oxycodone. We also observed that the thionocarbonate product was less susceptible to hydrolysis compared to the carbonate (data not shown), likely due to its lower electrophilicity, so the thionocarbonate was chosen for the synthesis of all oxycodone-containing electrophiles going forward.

To ensure selective modification of the *O*-enolate, we screened conditions from previous reports, which indicate that the use of a coordinating solvent and a hard electrophile favors modification of the *O*-enolate over the *C*-enolate.^46^ We found that the use of potassium bis­(trimethylsilyl)­amide (KHMDS) as a base, dimethoxyethane (DME) as a solvent, and pentafluorophenyl *O*-thionochloroformate (**2**) as an electrophile minimized *C*-modification products and progressed to full conversion from the ketone. However, the thionocarbonate product **3** was isolated in only 24% yield (Figure 1C). The low yield following purification was likely due to the instability of **3** toward chromatography. Further optimization of the electrophile synthesis resulted in the use of *p*-nitrophenol *O*-thionochloroformate (**4**) that was amenable to purification via precipitation, which afforded activated oxycodone-containing thionocarbonate **5** in 73% yield (Figure 1C).

### Optimization of the Peptide Sequence for Release

Using the branched lysine peptide sequences modified from our previous work,^47^ we began to explore the sequence effects on the efficiency and release rate of a model reporter molecule, *p*-nitrophenol (*p*NP). Peptides **6**–**8** were initially prepared to determine whether the peptide backbone length had any effect on the release of *p*NP in the presence of chymotrypsin alone (Figure 2A; see Supporting Information (SI) for synthetic procedures). Chymotrypsin promiscuity (*i.e*., cleavage of both sites) could lead to less misuse deterrence as only one enzyme could promote the release of the opioid. Substrates **6** and **7** both showed less than 15% release over the course of 4 h (Figures 2A, S13, and S14). However, substrate **8** reached greater than 90% *p*NP release within 3 h (Figures 2A and S15). This illustrates that increasing the length of the peptide backbone by at least two amino acids directly facilitates chymotrypsin promiscuity, leading to the release of the *p*NP reporter. Whether the addition of these two alanine residues affected the conformation of the peptide sequence in the chymotrypsin binding pocket was not investigated; however, identifying a prodrug scaffold with minimal release in the presence of only chymotrypsin was successful (*i.e*., **6** and **7**), so we proceeded toward further optimization of the prodrug sequence with this new structure–activity insight.

**Figure 2:**
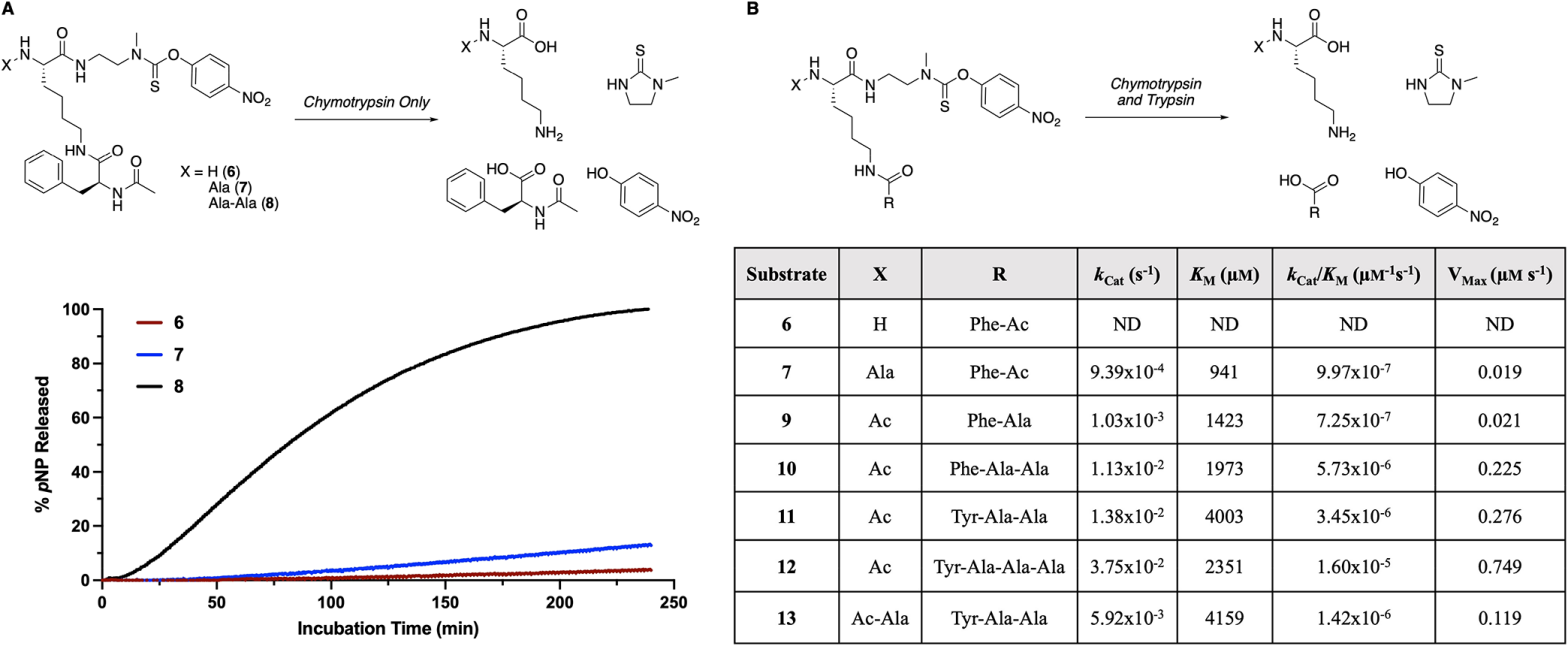
(A) Effect of the α-amine chain length on *p*NP release in the presence of chymotrypsin only. (B) Michaelis–Menten kinetics for various substrates in the presence of both chymotrypsin and trypsin. ND = not detectable. See SI for details on individual release assays.

In order to ensure the prodrug would be cleaved effectively once it was exposed to chymotrypsin and trypsin in concentrations found in the stomach,^48^ we elected to optimize the peptide sequence further, particularly on the ε-amine of the lysine, to enhance the substrate efficiency (Figure 2B). This would help to ensure that oxycodone is released in the gastrointestinal tract. To examine substrate specificity, Michaelis–Menten kinetics for substrates **6** and **7** were determined in the presence of both chymotrypsin and trypsin, but there was negligible release over 4 h for **6**, while **7** had a substrate specificity (*k*_Cat_/*K*_M_) of 9.97 × 10^–7^ μm ^–1^ s^–1^ (Figure 2B). Previous studies have shown that chymotrypsin binding can be enhanced through additional nonbulky amino acid groups following the aromatic residue.^39,49–51^ Accordingly, substrates **9** and **10**, with alanine residues coupled to phenylalanine, were prepared, and their kinetics were examined. Both **7** and **9** showed comparable substrate specificity, but the additional alanine on **10** resulted in significantly enhanced specificity (5.73 × 10^–6^ μm ^–1^ s^–1^). Substitution of phenylalanine with tyrosine has been shown to increase substrate specificity with chymotrypsin, so we synthesized the tyrosine-containing substrate **11** and observed a slight decrease in substrate specificity relative to the phenylalanine analog **10**. Coupling a third alanine to the tyrosine residue (**12**) increased the substrate specificity by around 5-fold compared to **11**. Since coupling of alanine residues to the α-amine of **6**–**8** increased the percentage of *p*NP release (Figure 2A), **13** with a single alanine on the *N*-terminus was prepared. Compared to substrate **11** with no *N*-terminal alanine, there was a minimal effect on the *K*_M_, but the *V*_Max_ was reduced by half, leading to a lower *k*_Cat_/*K*_M_. Since **12** had the highest *k*_Cat_/*K*_M_ and the fastest *V*_Max_, we proceeded to use this peptide scaffold for further experiments.

### Addition of a Third Protective Layer for Misuse Deterrence

While the addition of another alanine residue on the ε-peptide chain of **10** may result in a substrate specificity comparable to that of **12**, the use of tyrosine incorporated a phenol into the peptide, which could be functionalized to add an additional layer of misuse deterrence within the prodrug. We hypothesized that by protecting the phenol with a *tert*-butyl ether, chymotrypsin would not recognize the tyrosine derivative prior to passage through the acidic conditions in the stomach where the *tert*-butyl ether would be removed.^52^ This additional security measure removes the ability to pretreat the prodrug with store-bought digestive enzyme supplements to release the opioid. To probe this hypothesis, *in vitro* simulated digestion assays were carried out with **12** (−OH) and **14** (−O^*t*^Bu) in the presence of trypsin, chymotrypsin, or both enzymes (Figure 3A). *p*NP release from **12** was only observed in the presence of both proteolytic enzymes (Figure 3B), validating the necessity of both chymotrypsin and trypsin for the release of *p*NP. However, the presence of a *tert*-butyl ether in **14** protecting the phenol greatly reduced the amount of *p*NP released (∼5% release over 150 min), confirming that an additional acidic pretreatment is required for this prodrug scaffold to release the model reporter (Figure 3C).

**Figure 3:**
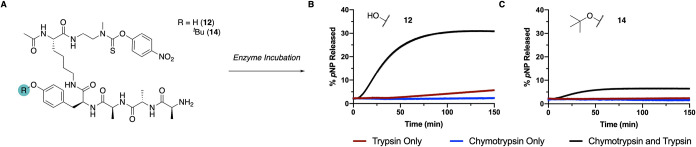
(A) Enzymatic release assay of *p*NP-containing peptides **12** and **14**. (B) *p*NP release curves from the **12**. (C) *p*NP release curves from **14**.

### Misuse Deterrence Testing of Prodrugs against Common Chemicals

To determine if this peptide scaffold was stable to different pH solutions and household chemicals that can break down other misuse deterrent formulations, the oxycodone-containing prodrug was synthesized (Figure 4A; see SI for details). Peptide **15** was synthesized by Fmoc solid-phase peptide synthesis using a 2-chlorotrityl chloride resin. Notably, the *N*-Fmoc group from the final coupling was left intact, and the O^*t*^Bu group was also retained through the selective cleavage of the peptide from the resin using hexafluoroisopropanol. Coupling of **16** and subsequent chemoselective deprotection of the Cbz group afforded **17**. The amine then underwent selective formation of the thionocarbamate using the oxycodone-containing electrophile **5**, and then deprotection of the Fmoc group generated the oxycodone-containing prodrug **18** with the O^*t*^Bu group intact in four linear steps from peptide **15** (Figure 4A). To examine the stability of this construct, **18** was incubated for 24 h at 23 °C with a variety of household chemicals and showed no detectable release of oxycodone (Figure 4B; see SI for details). Additionally, a store-bought digestive enzyme kit was unable to cleave the prodrug and to provide any release of oxycodone (Figure 4B). Prodrug **18** was also stable in different buffers varying from pH 2.0 to 10.0 with no observable release of oxycodone over 24 h at 23 °C (Figure 4B). The misuse deterrence profile of this prodrug indicates that it is challenging to extract oxycodone from this prodrug scaffold using commonly available chemicals.

**Figure 4:**
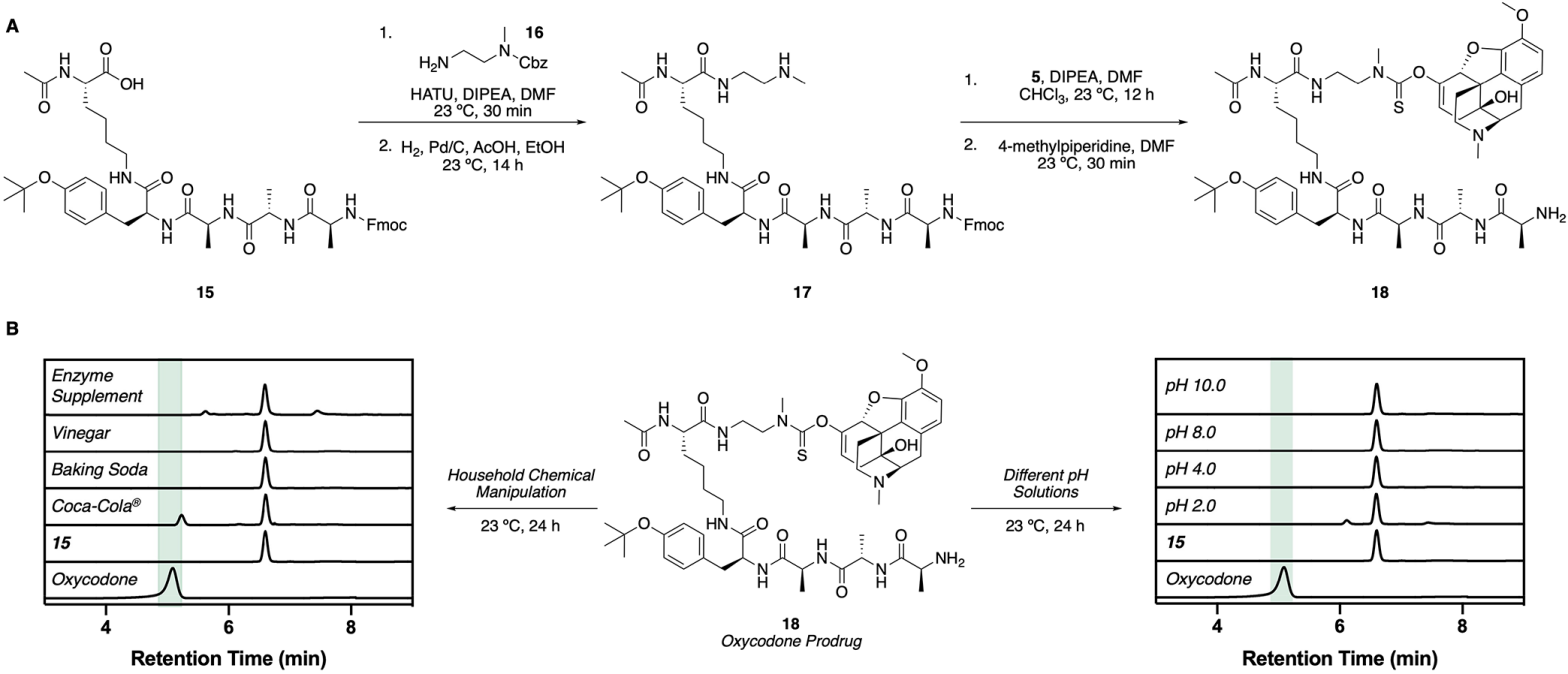
(A) Synthetic scheme of prodrug **18** from peptide **15**. Salts of the isolated amines are not shown for the sake of clarity. See SI for further details on the synthesis of **18**. (B) Household chemical and pH-based manipulation of oxycodone-containing prodrug **18**. HPLC traces are shown at 254 nm. The peak at 5.3 min in the Coca-Cola run corresponds to aspartame.

### Peptide Byproduct Inhibition Studies

Prior to moving forward with analgesia studies *in vivo*, it was important to ensure that the AAAY (**19**) peptide byproduct from chymotrypsin cleavage was not competitively inhibiting the *p*NP release. This could be an issue *in vivo*, as the PEPT1 transepithelial transporter in the digestive tract has a very weak binding affinity for tetrapeptides.^53^ A lack of transport for **19** could lead to the accumulation and generation of a competitive inhibitor for chymotrypsin, thus reducing the overall amount of opioid released. To examine this hypothesis, Michaelis–Menten competitive inhibition enzyme kinetics were performed with chymotrypsin and trypsin for **12** in the presence of peptide **19**. The activity of **12** proved to be negligibly affected by the presence of **19** (see SI, Figure S30). These observations enhanced our confidence in using the **12** prodrug scaffold for *in vivo* analgesia studies.

### *In Vivo* Determination of Analgesic Effects

The use of **18** for *in vivo* studies was precluded due to the decreased acidity of the mouse stomach compared to the human stomach (*ca.* pH 4.0 compared to 1.5 of the empty human stomach).^54^ This less acidic environment does not cleave the *tert*-butyl ether that is necessary for the enzymatic recognition of tyrosine, and thus prevents the release of oxycodone. Accordingly, the synthesis of prodrug scaffold **18** was modified to remove the *tert*-butyl group and afford the oxycodone prodrug **20** (Figure 5A; see SI for details). Hot plate latency, a measure of acute pain response, was used to determine the antinociceptive effects of the oxycodone prodrug.^55^ Oxycodone was administered as a positive control at a dose of 3 mg/kg (PO gavage). Since the *in vitro* simulated digestion assays stagnated at ∼30% release of *p*NP from **12** (Figure 3B), a higher dose of 30 mg/kg of oxycodone in **20** was used to determine the analgesic effects of the prodrug. Results for **20** indicate that the thermal threshold latency was increased to a level similar to that of the oxycodone positive control. This indicates that the oxycodone is released *in vivo* and is effective in inducing an analgesic effect in mice that is statistically significant from the PO gavage containing only water (Figure 5B). The analgesic time course for **20** is similar to that of oxycodone, which indicates that it can be effective in the rapid treatment of acute pain (Figure 5B).

**Figure 5:**
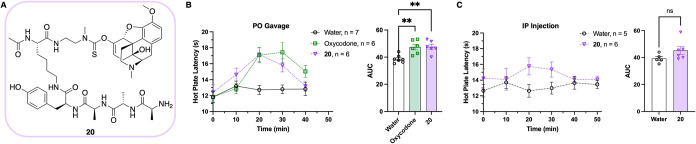
(A) Structure of oxycodone-containing prodrug **20** used for *in vivo* analgesia experiments. (B) Hot plate latency data and statistical analysis for PO gavage of **20** performed at 30 mg/kg of oxycodone in prodrug and 3 mg/kg of oxycodone. ** = *p* < 0.01. (C) Hot plate latency data and statistical analysis for IP injection of **20** performed at 30 mg/kg of oxycodone in prodrug. ns = not significant.

To confirm that this analgesic effect was due to enzymatic degradation of the prodrug in the digestive system, the antinociceptive effects of prodrug **20** were examined following systemic intraperitoneal (IP) injection. This route of administration produced withdrawal threshold latencies similar to those of the water control despite a moderate initial increase in the hot plate latency from **20** (Figure 5C). In combination with previous reports demonstrating that there is an analgesic effect with the IP injection of oxycodone,^56,57^ these results indicate that the oxycodone release is due to enzymatic cleavage of the prodrug in the stomach, corroborating the specificity for oral administration of the prodrug.

## Conclusions

We have developed an orally administered dual-enzyme-responsive peptide–oxycodone prodrug. The design of this prodrug relies on an initial passage through the acidic conditions in the stomach to activate the peptide sequence, followed by the enzymatically triggered release of free oxycodone. Improvement in the prodrug design was carried out through peptide sequence optimization to ensure the requirement of both chymotrypsin and trypsin while simultaneously enhancing the payload release kinetics. Through the preparation of a *p*NP oxycodone *O*-thionochloroformate electrophile (**5**), the ketone in oxycodone was functionalized to generate a reversible oxycodone modification that could be incorporated into a peptide scaffold. Optimized prodrug **20** was evaluated for its analgesic properties *in vivo* where it demonstrated that there was a similar effect to that of oxycodone upon oral administration of prodrug **20**. However, **20** did not alleviate acute pain upon IP injection. As misuse of oxycodone is often performed via intravenous injection, this feature of the prodrug contributes to its misuse deterrent profile. Additionally, this prodrug was stable to a broad pH range, household chemicals, and a digestive enzyme kit, demonstrating that oxycodone was not readily extracted. This observation meaningfully enhances its ability to act as a misuse deterrent prodrug.

Further development of the peptide scaffold to improve release fidelity, while retaining the dual-enzyme specificity, may allow for this prodrug to be used *in vivo* at a comparable dosage to oxycodone. Additional release assays *in vitro* could provide a more comprehensive misuse deterrence profile for the prodrug scaffold outlined herein. Further testing of **20** in postoperative or chronic pain models merits further examination to determine the potential for this prodrug to treat different types of pain. We propose that this general strategy of ketone modification for electrophile synthesis should also be amenable to other opioids and small molecule therapeutics. The fundamental chemistry and misuse deterrent opioid prodrug developed herein are anticipated to complement and advance existing efforts to combat the ongoing opioid epidemic.

## Supplementary Material



## Data Availability

All data associated with this study are present in the paper or the Supporting Information.

## Notes

Details about instruments, materials, methods, chemical synthesis, ^1^H and ^13^C­{^1^H} NMR characterization, enzymatic release assays, HPLC assays, and *in vivo* studies (PDF)
