# Supplementary material for: An Orally Administered Misuse Deterrent Opioid Prodrug for Treatment of Acute Pain
Source: JACS Au. 2025 Oct 15;5(10):5199–206. doi: 10.1021/jacsau.5c01126 (PMC12569658; doi:10.1021/jacsau.5c01126)
Supplement: Supplementary file 1 [file au5c01126_si_001.pdf]

# Supplementary Information

## An Orally Administered Misuse Deterrent Opioid Prodrug for Treatment of Acute Pain

Douglas A. Rose,[1,‡] Joseph W. Treacy,[1,‡] Karina Seth,[2] Anthony F. Tanzillo,[2] Allison Li,[2] Kyle Tamshen,[1] Lily K. Sloan,[1] Natalie Boehnke,[3] Christopher J. Evans,[2] Catherine M. Cahill,[2,*] Heather D. Maynard[1,4,*]

**Affiliations:**

[1]Department of Chemistry and Biochemistry, University of California, Los Angeles, 607 Charles E. Young Drive East, Los Angeles, California 90095-1569, United States

[2]Department of Psychiatry and Behavioral Science, Shirley and Stefan Hatos Center for Neuropharmacology, Semel Institute for Neuroscience and Human Behavior, University of California, Los Angeles, 760 Westwood Plaza, Los Angeles, California 90095-8353, United States

[3]Department of Chemical Engineering and Materials Science, University of Minnesota Twin Cities, Minneapolis, Minnesota 55455, United States

[4]California NanoSystems Institute, University of California, Los Angeles, 570 Westwood Plaza, Los Angeles, CA 90095, United States

[‡]These authors (D.A.R. and J.W.T.) contributed equally.

*Correspondence to maynard@chem.ucla.edu and cmcahill@g.ucla.edu

<u>**Table of Contents**</u>


General Experimental Details....5

Oxycodone Modification....6

Synthesis of pentafluorophenol-oxycodone thionocarbonate trifluoroacetate (**3**)....6

Synthesis of *p*NP-thionochloroformate (**4**)....9

Synthesis of *p*NP-oxycodone thionocarbonate (**5**)....11

Synthesis of *p*NP-Containing Peptides....13

Synthesis of *N*-Boc-*N*',*N*'-Cbz,Me-ethylenediamine (**21**)....13

Synthesis of *N*',*N*'-Cbz,Me-ethylenediammonium trifluoroacetate (**16**)....15

Synthesis of Fmoc-L-Lys-(2-(Cbz)methyl)ethylamino trifluoroacetate (**22**)....17

Synthesis of Fmoc-Lys[(Ac)Phe]-2-methylethylammonium trifluoroacetate (**23**)....18

Synthesis of Lys[(Ac)Phe]-(2-(*p*NP-thionocarbamate)methyl)ethylamino trifluoroacetate (**6**)19

Synthesis of Ala-Lys[(Ac)Phe]-(2-(*p*NP-thionocarbamate)methyl)ethylamino trifluoroacetate (**7**)....20

Synthesis of Ala-Ala-Lys[(Ac)Phe]-(2-(*p*NP-thionocarbamate)methyl)ethylamino trifluoroacetate (**8**)....21

Synthesis of Lys[(Boc)Phe]-(2-(Cbz)methyl)ethylamino trifluoroacetate (**24**)....22

Synthesis of Ac-Lys[Phe]-(2-(Cbz)methyl)ethylamino trifluoroacetate (**25**)....23

Synthesis of Ac-Lys[Phe-(Boc)Ala]-2-methylethylamino trifluoroacetate (**26**)....24

Synthesis of Ac-Lys[Phe-Ala]-(2-(*p*NP-thionocarbamate)methyl)ethylamino trifluoroacetate (**9**)....25

Synthesis of Ac-Lys[Phe-Ala-Ala]-(2-(*p*NP-thionocarbamate)methyl)ethylamino trifluoroacetate (**10**)....26

Synthesis of Ac-Lys[Tyr($^t$Bu)-Ala-(Fmoc)Ala] (**27**)....27

Synthesis of Ac-Lys[Tyr($^t$Bu)-Ala-(Fmoc)Ala]-2-methylethylamino trifluoroacetate (**28**)....28

Synthesis of Ac-Lys[Tyr-Ala-Ala]-(2-(pNP-thionocarbamate)methyl)ethylamino trifluoroacetate (**11**)....29

Synthesis of Ac-Lys[Tyr($^t$Bu)-Ala-Ala-(Boc)Ala] (**29**)....30

Synthesis of Ac-Lys[Tyr($^t$Bu)-Ala-Ala-(Boc)Ala]-2-methylethylamino trifluoroacetate (**30**).31

Synthesis of Ac-Lys[Tyr-Ala-Ala-Ala]-(2-(*p*NP-thionocarbamate)methyl)ethylamino trifluoroacetate (**12**)....32

Synthesis of (Fmoc)Ala-Ala-Tyr($^t$Bu) (**31**)....33

Synthesis of Ac-Ala-Lys trifluoroacetate (**32**)....34

Synthesis of Ac-Ala-Lys[Tyr($^t$Bu)-Ala-Ala-Fmoc] (**33**)....35

Synthesis of Ac-Ala-Lys[Tyr($^t$Bu)-Ala-(Fmoc)Ala]-2-methylethylamino trifluoroacetate (**34**)....36

Synthesis of Ac-Ala-Lys[Tyr-Ala-Ala]-(2-(*p*NP-thionocarbamate)methyl)ethylamino trifluoroacetate (**13**) ..... 37

Synthesis of Ac-Lys[Tyr($^t$Bu)-Ala-Ala-(Fmoc)Ala] (**15**) ..... 38

Synthesis of Ac-Lys[Tyr($^t$Bu)-Ala-Ala-(Fmoc)Ala]-2-methylethylamino trifluoroacetate (**17**) ..... 39

Synthesis of Ac-Lys[Tyr($^t$Bu)-Ala-Ala-Ala]-(2-(*p*NP-thionocarbamate)methyl)ethylamino trifluoroacetate (**14**) ..... 40

*p*NP Release Assays ..... 41

General Procedure – Release Kinetics ..... 41

General Procedure – Michaelis-Menten Kinetics ..... 41

Standard curve of *p*NP ..... 42

Release of *p*NP from **6** (Lys[(Ac)Phe]-(2-(*p*NP-thionocarbamate)methyl)ethylamino trifluoroacetate) in the presence of only chymotrypsin ..... 43

Release of *p*NP from **7** (Ala-Lys[(Ac)Phe]-(2-(*p*NP-thionocarbamate)methyl)ethylamino trifluoroacetate) in the presence of only chymotrypsin ..... 44

Release of *p*NP from **8** (Ala-Ala-Lys[(Ac)Phe]-(2-(*p*NP-thionocarbamate)methyl)ethylamino trifluoroacetate) in the presence of only chymotrypsin ..... 45

Michaelis-Menten plot of **7** (Ala-Lys[(Ac)Phe]-(2-(*p*NP-thionocarbamate)methyl)ethylamino trifluoroacetate) in the presence of chymotrypsin and trypsin ..... 46

Michaelis-Menten plot of **9** (Ac-Lys[Phe-Ala]-(2-(*p*NP-thionocarbamate)methyl)ethylamino trifluoroacetate) in the presence of chymotrypsin and trypsin ..... 47

Michaelis-Menten plot of **10** (Ac-Lys[Phe-Ala-Ala]-(2-(*p*NP-thionocarbamate)methyl)ethylamino trifluoroacetate) in the presence of chymotrypsin and trypsin ..... 48

Michaelis-Menten plot of **11** (Ac-Lys[Tyr-Ala-Ala]-(2-(pNP-thionocarbamate)methyl)ethylamino trifluoroacetate) in the presence of chymotrypsin and trypsin ..... 49

Michaelis-Menten plot of **12** (Ac-Lys[Tyr-Ala-Ala-Ala]-(2-(*p*NP-thionocarbamate)methyl)ethylamino trifluoroacetate) in the presence of chymotrypsin and trypsin ..... 50

Michaelis-Menten plot of **13** (Ac-Ala-Lys[Tyr-Ala-Ala]-(2-(*p*NP-thionocarbamate)methyl)ethylamino trifluoroacetate) in the presence of chymotrypsin and trypsin ..... 51

Release of *p*NP from **12** (Ac-Lys[Tyr-Ala-Ala-Ala]-(2-(*p*NP-thionocarbamate)methyl)ethylamino trifluoroacetate) in the presence of chymotrypsin and trypsin ..... 52

Release of *p*NP from **12** (Ac-Lys[Tyr-Ala-Ala-Ala]-(2-(*p*NP-thionocarbamate)methyl)ethylamino trifluoroacetate) in the presence of only trypsin ..... 53

Release of *p*NP from **12** (Ac-Lys[Tyr-Ala-Ala-Ala]-(2-(*p*NP-thionocarbamate)methyl)ethylamino trifluoroacetate) in the presence of only chymotrypsin..54

Release of *p*NP from **14** (Ac-Lys[Tyr($^t$Bu)-Ala-Ala-Ala]-(2-(*p*NP-thionocarbamate)methyl)ethylamino trifluoroacetate) in the presence of chymotrypsin and trypsin ....55

Release of *p*NP from **14** (Ac-Lys[Tyr($^t$Bu)-Ala-Ala-Ala]-(2-(*p*NP-thionocarbamate)methyl)ethylamino trifluoroacetate) in the presence of only trypsin ....56

Release of *p*NP from **14** (Ac-Lys[Tyr($^t$Bu)-Ala-Ala-Ala]-(2-(*p*NP-thionocarbamate)methyl)ethylamino trifluoroacetate) in the presence of only chymotrypsin..57

Synthesis of Oxycodone-Containing Prodrugs ....58

Synthesis of Ac-Lys[Tyr($^t$Bu)-Ala-Ala-Ala]-(2-(oxycodone-thionocarbamate)methyl)ethylamino ditrifluoroacetate (**18**) ....58

Synthesis of Ac-Lys[Tyr-Ala-Ala-Ala]-(2-(oxycodone-thionocarbamate)methyl)ethylamino ditrifluoroacetate (**20)** ....59

Misuse Deterrence of Prodrugs Against Common Chemicals ....60

General Procedure ....60

Household Chemical Manipulation Assay ....61

pH Misuse Deterrence Assay ....61

Synthesis of Tyrosine Containing Peptide Byproducts ....62

Synthesis of Ala-Ala-Ala-Tyr trifluoroacetate (**19**) ....62

Competitive Inhibition Release Assay ....63

General Procedure ....63

*In Vivo* Studies of Analgesic Effects ....64

General Experimental Details ....64

Hot Plate Testing ....64

Statistical Analysis ....64

*In Vivo* Plotted Data ....65

References ....66

## General Experimental Details

Oxycodone hydrochloride was supplied by Professor Christopher J. Evans and Professor Catherine M. Cahill in the UCLA Department of Neurosciences who are licensed through the Drug Enforcement Agency.

All other chemicals were used as purchased unless otherwise noted from Acros, Alfa Aesar, Chem-Impex, Combi-Blocks, Fisher Scientific, Oakwood Chemical, TCI Chemicals, or Sigma Aldrich. Dichloromethane (DCM) and triethylamine were distilled over $CaH_2$ and stored under argon. Anhydrous 1,2-dimethoxyethane (DME) was purchased and used as received. Potassium bis(trimethylsilyl)amide (KHMDS) was stored in a Vacuum Atmospheres Genesis stainless steel glove box under nitrogen atmosphere. Representative procedures are provided for each reaction. All amino acids were used as received, and all amino acids used in this study were the L-variant.

NMR spectra were obtained using either Bruker AV400, AV500, DRX500, or AV600 spectrometers and are calibrated using residual undeuterated solvent. The following abbreviations were used to explain multiplicities: s = singlet, d = doublet, t = triplet, q = quartet, m = multiplet. Flash column chromatography was performed on a Biotage Isolera One 3.0 autocolumn instrument. All silica chromatography was carried out on the Biotage using KP-Sil high-performance columns repacked using the Silicycle silica (P60, particle size 40–63 μm, column sizes described in experimental). ESI mass spectra were obtained using either a Waters Acquity LCT Premier XE equipped with an autosampler and direct injection port or an Agilent 6530 QTOF-ESI with a 1260 Infinity LC with autosampler. Analytical reverse phase HPLC was carried out on an Agilent 1260 Infinity II HPLC system equipped with an autosampler and a UV detector using a Poroshell 120 2.7 μm C18 120 Å column (Analytical: 2.7 μm, 4.6 × 100 mm) with monitoring at $\lambda$ = 220 and 254 nm and with a flow rate of 0.8 mL/min. Peptide-drug conjugates were analyzed using a mobile phase consisting of 10-100% MeCN + 0.1% TFA in water beginning with a 1 min isocratic at 10%, then up to 100% over 10 min in a linear gradient, followed by an isocratic hold at 100% MeCN + 0.1% TFA for 4 min (Total time: 15 min). Reverse phase purification was carried out on an Agilent 1290 Infinity II liquid chromatography system equipped with a UV detector using a Luna 5 μm C18 100 Å column (Preparatory: 5 μm, 250 × 21.2 mm) with monitoring at $\lambda$ = 215 and 254 nm and with a flow rate of 20 mL/min. Individual gradients for each product purified *via* reverse phase HPLC are specified in their respective procedures. All enzyme kinetic assays were performed using a SpectraMax iD3 plate reader and monitored at 405 nm.

# Oxycodone Modification

**Synthesis of pentafluorophenol-oxycodone thionocarbonate trifluoroacetate (3)**

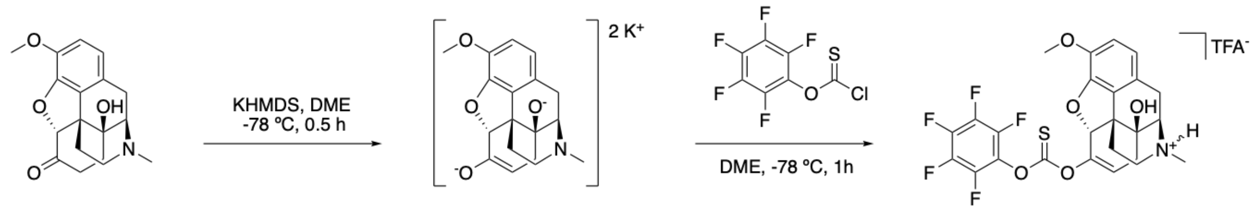


A two neck, 25 mL round bottom flask was charged with a stir bar then flame dried. Oxycodone* (45 mg, 1 Eq, 128 µmol) was dissolved in 5 mL of anhydrous DME, then the solution was cooled to -78 ºC. Anhydrous KHMDS (52 mg, 2 Eq, 256 µmol) was dissolved in 2 mL of anhydrous DME and added to the round bottom flask at -78 ºC. This was left to stir for 20 minutes under an inert atmosphere. Separately, a two neck, 25 mL round bottom flask was charged with a stir bar then flame dried, then pentafluorophenylchlorothionoformate (TCI Chemicals, 101 mg, 62 µL, 3 Eq, 384 µmol) was added to the flask and dissolved in 5 mL of anhydrous DME and subsequently cooled to -78 ºC. After 20 minutes of stirring the enolate solution, it was slowly cannula transferred (ca. five minutes) into the round bottom flask containing the electrophile. The enolate-containing flask was washed with an additional 3 mL of anhydrous DME, then this was also cannula transferred into the electrophile-containing flask. This reaction was left to stir at -78 ºC for 30 minutes after addition of the enolate, then it was stirred at 23 ºC for 30 minutes. After an hour of reaction time, the flask was concentrated under vacuum, then the resulting crude product was purified *via* preparative HPLC** (C18, 30-95% MeCN gradient against water, both with 0.1% TFA additive) to afford the product (20.2 mg, 31 µmol, 24% yield) as a white solid.

*Oxycodone was prepared from the hydrochloride salt by dissolving the hydrochloride salt in 10 mL of 0.1 M NaOH and stirring for 30 minutes. The suspension was diluted with ethyl acetate (50 mL) and transferred to a separatory funnel, then the product was extracted from the aqueous layer with ethyl acetate (3x50 mL). The organic layers were collected, dried with anhydrous magnesium sulfate, filtered, then concentrated under vacuum to yield a white solid.
**This product appears to degrade during preparative HPLC, but the oxycodone is able to be recovered if desired.

**Physical State:** White solid
**$^1$H NMR (500 MHz, $CD_3CN$):** δ 8.39 (s, 1H), 6.89 (d, *J* = 8.3 Hz, 1H), 6.81 (d, *J* = 8.3 Hz, 1H), 5.84 (dd, *J* = 6.1, 2.2 Hz, 1H), 5.27 – 5.24 (m, 1H), 3.81 (s, 4H), 3.42 – 3.35 (m, 1H), 3.25 – 3.14 (m, 2H), 2.87 (s, 3H), 2.83 – 2.73 (m, 1H), 2.67 – 2.56 (m, 1H), 2.47 – 2.36 (m, 1H), 2.27 – 2.19 (m, 1H), 1.83 – 1.75 (m, 1H).
**$^{13}$C{$^1$H} NMR (126 MHz, $CD_3CN$):** δ 191.22, 147.63, 145.28, 145.17, 143.20 – 143.01 (m), 141.18 – 141.02 (m), 140.37 – 140.09 (m), 138.64 – 137.83 (m), 129.56, 123.48, 121.32, 120.71, 116.30, 84.81, 71.36, 66.50, 57.15, 47.79, 46.68, 42.18, 32.76, 28.66, 24.59.
**$^{19}$F NMR (376 MHz, $CD_3CN$):** δ -154.27 – -154.54 (m, 2F), -158.25 (t, *J* = 20.9 Hz, 1F), -163.78 – -164.11 (m, 2F).
**HRMS (ESI/Q-TOF):** [M-TFA$^-$]$^+$ calculated for $C_{25}H_{21}F_5NO_5S^+$ 542.1055, observed 542.1142.

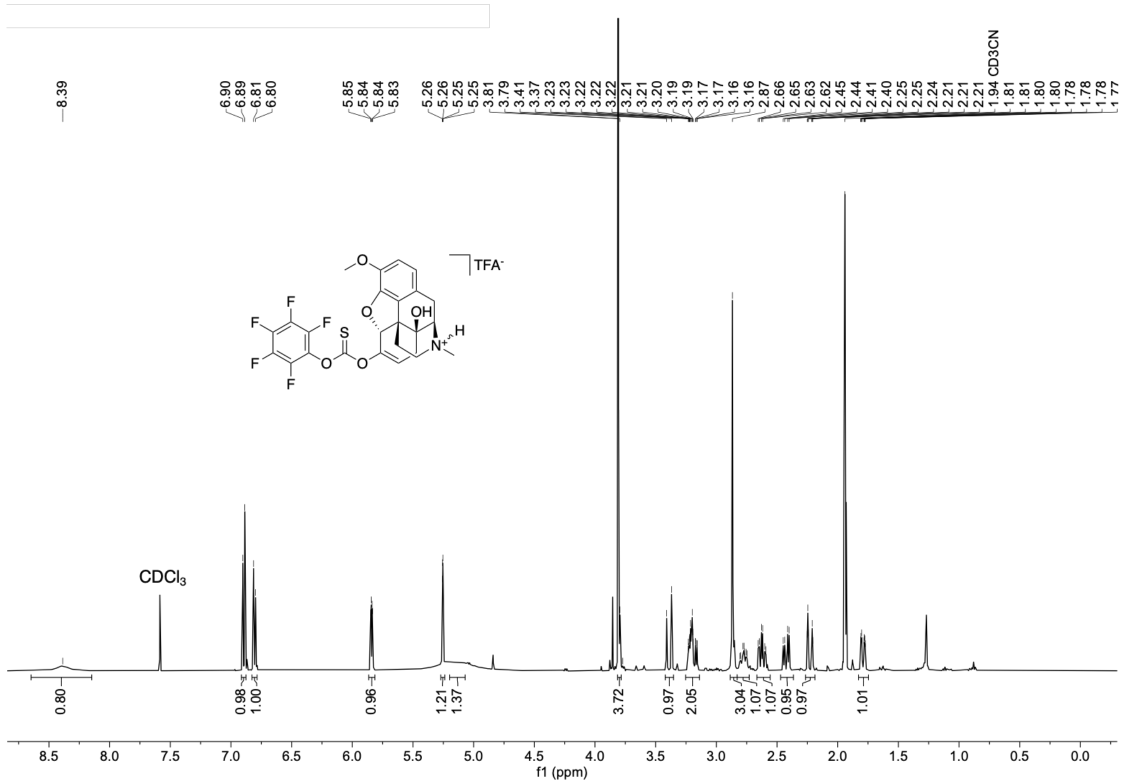

**Figure S1.** $^{1}$H NMR of **3** in $CD_3CN$ at 298 K.

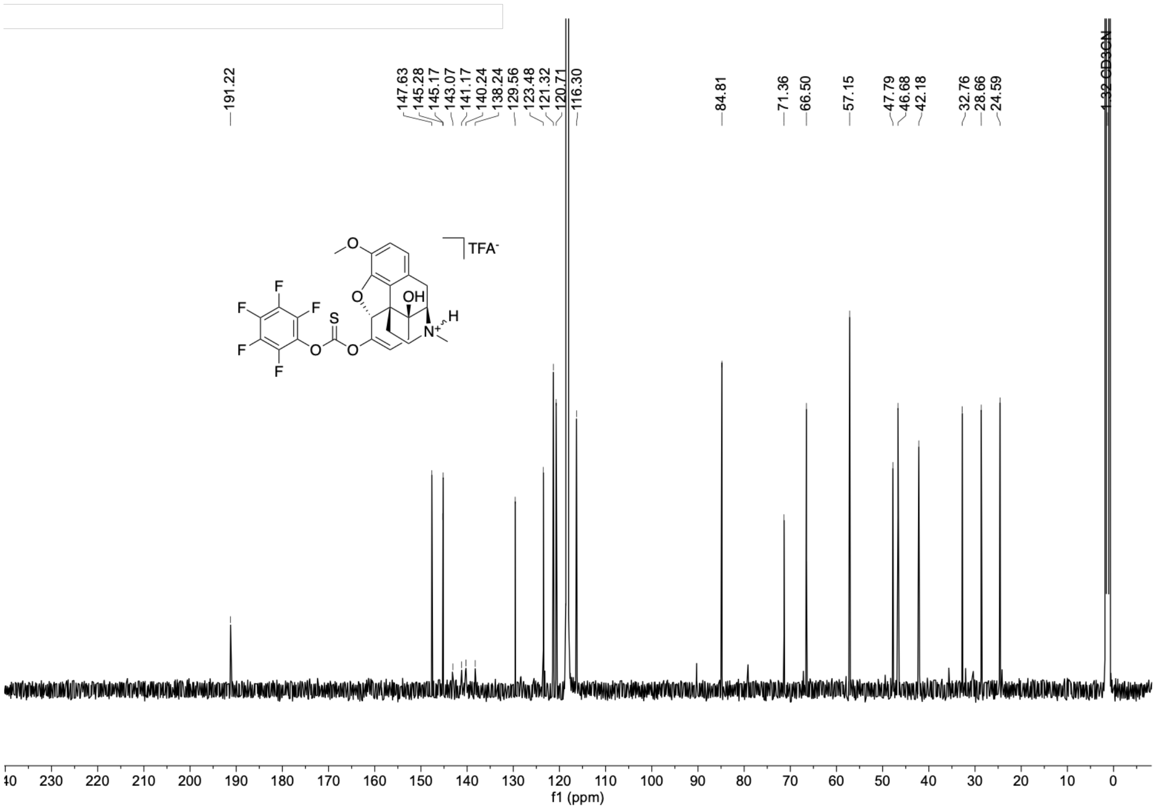

**Figure S2.** $^{13}C\{^{1}H\}$ NMR of **3** in $CD_3CN$ at 298 K.

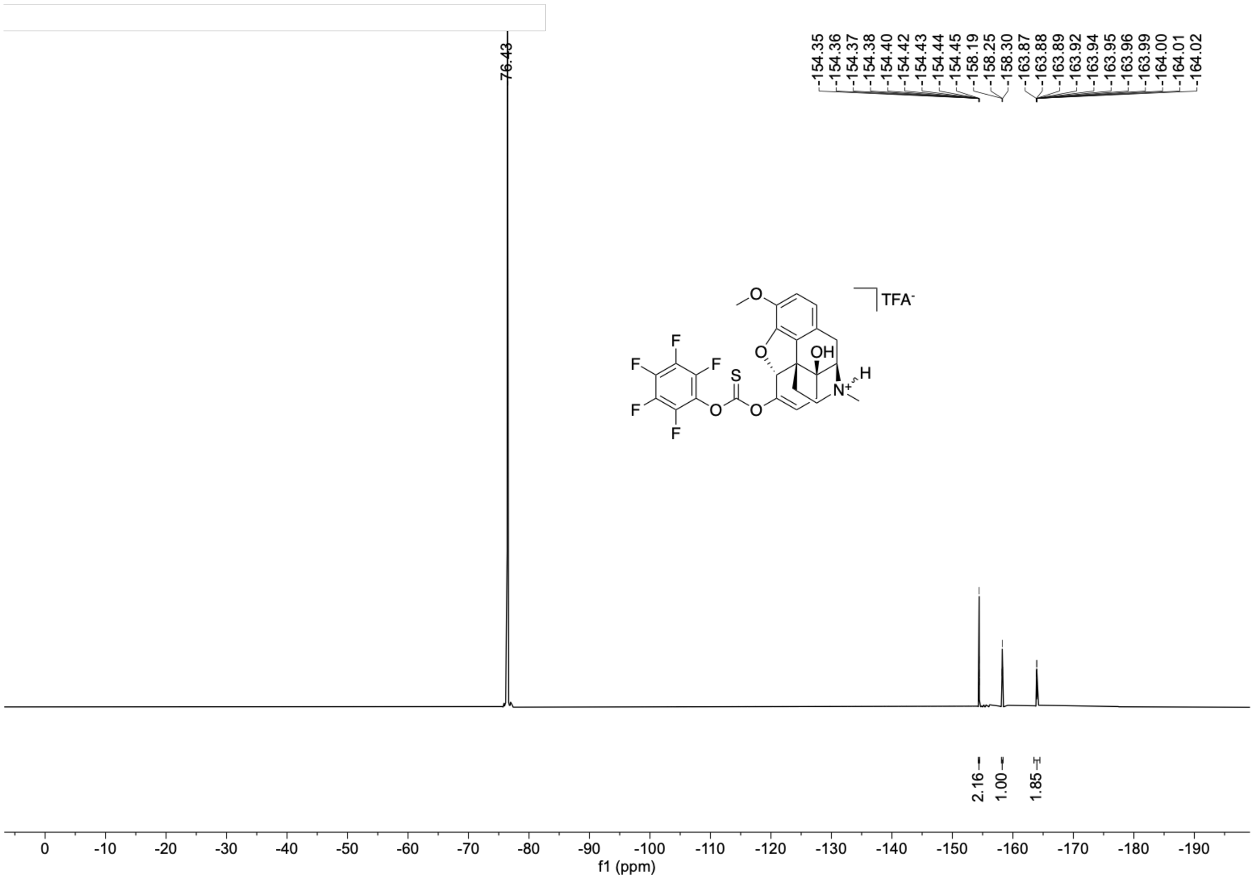


**Figure S3.** $^{19}$F NMR of **3** in $CD_3CN$ at 298 K. The peak at -76.43 ppm is from the trifluoroacetate counterion.

**Synthesis of *p*NP-thionochloroformate (4)**

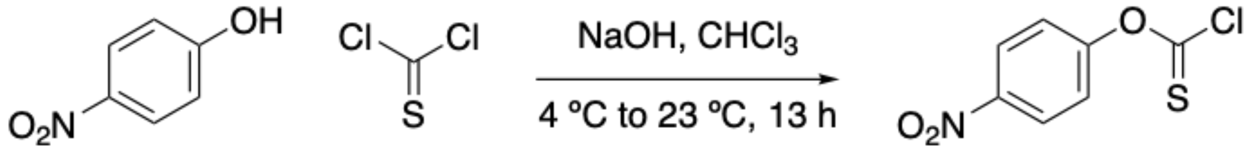


A solution of 4-nitrophenol (2.00 g, 1 Eq, 14.4 mmol) in 0.5 M sodium hydroxide (575 mg, 28.8 mL, 0.500 molar, 1 Eq, 14.4 mmol) was prepared in a 250 mL round bottom flask charged with a stir bar and cooled to 4 °C. Separately, a solution of thiophosgene (1.1 mL, 1 Eq, 14.4 mmol) in $CHCl_3$ (20 mL) was prepared and then added dropwise to the cooled solution *via* syringe pump over the course of 10 minutes. This solution was then stirred at 4 °C for 1 hour, then warmed up to 23 °C, and stirred for an additional 12 hours at 23 °C. The chloroform was removed under vacuum, and the resulting suspension was transferred to a separatory funnel using EtOAc (300 mL). The organic layer was then washed with saturated aqueous sodium bicarbonate (3 x 75 mL), brine (50 mL), dried over anhydrous magnesium sulfate, filtered, and concentrated under vacuum. This was further purified by flash column chromatography (50 g silica gel, 5-50% EtOAc gradient against hexanes) to afford the product (1.84 g, 8.496 mmol, 59% yield) as a yellow solid.

**Physical State:** Yellow solid
**TLC (UV):** $R_f$ 0.73 (9:1 hexanes-ethyl acetate).
**$^1$H NMR (600 MHz, $CDCl_3$):** δ 8.37 – 8.34 (m, 2H), 7.36 – 7.33 (m, 2H).
**$^{13}$C{$^1$H} NMR (126 MHz, $CDCl_3$):** δ 184.78, 158.27, 146.60, 125.91, 122.83.
**HRMS (ESI/Q-TOF):** $[M+H]^+$ calculated for $C_7H_5ClNO_3S^+$ 217.9679, observed 217.9758.

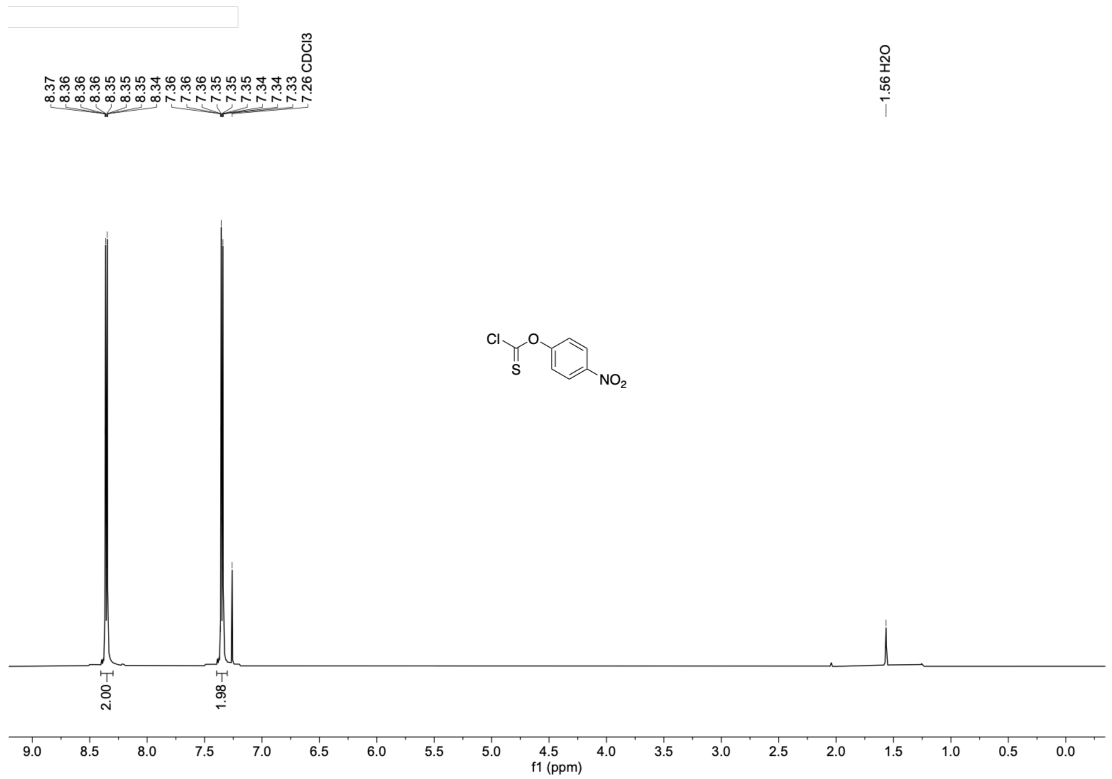


**Figure S4.** $^{1}$H NMR of **4** in $CDCl_3$ at 298 K.

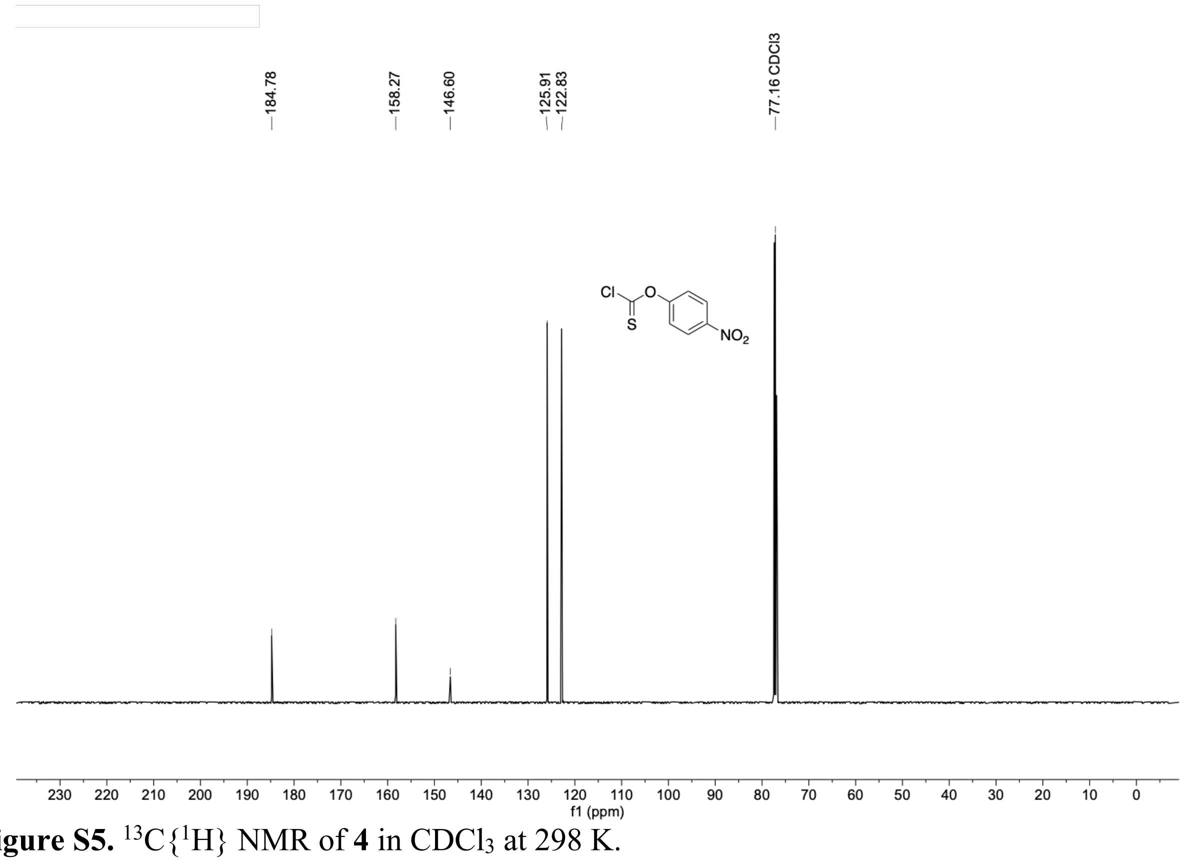


**Figure S5.** $^{13}$C{$^{1}$H} NMR of **4** in $CDCl_3$ at 298 K.

**Synthesis of *p*NP-oxycodone thionocarbonate (5)**

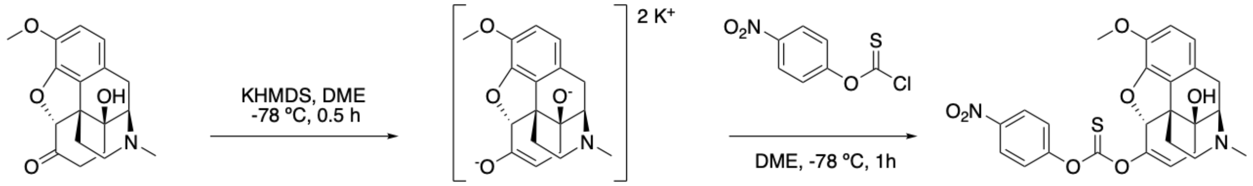


A two neck, 25 mL round bottom flask was charged with a stir bar then flame dried. Oxycodone* (45 mg, 1 Eq, 128 µmol) was dissolved in 5 mL of anhydrous DME, then the solution was cooled to -78 ºC. Anhydrous KHMDS (52 mg, 2 Eq, 256 µmol) was dissolved in 2 mL of anhydrous DME and added to the round bottom flask at -78 ºC. This was left to stir for 20 minutes under an inert atmosphere. Separately, a two neck, 25 mL round bottom flask was charged with a stir bar then flame dried, then **4** (44 mg, 1.6 Eq, 205 µmol) was added to the flask and dissolved in 5 mL of anhydrous DME and subsequently cooled to -78 ºC. After 20 minutes of stirring the enolate solution, it was slowly cannula transferred (ca. five minutes) into the round bottom flask containing the electrophile. The enolate-containing flask was washed with an additional 3 mL of anhydrous DME, then this was also cannula transferred into the electrophile-containing flask. This reaction was left to stir at -78 ºC for 30 minutes after addition of the enolate, then it was stirred at 23 ºC for 30 minutes. After an hour of reaction time, the flask was concentrated under vacuum, then the reaction was transferred to a separatory funnel with ethyl acetate (200 mL). The organic layer was washed three times with water (3 x 50 mL), then once with brine (50 mL). The organic layers were combined, dried with anhydrous magnesium sulfate, filtered, and concentrated under vacuum. The yellow solid was then dissolved in ca. 1 mL of DCM and precipitated into chilled hexanes (45 mL, ca. -20 ºC). This was repeated two more times, then the solid was concentrated under vacuum to afford the product (46 mg, 93 µmol, 73% yield) as a pale yellow solid. This material is 90% pure by $^{1}$H NMR and was not further purified as not to decrease the yield of the oxycodone-containing product.**

*Oxycodone was prepared from the hydrochloride salt by dissolving the hydrochloride salt in 10 mL of 0.1 M NaOH and stirring for 30 minutes. The suspension was diluted with ethyl acetate (50 mL) and transferred to a separatory funnel, then the product was extracted from the aqueous layer with ethyl acetate (3x50 mL). The organic layers were collected, dried with anhydrous magnesium sulfate, filtered, then concentrated under vacuum to yield a white solid.
**It is possible to recover additional product by collecting the supernatant, concentrating it under vacuum, and performing additional precipitations in hexanes.

**Physical State:** Pale yellow solid
**$^{1}$H NMR (500 MHz, $CDCl_3$):** δ 8.35 – 8.28 (m, 2H), 7.39 – 7.34 (m, 2H), 6.74 (d, *J* = 8.2 Hz, 1H), 6.68 (d, *J* = 8.2 Hz, 1H), 5.74 (dd, *J* = 6.1, 2.2 Hz, 1H), 5.23 (s, 1H), 3.85 (s, 3H), 3.25 – 3.10 (m, 2H), 2.80 – 2.71 (m, 1H), 2.70 – 2.61 (m, 1H), 2.52 (s, 3H), 2.48 – 2.30 (m, 3H), 2.26 – 2.17 (m, 1H), 1.69 (dd, *J* = 12.8, 3.4 Hz, 1H).
**$^{13}$C{$^{1}$H} NMR (126 MHz, $CDCl_3$):** δ 192.00, 157.66, 146.65, 146.08, 144.65, 143.92, 130.43, 125.49, 124.82, 123.40, 120.48, 119.37, 114.31, 85.40, 70.60, 64.31, 56.67, 47.04, 45.57, 42.99, 31.91, 30.42, 22.72.
**HRMS (ESI/Q-TOF):** $[M+H]^+$ calculated for $C_{25}H_{25}N_2O_7S^+$ 497.1382, observed 497.1447.

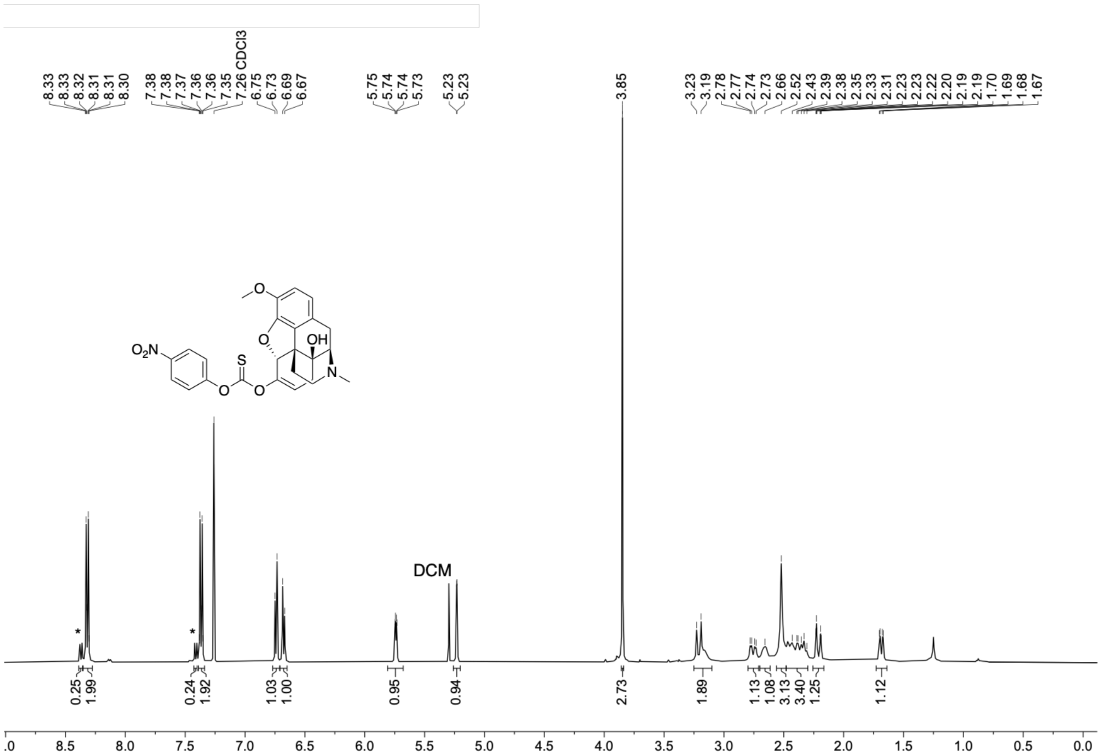

**Figure S6.** $^{1}H$ NMR of **5** in $CDCl_3$ at 298 K. Peaks indicated by * correspond to excess **4** that was not removed by precipitation.

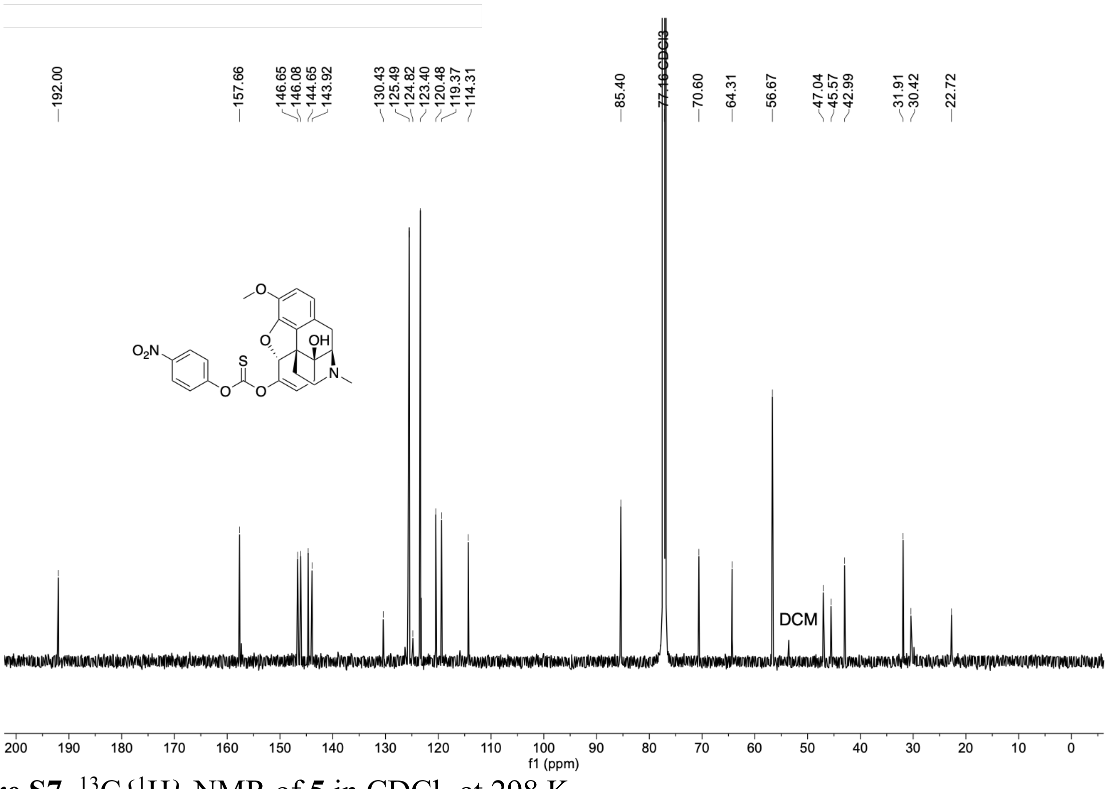

**Figure S7.** $^{13}C\{^{1}H\}$ NMR of **5** in $CDCl_3$ at 298 K.

# Synthesis of *p*NP-Containing Peptides

**Synthesis of *N*-Boc-*N*',*N*'-Cbz,Me-ethylenediamine (21)**

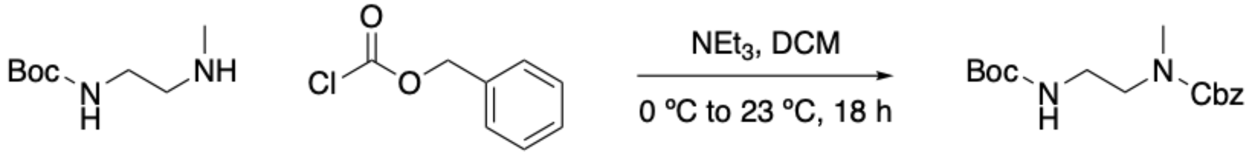


A two neck, 250 mL flame dried round bottom flask was charged with a stir bar *tert*-Butyl-2-(methylamino)ethylcarbamate (1.100 g, 1 Eq, 6.313 mmol) was added to the flask and dissolved in 50 mL of anhydrous DCM, forming a pale yellow solution. Anhydrous triethylamine (1.022 g, 1.41 mL, 1.6 Eq, 10.10 mmol) was added to the reaction with no observable exotherm or color change. The flask was cooled to 0 ºC under an argon atmosphere. Separately, carbobenzoxy chloride, Cbz-Cl, (1.131 g, 1.002 mL, 1.05 Eq, 6.629 mmol) was dissolved in 20 mL of anhydrous DCM, then this was added to the round bottom flask over thirty minutes *via* syringe pump. The flask was left to warm up to 23 ºC overnight under an argon atmosphere. After 18 hours, the reaction was transferred to a separatory funnel (150 mL DCM) where the organic layer was washed once with brine (75 mL). The organic layer was collected, dried with anhydrous magnesium sulfate, filtered, and concentrated under vacuum. The material was further purified using flash column chromatography (100 g silica gel, 0-10% methanol against DCM) to afford the product (1.693 g, 5.490 mmol, 87% yield) as a yellow oil.

**Physical State:** Yellow oil
**TLC (UV):** $R_f$ 0.48 (9:1 DCM-methanol).
**$^1$H NMR (600 MHz, $CD_3CN$):** δ 7.42 – 7.23 (m, 5H), 5.34 (s, 1H), 5.08 (s, 2H), 3.35 – 3.29 (m, 2H), 3.17 (q, $J$ = 6.2 Hz, 2H), 2.93 – 2.85 (m, 3H), 1.38 (s, 9H).
**$^{13}$C{$^1$H} NMR (151 MHz, $CD_3CN$):** δ 156.94, 138.48, 129.43, 128.78, 128.54, 79.25, 67.46, 49.54, 49.19, 39.26, 35.40, 28.61.
**HRMS (ESI/Q-TOF):** $[M+Na]^+$ calculated Mass for $C_{16}H_{24}N_2NaO_4^+$ 331.1634, observed 331.1619.

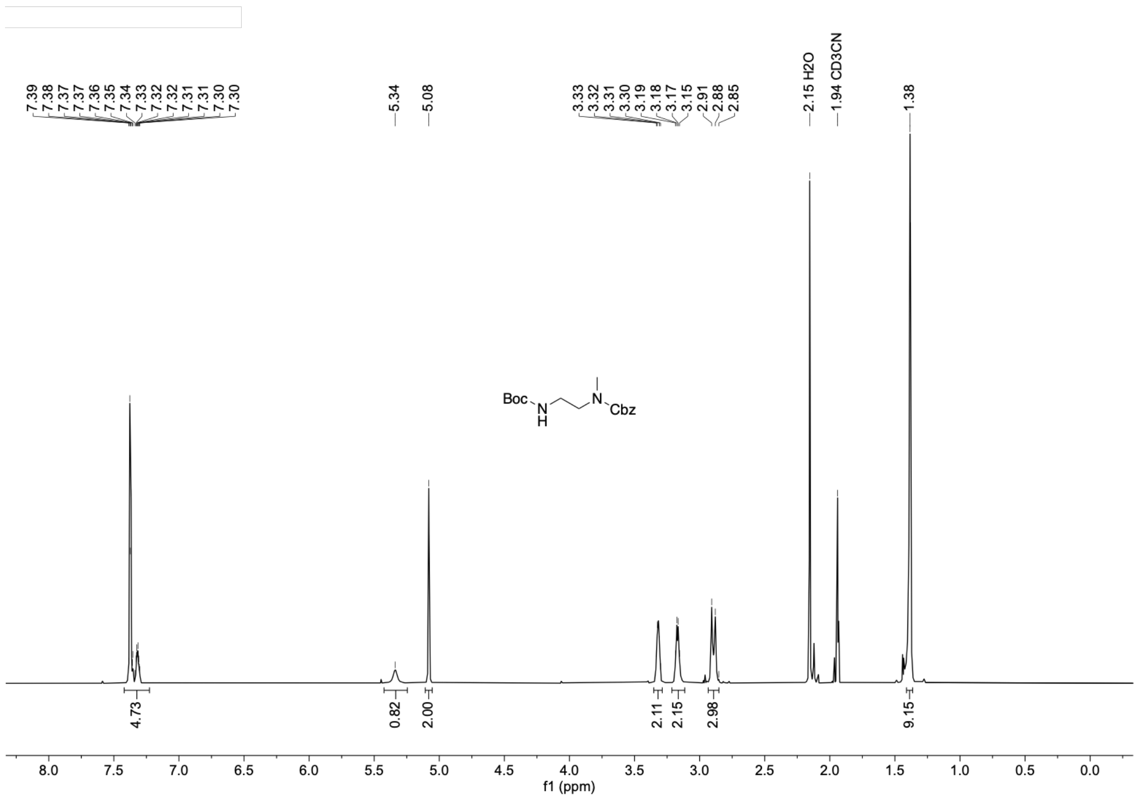


**Figure S8.** $^{1}H$ NMR of **21** in $CD_3CN$ at 298 K.

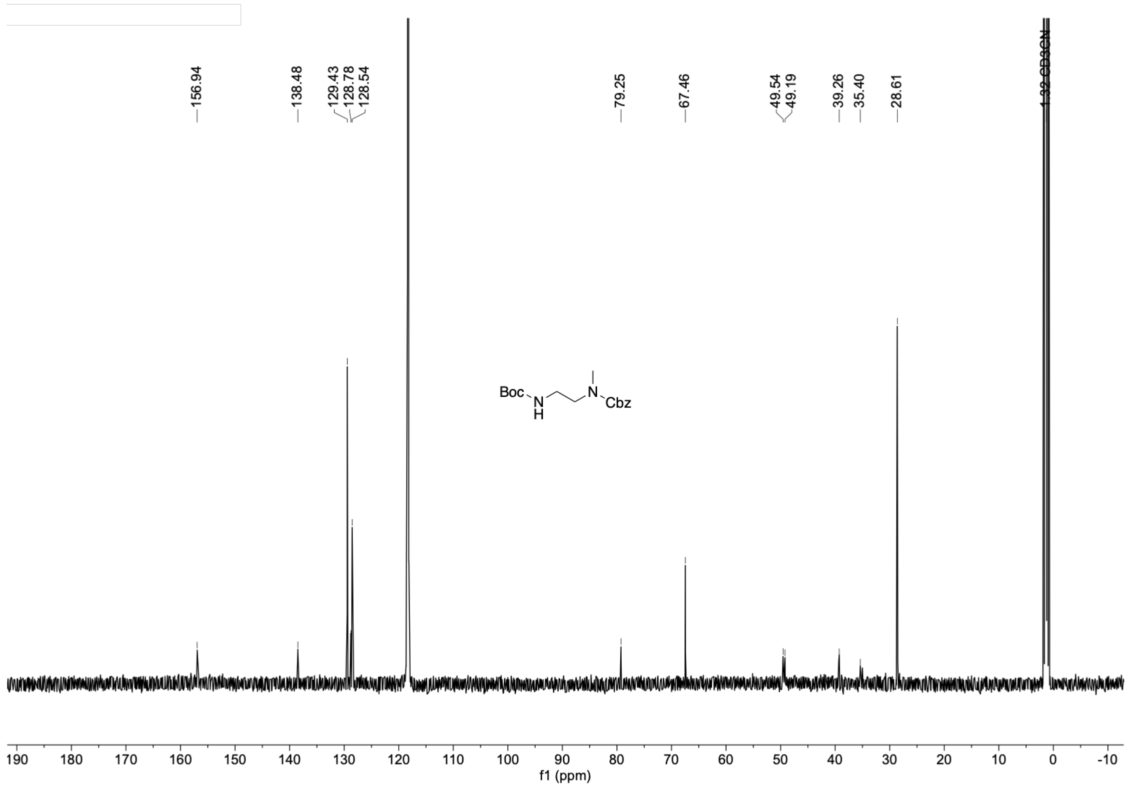


**Figure S9.** $^{13}C\{^{1}H\}$ NMR of **21** in $CD_3CN$ at 298 K.

**Synthesis of *N'*,*N'*-Cbz,Me-ethylenediammonium trifluoroacetate (16)**

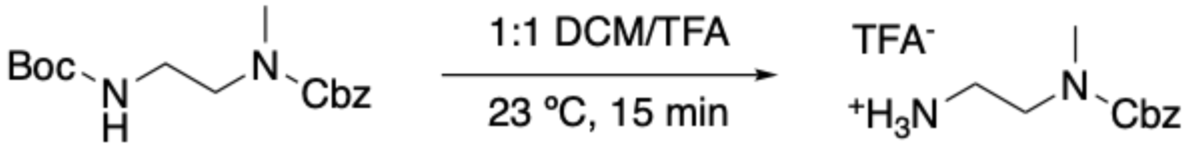


**21** (650 mg, 1 Eq, 2.11 mmol) was dissolved in 5 mL of DCM in a scintillation vial charged with a stir bar, creating a clear solution. Trifluoroacetic acid (TFA) (7.40 g, 5.00 mL, 30.8 Eq, 64.9 mmol) was added to the solution, forming a light yellow color with gas evolution. This was stirred at 23 ºC for 15 minutes. After 15 minutes, the reaction was concentrated under vacuum. The resulting yellow oil was triturated twice with diethyl ether (2 x 20 mL), forming a white suspension. The white suspension was centrifuged down, and the clear supernatant was decanted to afford the product (615 mg, 1.91 mmol, 91% yield) as a white solid.

**Physical State:** White solid
**$^1$H NMR (600 MHz, $CD_3CN$):** δ 7.43 – 7.27 (m, 5H), 5.11 (s, 2H), 3.61 – 3.47 (m, 2H), 3.20 – 3.05 (m, 2H), 2.98 – 2.84 (m, 3H).
**$^{13}$C{$^1$H} NMR (151 MHz, $CD_3CN$):** δ 161.73, 137.96, 129.47, 128.94, 128.73, 68.13, 47.75, 39.38, 35.18.
**HRMS (ESI/Q-TOF):** $[M+H]^+$ calculated for $C_{11}H_{17}N_2O_2^+$ 209.1290, observed 209.1239.

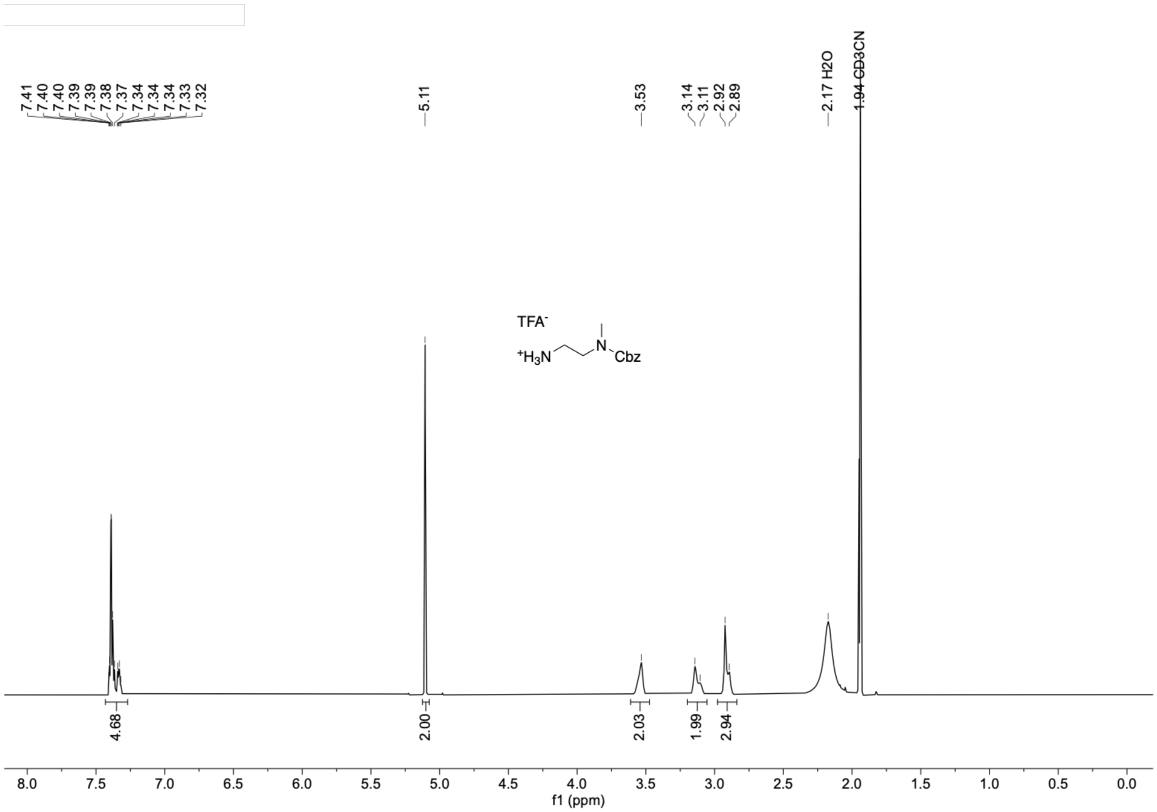


**Figure S10.** $^{1}H$ NMR of **16** in $CD_3CN$ at 298 K.

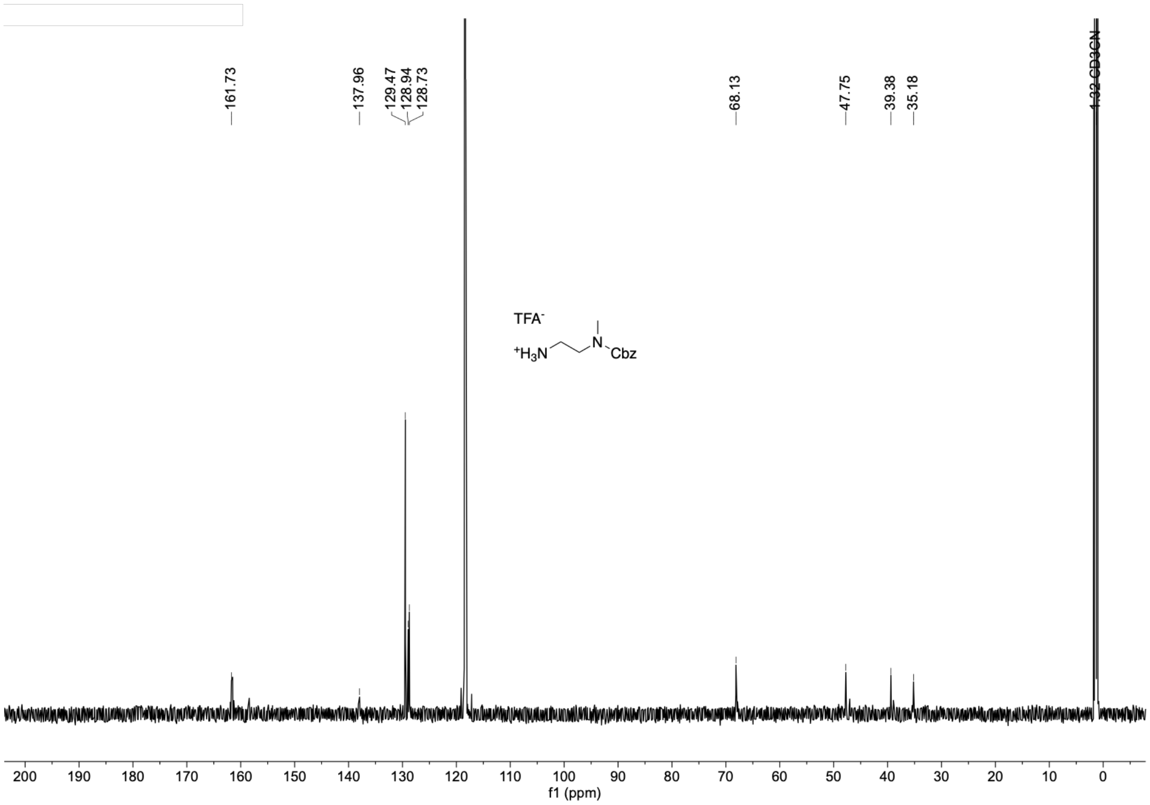


**Figure S11.** $^{13}C\{^{1}H\}$ NMR of **16** in $CD_3CN$ at 298 K.

**Synthesis of Fmoc-L-Lys-(2-(Cbz)methyl)ethylamino trifluoroacetate (22)**

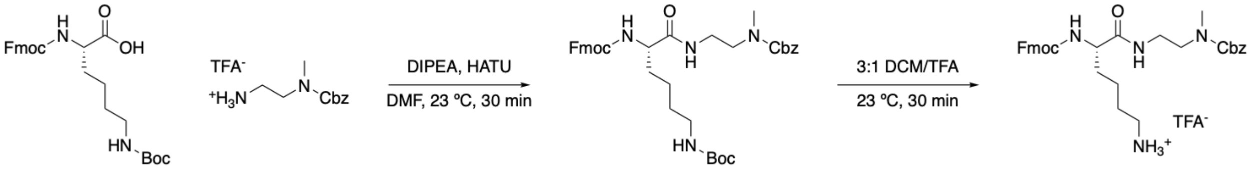


To a solution of Fmoc-Lys(Boc)-OH (400.0 mg, 1 Eq, 854 µmol) in DMF (5 mL) was added HATU (357 mg, 1.1 Eq, 939 µmol) and DIPEA (410 µL, 3 Eq, 2.56 mmol), forming a bright yellow solution. This solution was stirred for 15 minutes to ensure complete activation. Separately, a solution of **16** (303.0 mg, 1.1 Eq, 939 µmol) was prepared in DMF (2 mL) and then added dropwise to the activated peptide. This solution was stirred for 30 minutes at 23 °C, then the reaction was concentrated under vacuum and precipitated into water (45 mL) to afford the intermediate as a white solid. This was carried forward without any further purification.

**HRMS (ESI/Q-TOF):** $[M+H]^+$ calculated for $C_{37}H_{47}N_4O_7^+$ 659.3445, observed 659.2946.

The resulting peptide was dissolved in a 25% TFA solution in DCM (v/v, 10 mL). This was stirred at 23 °C for 30 minutes and then concentrated under vacuum. The resulting crude product was then purified *via* preparative HPLC (C18, 10-50% MeCN gradient against water, both with 0.1% TFA additive) to afford the product (432 mg, 641 µmol, 75% yield) as a white solid.

**HRMS (ESI/Q-TOF):** $[M\text{-}TFA^-]^+$ calculated for $C_{32}H_{39}N_4O_5^+$ 559.2920, observed 559.3320.

**Synthesis of Fmoc-Lys[(Ac)Phe]-2-methylethylammonium trifluoroacetate (23)**

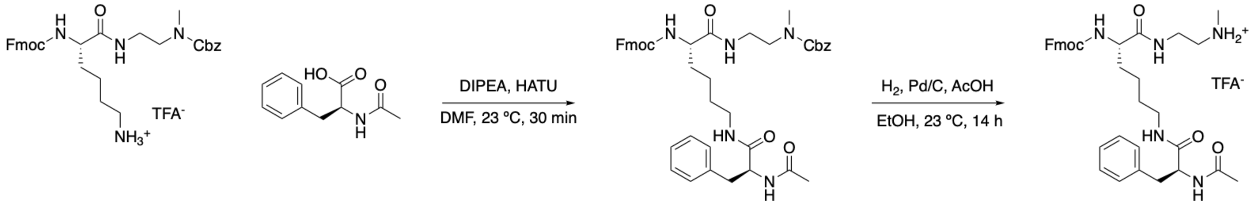


To a solution of Ac-Phe-OH (72.5 mg, 1.1 Eq, 350 µmol) in DMF (5 mL) was added HATU (133 mg, 1.1 Eq, 350 µmol) and DIPEA (166 µL, 3 Eq, 0.95 mmol), forming a bright yellow solution. This solution was stirred for 15 minutes to ensure complete activation. Separately, a solution of **22** (214 mg, 1 Eq, 318 µmol) was prepared in DMF (2 mL) and then added dropwise to the activated peptide. This solution was stirred for 30 minutes at 23 °C, then the reaction was concentrated under vacuum and precipitated into water (45 mL) to afford the intermediate as a white solid. This was carried forward without any further purification.

**HRMS (ESI/Q-TOF):** $[M+H]^+$ calculated for $C_{43}H_{50}N_5O_7^+$ 748.3710, observed 748.4602.

The resulting peptide was then dissolved in ethanol (20 mL) with acetic acid additive* (401 µL, 20 Eq, 7.00 mmol) and combined with Pd/C (30 mg, 10 wt. % Pd, 0.1 Eq, 35 µmol). This solution was then sparged with argon, sealed under an atmosphere of hydrogen with a balloon, and stirred at 23 °C for 14 hours. The reaction was then filtered over Celite and concentrated under vacuum. The resulting crude product was then purified *via* preparative HPLC (C18, 10-100% MeCN gradient against water, both with 0.1% TFA additive) to afford the product (92 mg, 127 µmol, 40% yield) as a white solid.

*Acetic acid was used as an additive to protonate the secondary amine that is formed after the hydrogenolysis of the Cbz group. This formation of the ammonium salt prevents significant deprotection of the Fmoc group by the secondary amine.

**HRMS (ESI/Q-TOF):** $[M\text{-}TFA^-]^+$ calculated for $C_{35}H_{44}N_5O_5^+$ 614.3337, observed 614.3444.

**Synthesis of Lys[(Ac)Phe]-(2-(*p*NP-thionocarbamate)methyl)ethylamino trifluoroacetate (6)**

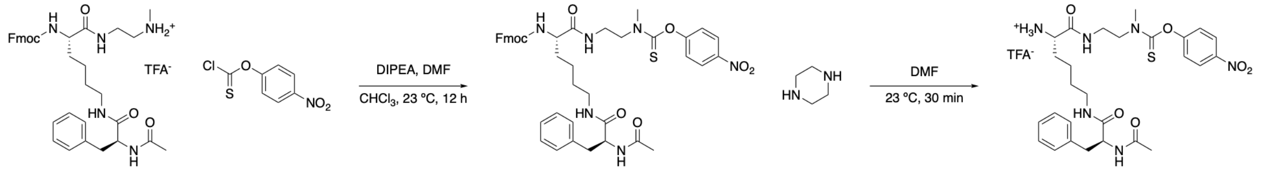


To a solution of **23** (92 mg, 1 Eq, 127 µmol) in $CHCl_3$ (2.5 mL) and DMF (0.5 mL) was added DIPEA (66 µL, 3 Eq, 381 µmol). This solution was stirred for five minutes before a solution of **4** (35 mg, 1.25 Eq, 159 µmol) in $CHCl_3$ (1.0 mL) was added. This yellow solution was stirred at 23 °C for 12 hours. The reaction was concentrated under vacuum and then precipitated into water (45 mL) to afford the product as a pale yellow solid. This was carried forward without any further purification.

**HRMS (ESI/Q-TOF):** $[M+H]^+$ calculated for $C_{42}H_{47}N_6O_8S^+$ 795.3176, observed 795.3326.

The resulting peptide was dissolved in DMF (2 mL) with piperazine* (110 mg, 10 Eq, 1.27 mmol). This was stirred at 23 °C for 30 minutes, then the reaction was concentrated under vacuum. The resulting crude product was then purified *via* preparative HPLC (C18, 10-50% MeCN gradient against water, both with 0.1% TFA additive) to afford the product (44 mg, 64 µmol, 51% yield) as a white solid.

*Other secondary amines may be used to carry out this deprotection, however, the Fmoc-4-methylpiperidine adduct produced after deprotection had a similar retention time on preparative HPLC to the desired product. Accordingly, piperazine was used to shift the retention time of the Fmoc-amine adduct to allow for improved separation and easier purification.

**HRMS (ESI/Q-TOF):** $[M\text{-}TFA^-]^+$ calculated for $C_{27}H_{37}N_6O_6S^+$ 573.2490, observed 573.2581.

**Synthesis of Ala-Lys[(Ac)Phe]-(2-(*p*NP-thionocarbamate)methyl)ethylamino trifluoroacetate (7)**

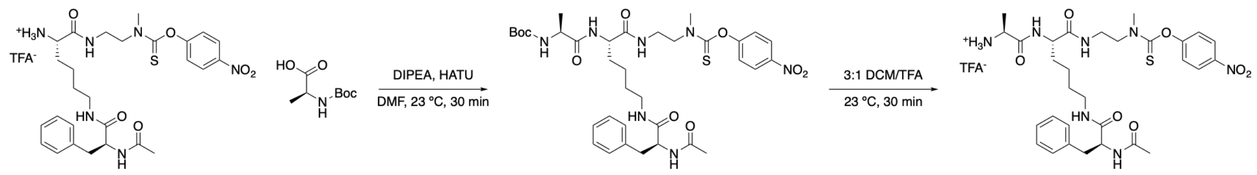


To a solution of Boc-Ala-OH (31 mg, 1.5 Eq, 170 µmol) in DMF (5 mL) was added HATU (63 mg, 1.5 Eq, 170 µmol) and DIPEA (96 µL, 3.5 Eq, 0.55 mmol), forming a bright yellow solution. This solution was stirred for 15 minutes to ensure complete activation. Separately, a solution of **6** (76 mg, 1 Eq, 110 µmol) was prepared in DMF (2 mL) and then added dropwise to the activated peptide. This solution was stirred for 30 minutes at 23 °C, then the reaction was concentrated under vacuum and then precipitated into water (45 mL) to afford the intermediate as a white solid. This was carried forward without any further purification.

**HRMS (ESI/Q-TOF):** $[M+H]^+$ calculated for $C_{35}H_{50}N_7O_9S^+$ 744.3391, observed 744.3480.

The resulting peptide was dissolved in a 25% TFA solution in DCM (v/v, 10 mL). This was stirred at 23 °C for 30 minutes and then concentrated under vacuum. The resulting crude product was then purified *via* preparative HPLC (C18, 10-80% MeCN gradient against water, both with 0.1% TFA additive) to afford the product (62 mg, 81 µmol, 74% yield) as a white solid.

**HRMS (ESI/Q-TOF):** $[M\text{-}TFA^-]^+$ calculated for $C_{30}H_{42}N_7O_7S^+$ 644.2861, observed 644.2968.

**Synthesis of Ala-Ala-Lys[(Ac)Phe]-(2-(*p*NP-thionocarbamate)methyl)ethylamino trifluoroacetate (8)**

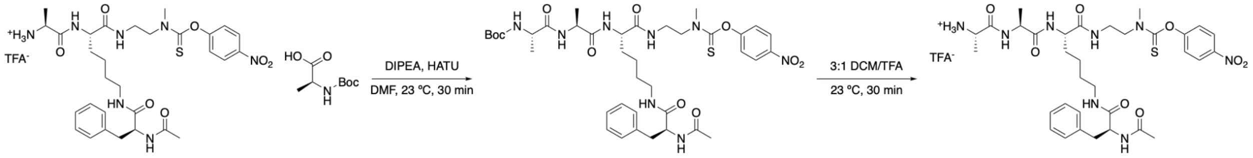


To a solution of Boc-Ala-OH (16 mg, 1.5 Eq, 85 μmol) in DMF (5 mL) was added HATU (32 mg, 1.5 Eq, 85 μmol) and DIPEA (49 μL, 3.5 Eq, 298 μmol mmol), forming a bright yellow solution. This solution was stirred for 15 minutes to ensure complete activation. Separately, a solution of **7** (43 mg, 1 Eq, 57 μmol) was prepared in DMF (2 mL) and then added dropwise to the activated peptide. This solution was stirred for 30 minutes at 23 °C, then the reaction was concentrated under vacuum and precipitated into water (45 mL) to afford the intermediate as a white solid. This was carried forward without any further purification.

**HRMS (ESI/Q-TOF):** $[M+H]^+$ calculated for $C_{38}H_{55}N_8O_{10}S^+$ 815.3762, observed 815.3869.

The resulting peptide was dissolved in a 25% TFA solution in DCM (v/v, 10 mL). This was stirred at 23 °C for 30 minutes and then concentrated under vacuum. The resulting crude product was then purified *via* preparative HPLC (C18, 10-80% MeCN gradient against water, both with 0.1% TFA additive) to afford the product (43 mg, 52 μmol, 91% yield) as a white solid.

**HRMS (ESI/Q-TOF):** $[M\text{-}TFA^-]^+$ calculated for $C_{33}H_{47}N_8O_8S^+$ 715.3232, observed 715.3345.

**Synthesis of Lys[(Boc)Phe]-(2-(Cbz)methyl)ethylamino trifluoroacetate (24)**

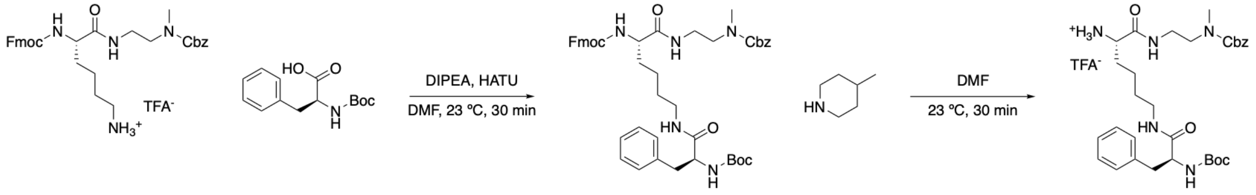


To a solution of Boc-Phe-OH (475 mg, 2 Eq, 1.79 mmol) in DMF (5 mL) was added HATU (681 mg, 2 Eq, 1.79 mmol) and DIPEA (780 µL, 5 Eq, 4.48 mmol) forming a bright yellow solution. This solution was stirred for 15 minutes to ensure complete activation. Separately, a solution of **22** (500 mg, 1 Eq, 0.90 mmol) was prepared in DMF (2 mL) and then added dropwise to the activated peptide. This solution was stirred for 30 minutes at 23 °C, then the reaction was concentrated under vacuum and precipitated into water (45 mL) to afford the intermediate as a white solid. This was carried forward without any further purification.

The resulting peptide was dissolved in a 20% 4-methylpiperidine solution in DMF (v/v, 5 mL). This was stirred at 23 °C for 30 minutes and then concentrated under vacuum. The resulting crude product was then purified *via* preparative HPLC (C18, 10-100% MeCN gradient against water, both with 0.1% TFA additive) to afford the product (380 mg, 567 µmol, 63% yield) as a white solid.

**HRMS (ESI/Q-TOF):** $[M\text{-}TFA^-]^+$ calculated for $C_{31}H_{46}N_5O_6^+$ 584.3443, observed 584.3550.

**Synthesis of Ac-Lys[Phe]-(2-(Cbz)methyl)ethylamino trifluoroacetate (25)**

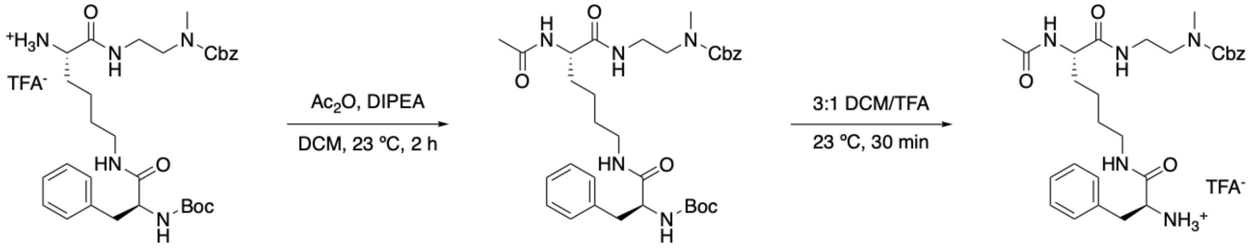


To a solution of **24** (380 mg, 1 Eq, 567 µmol) in DCM (15 mL) was added DIPEA (593 µL, 6 Eq, 3.40 mmol) and acetic anhydride (193 µL, 4 Eq, 2.04 mmol). This solution was stirred for 2 hours at 23 °C, then the reaction was concentrated under vacuum and precipitated into a 1:1 solution of diethyl ether and hexanes (45 mL) to afford the intermediate as a white solid. This was carried forward without any further purification.

The resulting peptide was dissolved in a 25% TFA solution in DCM (v/v, 10 mL), which was stirred at 23 °C for 30 minutes and then concentrated under vacuum. The resulting crude product was then purified *via* preparative HPLC (C18, 10-80% MeCN gradient against water, both with 0.1% TFA additive) to afford the product (232 mg, 369 µmol, 65% yield) as a white solid.

**HRMS (ESI/Q-TOF):** $[M-TFA^-]^+$ calculated for $C_{28}H_{40}N_5O_5^+$ 526.3024, found 526.3006.

**Synthesis of Ac-Lys[Phe-(Boc)Ala]-2-methylethylamino trifluoroacetate (26)**

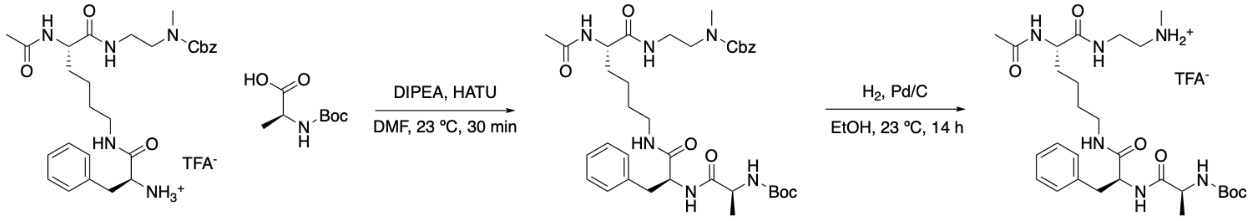


To a solution of Boc-Ala-OH (100 mg, 1.4 Eq, 530 µmol) in DMF (5 mL) was added HATU (202 mg, 1.4 Eq, 530 µmol) and DIPEA (385 µL, 6 Eq, 2.27 mmol) forming a bright yellow solution. This solution was stirred for 15 minutes to ensure complete activation. Separately, a solution of **25** (232 mg, 1 Eq, 369 µmol) was prepared in DMF (2 mL) and then added dropwise to the activated peptide. This solution was stirred for 30 minutes at 23 °C, then the reaction was concentrated under vacuum and precipitated into water (45 mL) to afford the intermediate as a white solid. This was carried forward without any further purification.

The resulting peptide was dissolved in ethanol (30 mL) and combined with Pd/C (40 mg, 10 wt. % Pd, 0.1 Eq, 53 µmol). This solution was then sparged with argon, sealed under an atmosphere of hydrogen with a balloon, and stirred at 23 °C for 14 hours. The reaction was then filtered over Celite and concentrated under vacuum. The resulting crude product was then purified *via* preparative HPLC (C18, 10-85% MeCN gradient against water, both with 0.1% TFA additive) to afford the product (120 mg, 177 µmol, 48% yield) as a white solid.

**HRMS (ESI/Q-TOF):** $[M\text{-}TFA^-]^+$ calculated for $C_{28}H_{47}N_6O_6^+$ 563.3552, observed 563.3320.

**Synthesis of Ac-Lys[Phe-Ala]-(2-(*p*NP-thionocarbamate)methyl)ethylamino trifluoroacetate (9)**

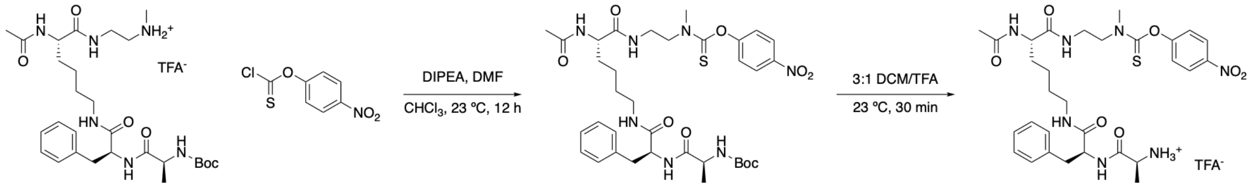


To a solution of **26** (35.0 mg, 1 Eq, 51.5 μmol) in $CHCl_3$ (2.5 mL) and DMF (0.5 mL) was added DIPEA (43.4 μL, 5 Eq, 249 μmol). This solution was stirred for five minutes before a solution of **4** (20.3 mg, 1.8 Eq, 93.4 μmol) in $CHCl_3$ (1.0 mL) was added, forming a bright yellow solution. This solution was then vigorously stirred at 23 °C for 12 hours, then the reaction was concentrated under vacuum and precipitated into water (45 mL) to afford the intermediate as a pale yellow solid. This was carried forward without any further purification.

The resulting peptide was dissolved in a 25% TFA solution in DCM (v/v, 10 mL), which was stirred at 23 °C for 30 minutes and then concentrated under vacuum. The resulting crude product was then purified *via* preparative HPLC (C18, 10-100% MeCN gradient against water, both with 0.1% TFA additive) to afford the product (30.0 mg, 38.6 μmol, 75% yield) as a white solid.

**HRMS (ESI/Q-TOF):** $[M\text{-}TFA^-]^+$ calculated for $C_{30}H_{42}N_7O_7S^+$ 644.2861, observed 644.2857.

**Synthesis of Ac-Lys[Phe-Ala-Ala]-(2-(*p*NP-thionocarbamate)methyl)ethylamino trifluoroacetate (10)**

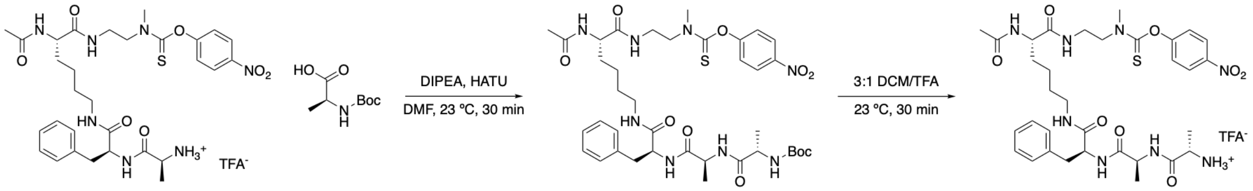


To a solution of Boc-Ala-OH (9.5 mg, 1.5 Eq, 50.4 µmol) in DMF (2 mL) was added HATU (19.2 mg, 1.5 Eq, 50.4 µmol) and DIPEA (37 µL, 6 Eq, 210 µmol), forming a bright yellow solution. This solution was stirred for 15 minutes to ensure complete activation. Separately, a solution of **9** (27.0 mg, 1 Eq, 33.1 µmol) was prepared in DMF (1 mL) and then added dropwise to the activated peptide. This solution was stirred for 30 minutes at 23 °C, then the reaction was concentrated under vacuum and precipitated into water (45 mL) to afford the intermediate as a white solid. This was carried forward without any further purification.

The resulting peptide was dissolved in a 25% TFA solution in DCM (v/v, 10 mL), which was stirred at 23 °C for 30 minutes and then concentrated under vacuum. The resulting crude product was then purified *via* preparative HPLC (C18, 10-90% MeCN gradient against $H_2O$, both with 0.1% TFA additive) to afford the product (13.6 mg, 15.2 µmol, 46% yield) as a white solid.

**HRMS (ESI/Q-TOF):** $[M\text{-}TFA^-]^+$ calculated for $C_{33}H_{47}N_8O_8S^+$ 715.3232, observed 715.3348.

**Synthesis of Ac-Lys[Tyr($^t$Bu)-Ala-(Fmoc)Ala] (27)**

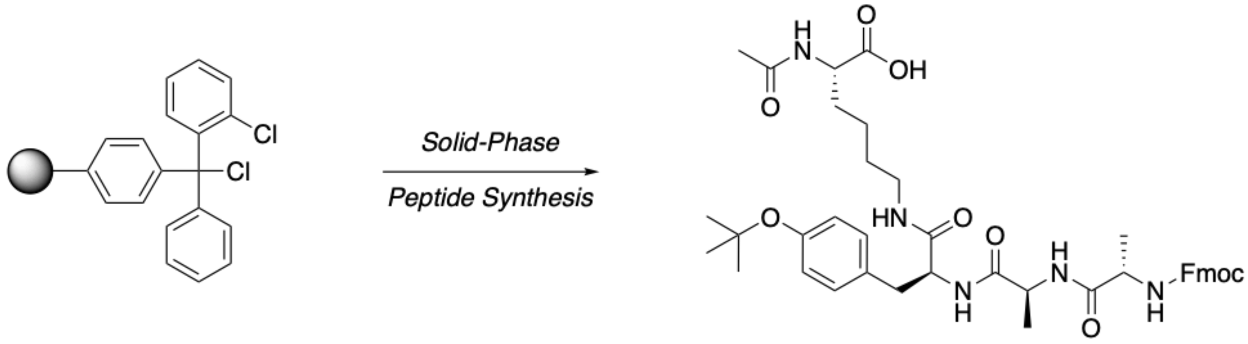


The peptide was synthesized *via* standard Fmoc Solid-Phase Peptide Synthesis conditions. The peptide was prepared using 1.000 g of 2-chlorotrityl chloride resin (0.89 mmol/g ChemImpex). The resin was swelled in DCM for 60 minutes prior to any modifications. The initial lysine coupling was carried out using two equivalents of Ac-Lys(Fmoc)-OH and six equivalents of DIPEA. This was done in 12 mL of 1:1 DCM and NMP (v/v) mixture and shaken for 90 minutes. Each amino acid residue thereafter was loaded using a 3:3:6 equivalent ratio of Fmoc-Amino Acid: HATU: DIPEA in NMP for 30 minutes (Equivalents relative to resin). The couplings were followed by deprotection in 20% 4-methylpiperidine in DMF (v/v, ca. 8 mL) for 20 minutes. After coupling the second alanine residue, the Fmoc protecting group was not cleaved. The resin was dried under vacuum and then swelled in DCM for 40 minutes. The peptide was cleaved from the resin while maintaining the *tert*-butyl protecting group by using a 20% HFIP in DCM (v/v) cleavage cocktail (40 mL). Approximately 10 mL of the cleavage cocktail was added to the resin/peptide and mixed for two minutes. The resin was filtered and the flow through was collected, then the solvent was removed under vacuum. This process was repeated three additional times. After each removal of HFIP and DCM under vacuum, the concentrate was precipitated into chilled diethyl ether (45 mL, ca. -20 ºC) to afford the product (387 mg, 501 µmol, 56% yield) as a white solid.

**HRMS (ESI/Q-TOF):** $[M+H]^+$ calculated for $C_{42}H_{54}N_5O_9^+$ 772.3922, observed 772.3973.

**Synthesis of Ac-Lys[Tyr($^t$Bu)-Ala-(Fmoc)Ala]-2-methylethylamino trifluoroacetate (28)**

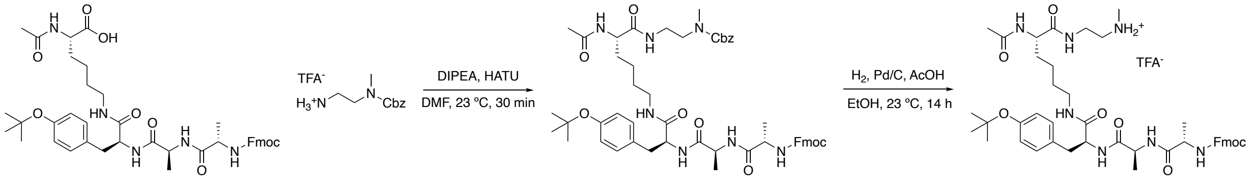


To a solution of **27** (379 mg, 1 Eq, 491 µmol) in DMF (10 mL) was added HATU (224 mg, 1.2 Eq, 589 µmol) and DIPEA (428 µL, 5 Eq, 2.45 mmol), forming a bright yellow solution. This solution was stirred for 15 minutes to ensure complete activation. Separately, a solution of **16** (153 mg, 1.5 Eq, 737 µmol) was prepared in DMF (2 mL) and then added dropwise to the activated peptide. This solution was stirred for 30 minutes at 23 °C, then the reaction was concentrated under vacuum and precipitated into water (45 mL) to afford the intermediate as a white solid. This was carried forward without any further purification.

The resulting peptide was dissolved in ethanol (30 mL) with acetic acid additive* (562 µL, 20 Eq, 9.82 mmol) and combined with Pd/C (84 mg, 10 wt. % Pd, 0.1 Eq, 49 µmol). This solution was then sparged with argon, sealed under an atmosphere of hydrogen with a balloon, and stirred at 23 °C for 14 hours. The reaction was then filtered over Celite and concentrated under vacuum. The crude product was then purified *via* preparative HPLC (C18, 10-75% MeCN gradient against water, both with 0.1% TFA additive) to afford the product (141 mg, 150 µmol, 31% yield) as a white solid.

*Acetic acid was used as an additive to protonate the secondary amine that is formed after the hydrogenolysis of the Cbz group. This formation of the ammonium salt prevents significant deprotection of the Fmoc group by the secondary amine.

**HRMS (ESI/Q-TOF):** $[M-TFA^-]^+$ calculated for $C_{45}H_{62}N_7O_8^+$ 828.4654, observed 828.4782.

**Synthesis of Ac-Lys[Tyr-Ala-Ala]-(2-(pNP-thionocarbamate)methyl)ethylamino trifluoroacetate (11)**

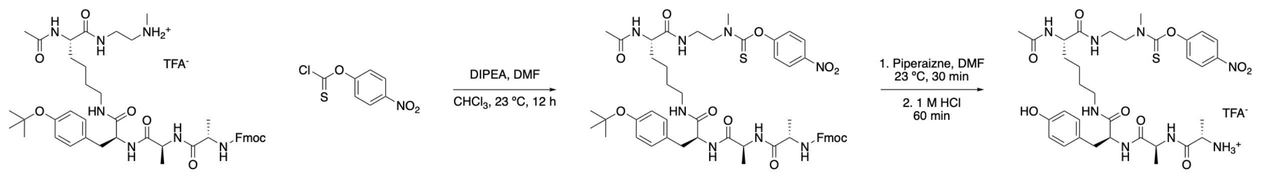


To a solution of **28** (25.0 mg, 1 Eq, 32.4 µmol) in $CHCl_3$ (2.5 mL) and DMF (0.5 mL) was added DIPEA (17.0 µL, 3 Eq, 97 µmol). This solution was stirred for five minutes before a solution of **4** (10.6 mg, 1.5 Eq, 48.6 µmol) in $CHCl_3$ (1.0 mL) was added. This solution was then vigorously stirred at 23 °C for 12 hours, then the reaction was concentrated under vacuum and precipitated into water (45 mL) to afford the intermediate as a pale yellow solid. This was carried forward without any further purification.

The resulting peptide was dissolved in DMF (2 mL) with piperazine (67.0 mg, 10 Eq, 780 µmol). This was stirred at 23 °C for 30 minutes and then concentrated under vacuum. The resulting crude product was then acidified using 1 M aq. HCl and stirred for an additional 60 minutes, then the material was purified *via* preparative HPLC (10-100% MeCN gradient against water, both with a 0.1% TFA additive) to afford the product (14.6 mg, 17.3 µmol, 54% yield) as a white solid.

**HRMS (ESI/Q-TOF):** $[M\text{-}TFA^-]^+$ calculated for $C_{33}H_{47}N_8O_9S^+$ 731.3181, observed 731.3244.

**Synthesis of Ac-Lys[Tyr($^t$Bu)-Ala-Ala-(Boc)Ala] (29)**

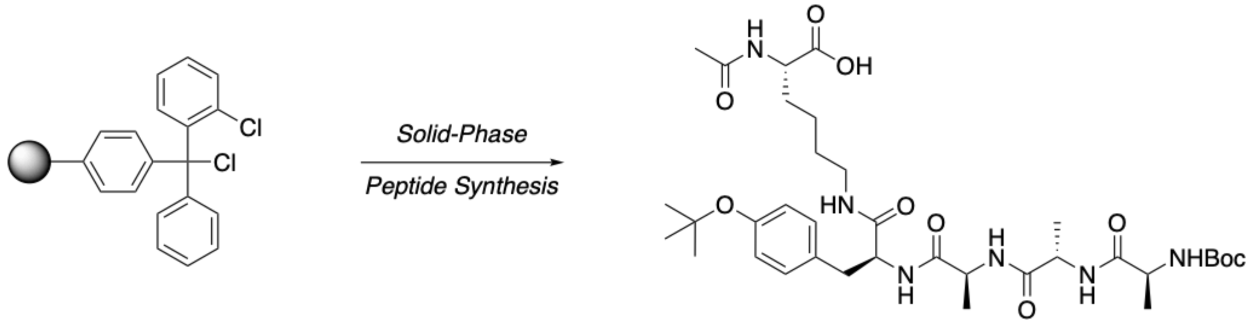


The peptide was synthesized *via* standard Fmoc Solid-Phase Peptide Synthesis conditions. The peptide was prepared using 1.000 g of 2-chlorotrityl chloride resin (0.89 mmol/g ChemImpex). The resin was swelled in DCM for 60 minutes prior to any modifications. The initial lysine coupling was carried out using two equivalents of Ac-Lys(Fmoc)-OH and six equivalents of DIPEA. This was done in 12 mL of 1:1 DCM and NMP (v/v) mixture and shaken for 90 minutes. Each amino acid residue thereafter was loaded using a 3:3:6 equivalent ratio of Fmoc- or Boc-Amino Acid: HATU: DIPEA in NMP for 30 minutes (Equivalents relative to resin). The couplings were followed by deprotection in 20% 4-methylpiperidine in DMF (v/v, ca. 8 mL) for 20 minutes. After coupling the third alanine residue, the Boc protecting group was not cleaved. The resin was dried under vacuum and then swelled in DCM for 40 minutes. The peptide was cleaved from the resin while maintaining the Boc and *tert*-butyl protecting groups by using a 20% HFIP in DCM (v/v) cleavage cocktail (40 mL). Approximately 10 mL of the cleavage cocktail was added to the resin/peptide and mixed for two minutes. The resin was filtered and the flow through was collected, then the solvent was removed under vacuum. This process was repeated three additional times. After each removal of HFIP and DCM under vacuum, the concentrate was precipitated into chilled diethyl ether (45 mL, ca. -20 ºC) to afford the product (540 mg, 749 µmol, 75% yield) as a white solid.

**HRMS (ESI/Q-TOF):** $[M+H]^+$ calculated for $C_{35}H_{57}N_6O_{10}^+$ 721.4131, observed 721.3825.

**Synthesis of Ac-Lys[Tyr($^t$Bu)-Ala-Ala-(Boc)Ala]-2-methylethylamino trifluoroacetate (30)**

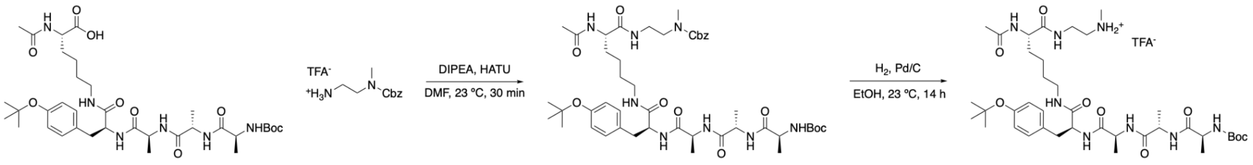


To a solution of **29** (540 mg, 1 Eq, 749 µmol) in DMF (10 mL) was added HATU (314 mg, 1.1 Eq, 824.5 µmol) and DIPEA (392 µL, 3 Eq, 2.249 mmol) forming a bright yellow solution. This solution was stirred for 15 minutes to ensure complete activation. Separately, a solution of **16** (266 mg, 1.1 Eq, 824.5 µmol) was prepared in DMF (2 mL) and then added dropwise to the activated peptide. This solution was stirred for 30 minutes at 23 °C, then the reaction contents were concentrated under vacuum and precipitated into water (45 mL) to afford the intermediate as a white solid. This was carried forward without any further purification.

The product was then dissolved in ethanol (30 mL) and combined with Pd/C (80 mg, 10 wt. %, Pd, 0.1 Eq, 75 µmol). This solution was then sparged with argon, sealed under an atmosphere of hydrogen, and stirred at 23 °C for 14 hours. The reaction was then filtered over Celite and concentrated under vacuum. The crude product was then purified *via* preparative HPLC (10-75% MeCN gradient against water, both with a 0.1% TFA additive) to afford the product (355 mg, 399 µmol, 53% yield) as a white solid.

**HRMS (ESI/Q-TOF):** $[M\text{-}TFA^-]^+$ calculated for $C_{38}H_{65}N_8O_9^+$ 777.4869, observed 777.4898.

**Synthesis of Ac-Lys[Tyr-Ala-Ala-Ala]-(2-(*p*NP-thionocarbamate)methyl)ethylamino trifluoroacetate (12)**

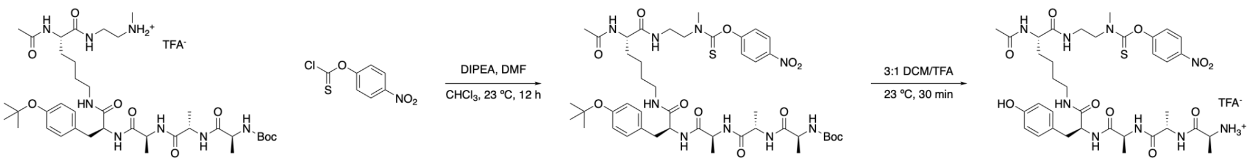

To a solution of **30** (25.0 mg, 1 Eq, 27.8 µmol) in $CHCl_3$ (2.5 mL) and DMF (0.5 mL) was added DIPEA (17.0 µL, 3 Eq, 83 µmol). This solution was stirred for five minutes before a solution of **4** (8.5 mg, 1.4 Eq, 39.0 µmol) in $CHCl_3$ (1.0 mL) was added. This solution was then vigorously stirred at 23 °C for 12 hours. The reaction was concentrated under vacuum and then precipitated into water (45 mL) to afford the intermediate as a pale yellow solid. This was carried forward without any further purification.

This resulting peptide was dissolved in DCM (3 mL) with TFA (1 mL). This was stirred at 23 °C for 30 minutes and then concentrated under vacuum. The resulting crude product was then purified *via* preparative HPLC (10-100% MeCN gradient against water, both with a 0.1% TFA additive) to afford the product (12.8 mg, 14.0 µmol, 50% yield) as a white solid.

**HRMS (ESI/Q-TOF):** $[M\text{-}TFA^-]^+$ calculated for $C_{36}H_{52}N_9O_{10}S^+$ 802.3552, observed 802.3570.

**Synthesis of (Fmoc)Ala-Ala-Tyr($^t$Bu) (31)**

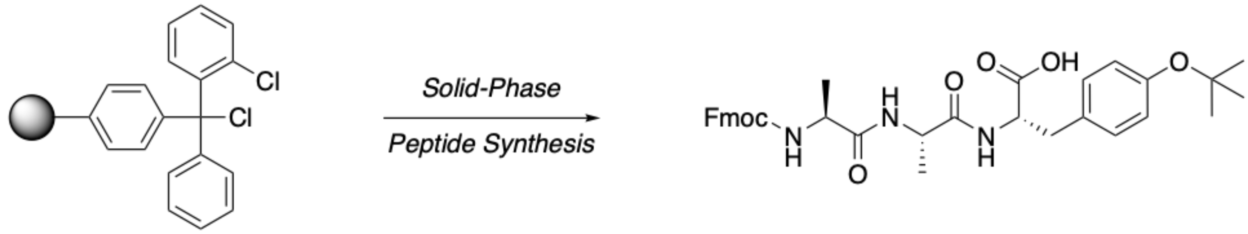


The peptide was synthesized *via* standard Fmoc Solid-Phase Peptide Synthesis conditions. The peptide was prepared using 1.000 g of 2-chlorotrityl chloride resin (0.89 mmol/g ChemImpex). The resin was swelled in DCM for 60 minutes prior to any modifications. The initial tyrosine coupling was carried out using two equivalents of Fmoc-Tyrosine($^t$Bu)-OH and six equivalents of DIPEA. This was done in 12 mL of 1:1 DCM and NMP (v/v) and shaken for 90 minutes. Each amino acid residue thereafter was loaded using a 3:3:6 equivalent ratio of Fmoc-Amino Acid: HATU: DIPEA in NMP for 30 minutes. The couplings were followed by deprotection in 20% 4-methylpiperidine in DMF (v/v, ca. 8 mL) for 20 minutes. After coupling the second alanine residue, the Fmoc protecting group was not cleaved. The resin was dried under vacuum for 120 minutes and then swelled in DCM for 40 minutes. The peptide was cleaved from the resin while maintaining the Fmoc and *tert*-butyl protecting groups by using a 20% HFIP in DCM (v/v) cleavage cocktail (40 mL). Approximately 10 mL of the cleavage cocktail was added to the resin/peptide and mixed for two minutes. The resin was filtered and the flow through was collected, then the solvent was removed under vacuum. This process was repeated three additional times. After each removal of HFIP and DCM under vacuum, the concentrate was precipitated into chilled diethyl ether (45 mL, ca. -20 ºC) to afford the product (455 mg, 756 µmol, 76% yield) as a white solid.

**HRMS (ESI/Q-TOF):** $[M+H]^+$ calculated for $C_{34}H_{40}N_3O_7^+$ 602.2866, observed 602.2903.

**Synthesis of Ac-Ala-Lys trifluoroacetate (32)**

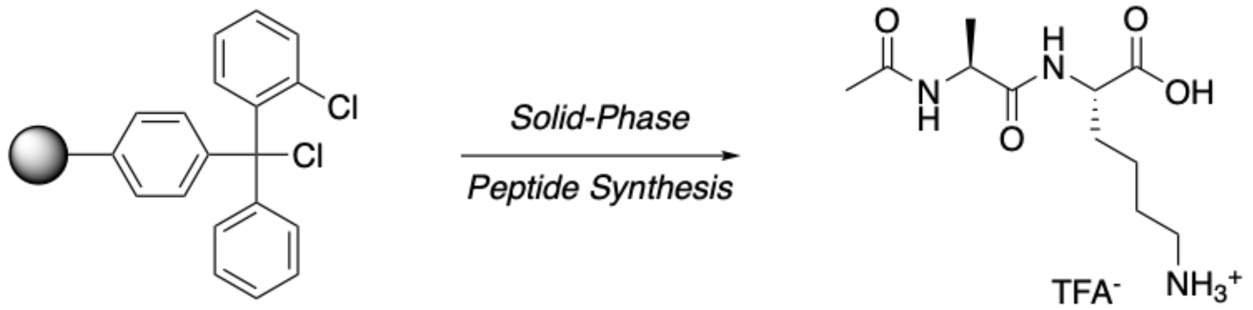


The peptide was synthesized *via* standard Fmoc Solid-Phase Peptide Synthesis conditions. The peptide was prepared using 1.000 g of 2-chlorotrityl chloride resin (0.89 mmol/g ChemImpex). The resin was swelled in DCM for 60 minutes prior to any modifications. The initial lysine coupling was carried out using two equivalents of Fmoc-Lysine(Boc)-OH and six equivalents of DIPEA. This was done in 12 mL of 1:1 DCM and NMP (v/v) and shaken for 90 minutes. Each amino acid residue thereafter was loaded using a 3:3:6 equivalent ratio of Fmoc-Amino Acid: HATU: DIPEA in NMP for 30 minutes. The couplings were followed by deprotection in 20% 4-methylpiperidine in DMF (v/v, ca. 8 mL) for 20 minutes. The resin was dried under vacuum for 120 minutes and then swelled in DCM for 40 minutes. The peptide was cleaved from the resin while simultaneously removing the Boc protecting groups by using a 20% TFA in DCM (v/v) cleavage cocktail (40 mL). Approximately 10 mL of the cleavage cocktail was added to the resin/peptide and mixed for two minutes. The resin was filtered and the flow through was collected, then the solvent was removed under vacuum. After each removal of TFA and DCM under vacuum the concentrate was precipitated into chilled diethyl ether (45 mL, ca. -20 ºC) to afford the product (268 mg, 717 µmol, 72% yield) as a white solid.

**HRMS (ESI/Q-TOF):** $[M\text{-}TFA^-]^+$ calculated for $C_{11}H_{22}N_3O_4^+$ 260.1605, observed 260.1669.

**Synthesis of Ac-Ala-Lys[Tyr(^tBu)-Ala-Ala-Fmoc] (33)**

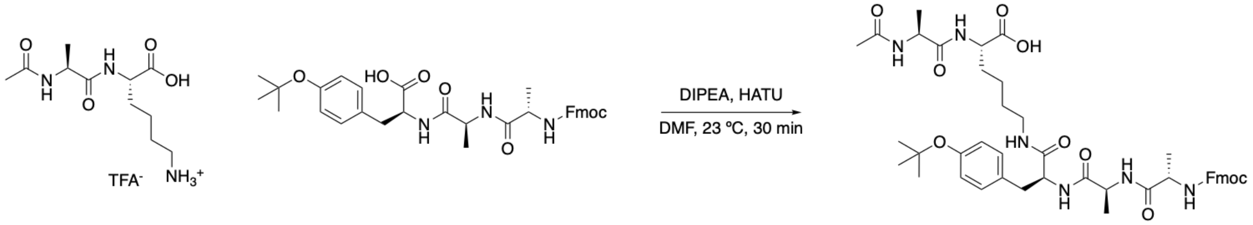


To a solution of **31** (443 mg, 1.1 Eq, 737 µmol) in DMF (5 mL) was added HATU (280 mg, 1.1 Eq, 737 µmol) and DIPEA (467 µL, 4 Eq, 2.68 mmol) forming a bright yellow solution. This solution was stirred for 15 minutes to ensure complete activation. Separately, a solution of **32** (250 mg, 1 Eq, 670 µmol) was prepared in DMF (1 mL) and then added dropwise to the activated peptide. This solution was stirred at 23 °C for 30 minutes, then the reaction was concentrated under vacuum and precipitated into water (45 mL) to afford the product (459 mg, 544 µmol, 81% yield) as a white solid. This was carried forward without any further purification.

**HRMS (ESI/Q-TOF):** $[M+H]^+$ calculated for $C_{45}H_{59}N_6O_{10}^+$ 843.4287, observed 843.4309.

**Synthesis of Ac-Ala-Lys[Tyr($^t$Bu)-Ala-(Fmoc)Ala]-2-methylethylamino trifluoroacetate (34)**

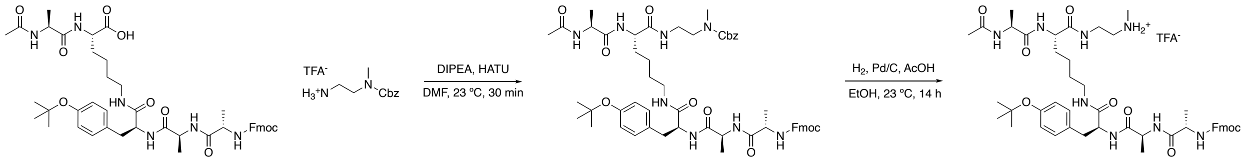

To a solution of **33** (456 mg, 1 Eq, 540 µmol) in DMF (7 mL) was added HATU (226 mg, 1.1 Eq, 595 µmol) and DIPEA (471 µL, 5 Eq, 2.70 mmol) forming a bright yellow solution. This solution was stirred for 15 minutes to ensure complete activation. Separately, a solution of **16** (158 mg, 1.2 Eq, 649 µmol) was prepared in DMF (2 mL) and then added dropwise to the activated peptide. This solution was stirred at 23 ºC for 30 minutes, then the reaction was concentrated under vacuum and precipitated into water (45 mL) to afford the intermediate as a white solid. This was carried forward without any further purification.

**HRMS (ESI/Q-TOF):** $[M+H]^+$ calculated for $C_{56}H_{73}N_8O_{11}^+$ 1033.5399, observed 1033.5392.

The resulting peptide was dissolved in ethanol (30 mL) with acetic acid additive* (618 µL, 20 Eq, 10.8 mmol) and combined with Pd/C (95 mg, 10 wt. % Pd, 0.1 Eq, 54 µmol). This solution was then sparged with argon, sealed under an atmosphere of hydrogen, and stirred at 23 °C for 14 hours. The reaction was filtered over Celite and concentrated under vacuum. The crude product was then purified *via* preparative HPLC (10-75% MeCN gradient against water, both with a 0.1% TFA additive) to afford the product (89 mg, 87.9 µmol, 16% yield) as a white solid.

*Acetic acid was used as an additive to protonate the secondary amine that is formed after the hydrogenolysis of the Cbz group. This formation of the ammonium salt prevents significant deprotection of the Fmoc group by the secondary amine.

**HRMS (ESI/Q-TOF):** $[M-TFA^-]^+$ calculated for $C_{48}H_{67}N_8O_9^+$ 899.5026, observed 899.5102.

**Synthesis of Ac-Ala-Lys[Tyr-Ala-Ala]-(2-(*p*NP-thionocarbamate)methyl)ethylamino trifluoroacetate (13)**

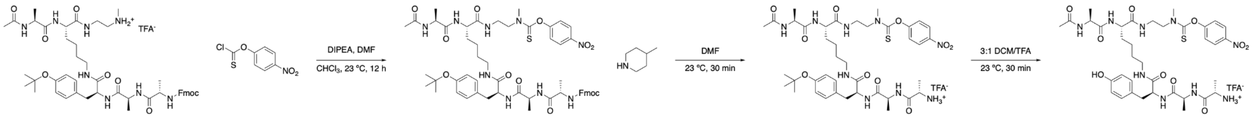

To a solution of **34** (25.0 mg, 1 Eq, 27.8 µmol) in $CHCl_3$ (2.5 mL) and DMF (0.5 mL) was added DIPEA (43.4 µL, 3 Eq, 83.5 µmol). This solution was stirred for five minutes before a solution of **4** (8.5 mg, 1.4 Eq, 39.0 µmol) in $CHCl_3$ (1.0 mL) was added. This solution was then vigorously stirred at 23 °C for 12 hours, then the reaction was concentrated under vacuum and precipitated into water (45 mL) to afford the product as a pale yellow solid. This was carried forward without any further purification.

The resulting peptide was dissolved in a 20% 4-methylpiperidine solution in DMF (v/v, 5 mL), which was stirred at 23 °C for 60 minutes and then concentrated under vacuum. The resulting crude product was then purified *via* preparative HPLC (10-100% MeCN gradient against water, both with a 0.1% TFA additive) to afford the O$^t$Bu protected peptide.

**HRMS (ESI/Q-TOF):** $[M\text{-}TFA^-]^+$ calculated for $C_{40}H_{60}N_9O_{10}S^+$ 858.4178, observed 858.4241.

The resulting peptide was dissolved in a 25% TFA solution in DCM (v/v, 10 mL), which was stirred at 23 °C for 30 minutes and then concentrated under vacuum. The resulting crude product was then precipitated into cold diethyl ether (45 mL, ca. -20 °C) to afford the product (17.1 mg, 18.7 µmol, 67% yield over three steps) as a white solid.

**HRMS (ESI/Q-TOF):** $[M\text{-}TFA^-]^+$ calculated for $C_{36}H_{52}N_9O_{10}S^+$ 802.3552, observed 802.3548.

**Synthesis of Ac-Lys[Tyr($^t$Bu)-Ala-Ala-(Fmoc)Ala] (15)**

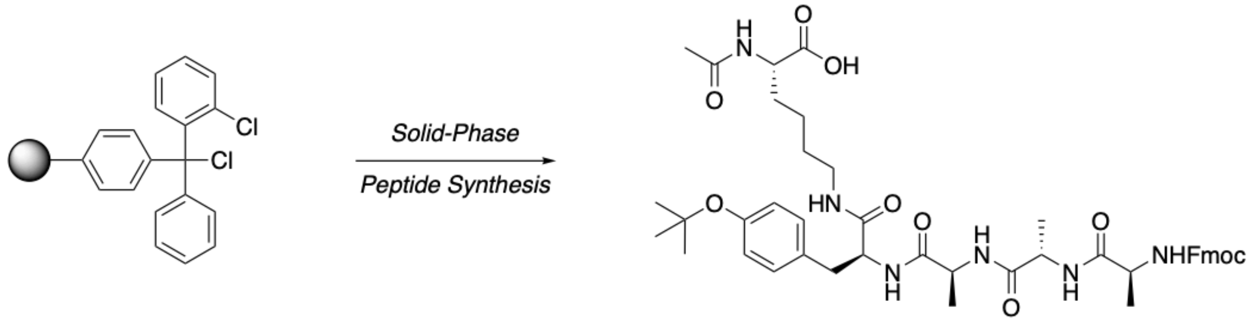


The peptide was synthesized *via* standard Fmoc Solid-Phase Peptide Synthesis conditions. The peptide was prepared using 1.000 g of 2-chlorotrityl chloride resin (0.89 mmol/g ChemImpex). The resin was swelled in DCM for 60 minutes prior to any modifications. The initial lysine coupling was carried out using two equivalents of Ac-Lys(Fmoc)-OH and six equivalents of DIPEA. This was done in 12 mL of 1:1 DCM and NMP (v/v) mixture and shaken for 90 minutes. Each amino acid residue thereafter was loaded using a 3:3:6 equivalent ratio of Fmoc-Amino Acid: HATU: DIPEA in NMP for 30 minutes (Equivalents relative to resin). The couplings were followed by deprotection in 20% 4-methylpiperidine in DMF (v/v, ca. 8 mL) for 20 minutes. After coupling the third alanine residue, the Fmoc protecting group was not cleaved. The resin was dried under vacuum and then swelled in DCM for 40 minutes. The peptide was cleaved from the resin while maintaining the Fmoc and *tert*-butyl protecting groups by using a 20% HFIP in DCM (v/v) cleavage cocktail (40 mL). Approximately 10 mL of the cleavage cocktail was added to the resin/peptide and mixed for two minutes. The resin was filtered and the flow through was collected, then the solvent was removed under vacuum. This process was repeated three additional times. After each removal of HFIP and DCM under vacuum, the concentrate was precipitated into chilled diethyl ether (45 mL, ca. -20 ºC) to afford the product (383 mg, 454 µmol, 51% yield) as a white solid.

**HRMS (ESI/Q-TOF):** $[M+H]^+$ calculated for $C_{45}H_{59}N_6O_{10}^+$ 843.4293, observed 843.4302.

**Synthesis of Ac-Lys[Tyr($^{t}$Bu)-Ala-Ala-(Fmoc)Ala]-2-methylethylamino trifluoroacetate (17)**

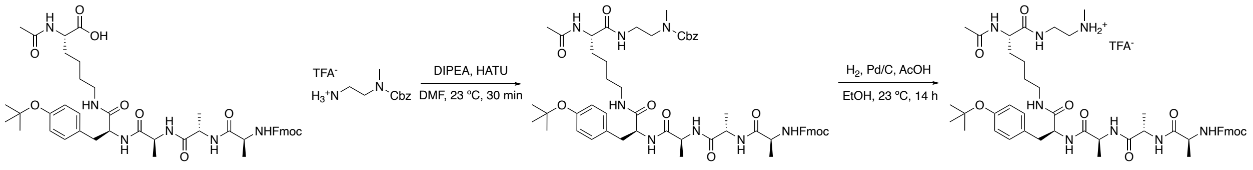


To a solution of **15** (100 mg, 1 Eq, 357 µmol) in DMF (3 mL) was added HATU (149 mg, 1.1 Eq, 393 µmol) and DIPEA (311 µL, 5 Eq, 1.79 mol). This solution was stirred for 15 minutes to ensure complete activation of the peptide. Separately, a solution of **16** (144 mg, 1.25 Eq, 445 µmol) was prepared in DMF (2 mL) and then added dropwise to the activated peptide. This solution was stirred at 23 ºC for 30 minutes, then the reaction was concentrated under vacuum and precipitated into water (45 mL) to afford the intermediate as a white solid. This was carried forward without any further purification.

**HRMS (ESI/Q-TOF):** $[M+H]^+$ calculated for $C_{56}H_{73}N_8O_{11}^+$ 1033.5399, observed 1033.5418.

The resulting peptide was dissolved in ethanol (15 mL) with acetic acid additive* (409 µL, 20 Eq, 7.14 mmol) and combined with Pd/C (25 mg, 10 wt. % Pd, 0.1 Eq, 36 µmol). This solution was then sparged with argon, sealed under an atmosphere of hydrogen, and stirred at 23 °C for 14 hours. The reaction was filtered over Celite and concentrated under vacuum. The crude product was then purified *via* preparative HPLC (30-95% MeCN gradient against water, both with a 0.1% TFA additive) to afford the product (41 mg, 40.4 µmol, 11% yield over two steps) as a white solid.

*Acetic acid was used as an additive to protonate the secondary amine that is formed after the hydrogenolysis of the Cbz group. This formation of the ammonium salt prevents significant deprotection of the Fmoc group by the secondary amine.

**HRMS (ESI/Q-TOF):** $[M\text{-}TFA^-]^+$ calculated for $C_{48}H_{67}N_8O_9^+$ 899.5026, observed 899.5123.

**Synthesis of Ac-Lys[Tyr($^t$Bu)-Ala-Ala-Ala]-(2-(*p*NP-thionocarbamate)methyl)ethylamino trifluoroacetate (14)**

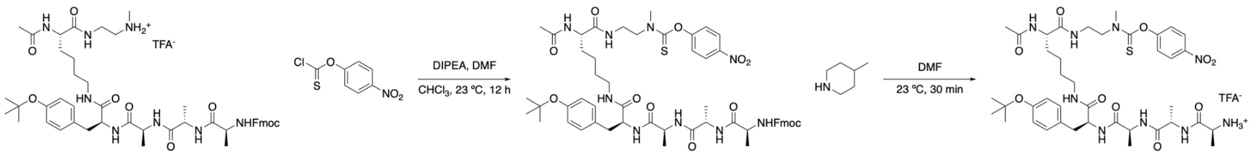


To a solution of **17** (29 mg, 1 Eq, 28.1 µmol) in $CHCl_3$ (2 mL) was added DIPEA (44 µL, 3 Eq, 84 µmol). This solution was stirred for five minutes before a solution of **4** (8.5 mg, 1.4 Eq, 39.0 µmol) in $CHCl_3$ (1.0 mL) was added. This solution was then vigorously stirred at 23 °C for 12 hours, then the reaction was concentrated under vacuum and precipitated into water (45 mL) to afford the product as a pale yellow solid. This was carried forward without any further purification.

The resulting peptide was dissolved in a 20% 4-methylpiperidine solution in DMF (v/v, 5 mL), which was stirred at 23 °C for 60 minutes and then concentrated under vacuum. The resulting crude product was then purified *via* preparative HPLC (10-100% MeCN gradient against water, both with a 0.1% TFA additive) to afford the product (13 mg, 13.4 µmol, 48% yield) as a white solid.

**HRMS (ESI/Q-TOF):** $[M\text{-}TFA^-]^+$ calculated for $C_{40}H_{60}N_9O_{10}S^+$ 858.4178, observed 858.4196.

# *p*NP Release Assays

### General Procedure – Release Kinetics

The peptide was dissolved in DMSO and diluted using 35 mM HEPES buffer (pH 7.5) to a final substrate concentration of 1.5 mM, maintaining a constant concentration of DMSO across all conditions. These solutions were then combined with either chymotrypsin, trypsin, or both enzymes to afford a final concentration of 0.02 mM for each enzyme. Immediately upon addition of the proteases, the 96-well plate was inserted into the plate reader and the absorbance at 405 nm was monitored over the course of 4 hours with time points taken every 30 seconds. The plate was incubated in the plate reader at 37 ºC.

### General Procedure – Michaelis-Menten Kinetics

The peptide was dissolved in DMSO and diluted using 35 mM HEPES buffer (pH 7.5) to a final substrate concentration of 2.0, 1.5, 1.0, 0.5, 0.24, and 0.1 mM, maintaining a constant concentration of DMSO across all conditions. These solutions were then combined with chymotrypsin and trypsin to afford a final concentration of 0.02 mM for each enzyme. Immediately upon addition of the proteases, the 96-well plate was inserted into the plate reader and the absorbance at 405 nm was monitored over the course of 4 hours with time points taken every 30 seconds. The plate was incubated in the plate reader at 37 ºC. All conditions were carried out in triplicate and reported as the average between the three replicates. Michaelis-Menten enzyme kinetic analysis was carried out using GraphPad Prism 8.4.3. Substrate concentrations were adjusted as needed to obtain relevant Michaelis-Menten statistics. Concentrations of substrate are shown in their respective activity plots below.

**Standard curve of *p*NP**

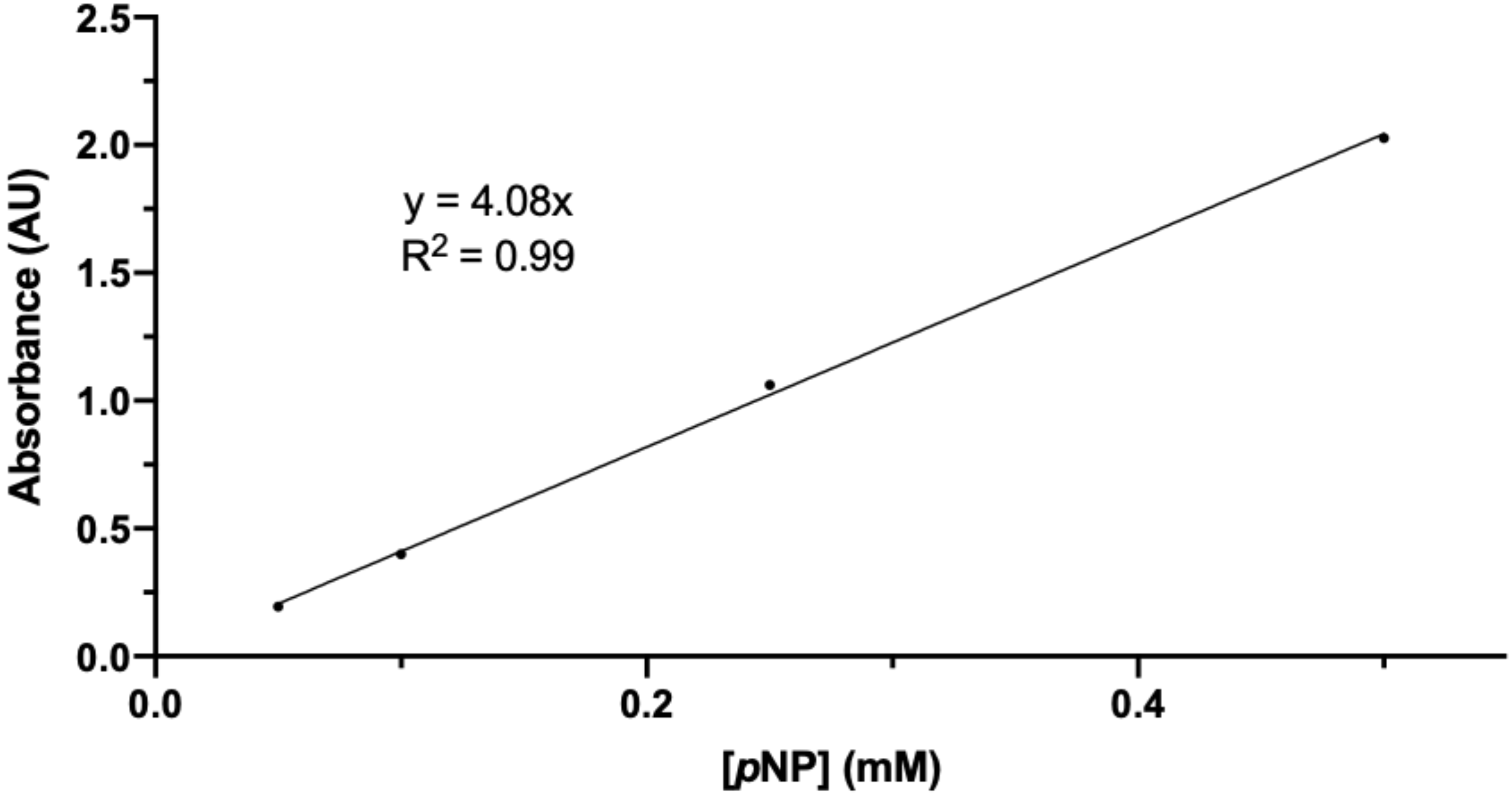


**Figure S12.** Standard curve of *p*NP at 405 nm used for release assays.

**Release of *p*NP from 6 (Lys[(Ac)Phe]-(2-(*p*NP-thionocarbamate)methyl)ethylamino trifluoroacetate) in the presence of only chymotrypsin**

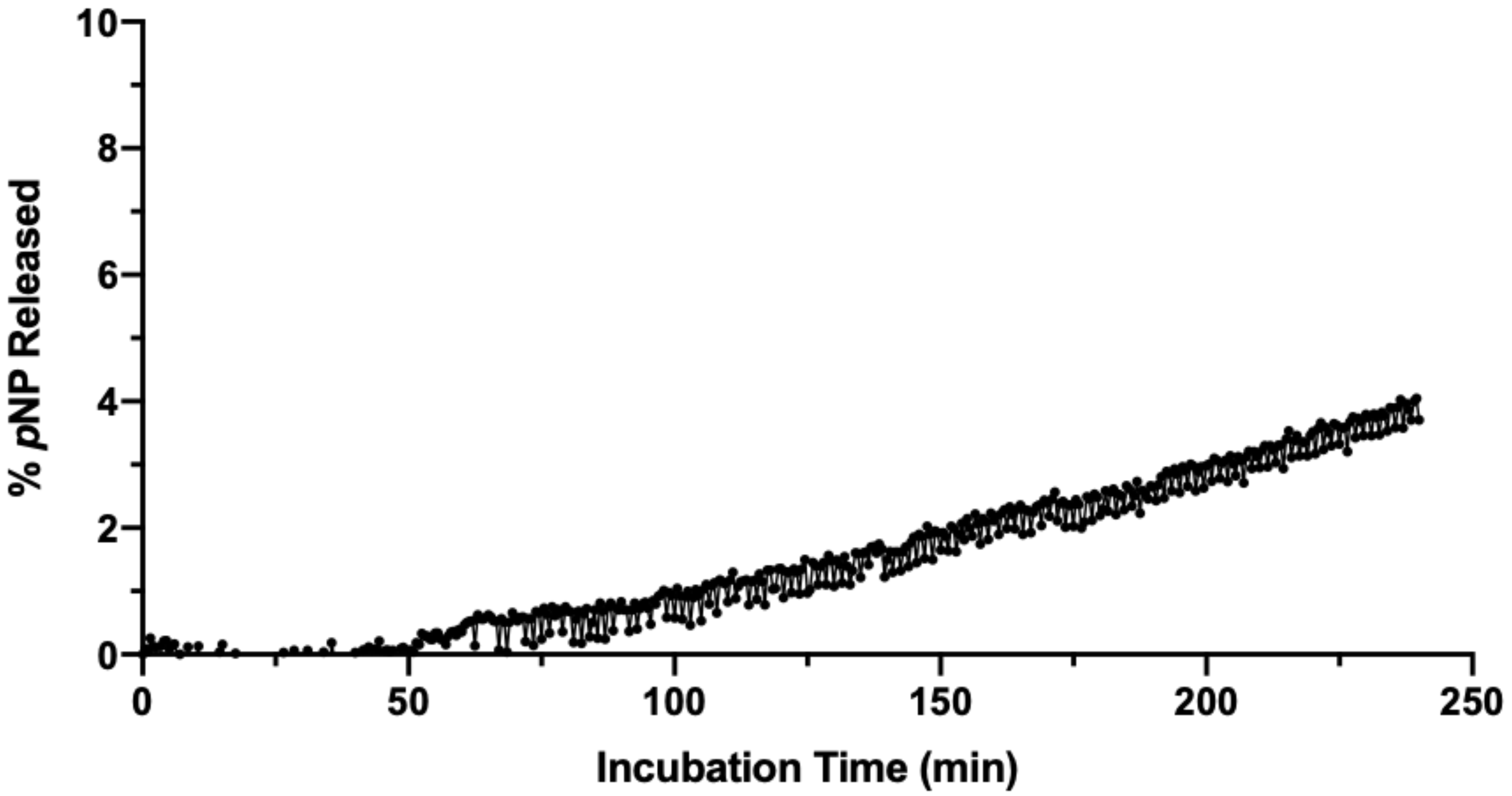


**Figure S13.** *p*NP release plot for **6** in the presence of chymotrypsin at a substrate concentration of 1.5 mM. This data is the same as shown in figure 2 of the manuscript, but it is enlarged for easier viewing.

**Release of *p*NP from 7 (Ala-Lys[(Ac)Phe]-(2-(*p*NP-thionocarbamate)methyl)ethylamino trifluoroacetate) in the presence of only chymotrypsin**

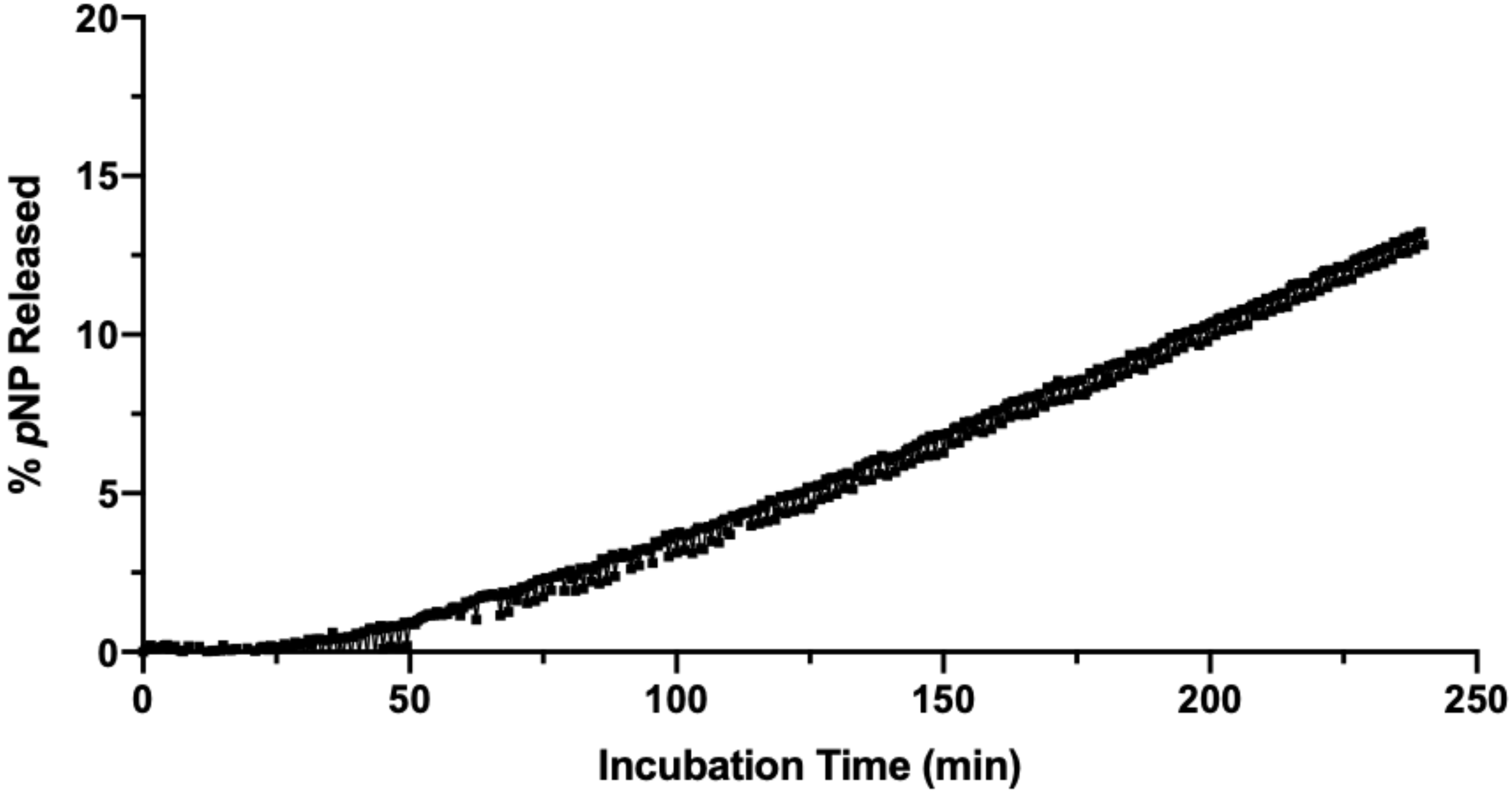


**Figure S14.** *p*NP release plot for **7** in the presence of chymotrypsin at a substrate concentration of 1.5 mM. This data is the same as shown in figure 2 of the manuscript, but it is enlarged for easier viewing.

**Release of *p*NP from 8 (Ala-Ala-Lys[(Ac)Phe]-(2-(*p*NP-thionocarbamate)methyl)ethylamino trifluoroacetate) in the presence of only chymotrypsin**

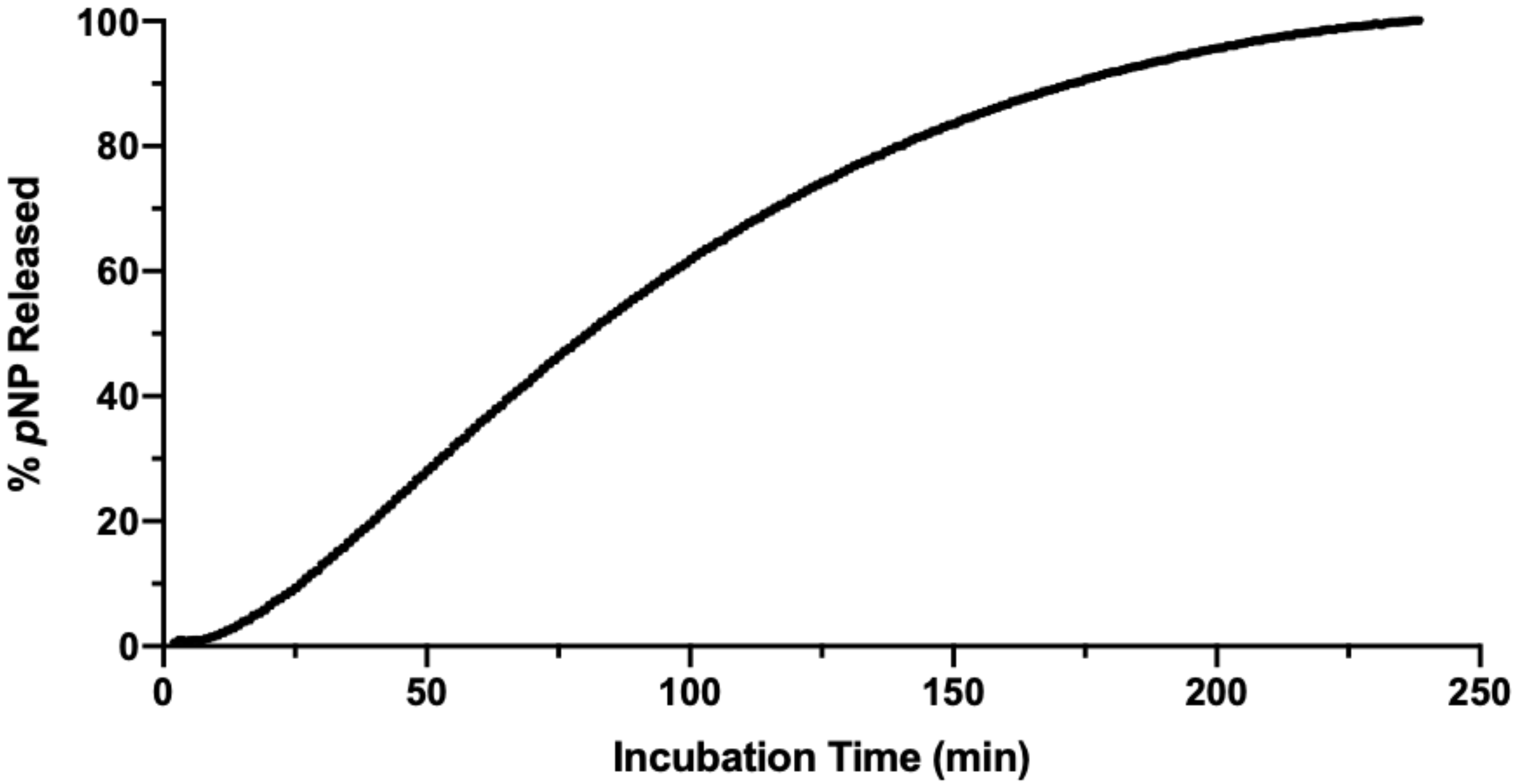


**Figure S15.** *p*NP release plot for **8** in the presence of chymotrypsin at a substrate concentration of 1.5 mM. This data is the same as shown in figure 2 of the manuscript, but it is enlarged for easier viewing.

**Michaelis-Menten plot of 7 (Ala-Lys[(Ac)Phe]-(2-(*p*NP-thionocarbamate)methyl)ethylamino trifluoroacetate) in the presence of chymotrypsin and trypsin**

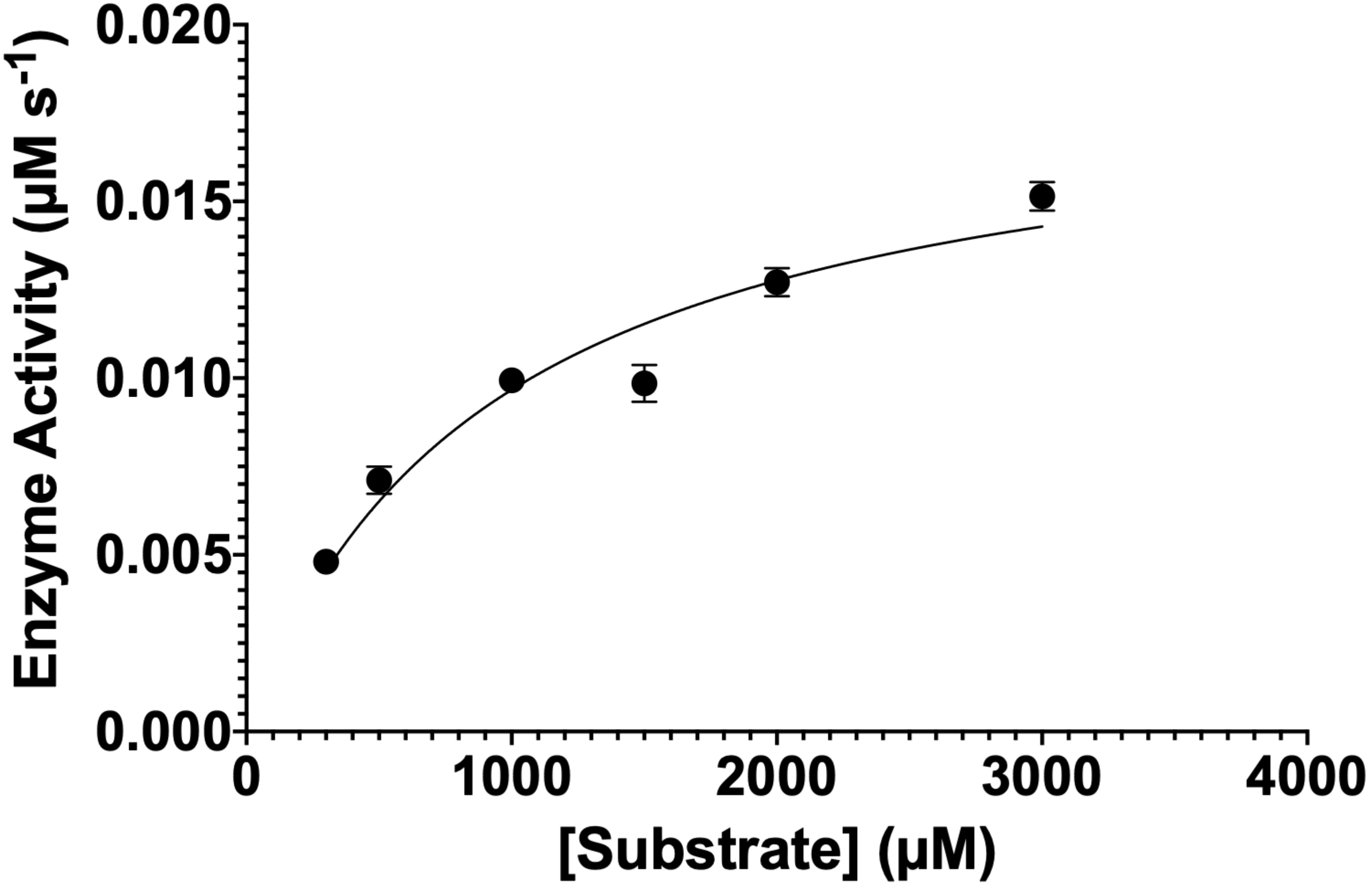


**Figure S16.** Michaelis-Menten plot for **7** in the presence of both chymotrypsin and trypsin. $k_{Cat}$ = 9.39x$10^{-4}$ $s^{-1}$, $K_M$ = 941 µM, $k_{Cat}/K_M$ = 9.97x$10^{-7}$ $\mu M^{-1}s^{-1}$, and $V_{Max}$ = 0.019 µM $s^{-1}$. n = 3, some error bars are smaller than the markers.

**Michaelis-Menten plot of 9 (Ac-Lys[Phe-Ala]-(2-(*p*NP-thionocarbamate)methyl)ethylamino trifluoroacetate) in the presence of chymotrypsin and trypsin**

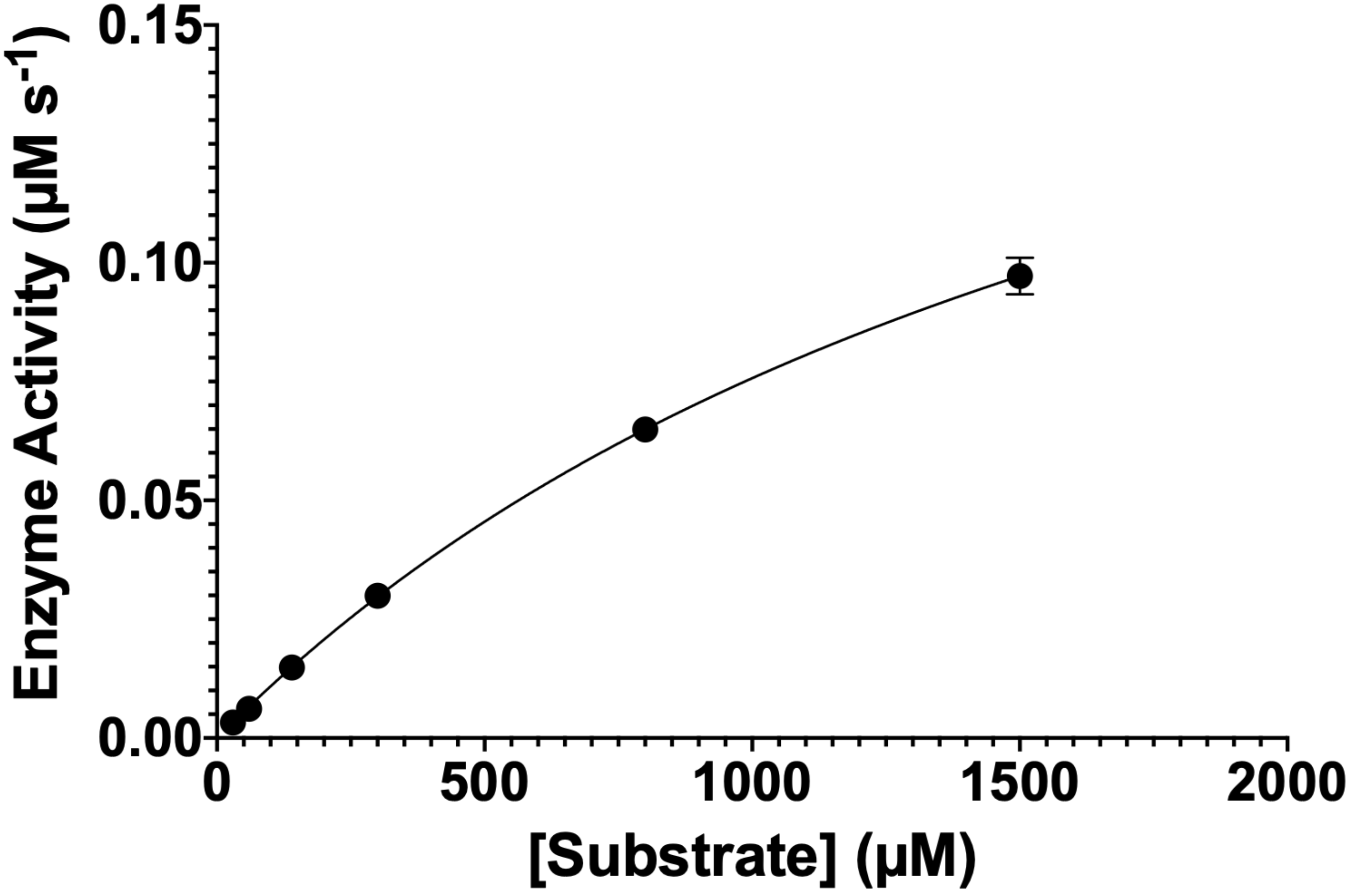


**Figure S17.** Michaelis-Menten plot for **9** in the presence of both chymotrypsin and trypsin. $k_{Cat}$ = 1.03x10$^{-3}$ s$^{-1}$, $K_M$ = 1423 µM, $k_{Cat}/K_M$ = 7.25x10$^{-7}$ µM$^{-1}$s$^{-1}$, and $V_{Max}$ = 0.021 µM s$^{-1}$. n = 3, some error bars are smaller than the markers.

**Michaelis-Menten plot of 10 (Ac-Lys[Phe-Ala-Ala]-(2-(*p*NP-thionocarbamate)methyl)ethylamino trifluoroacetate) in the presence of chymotrypsin and trypsin**

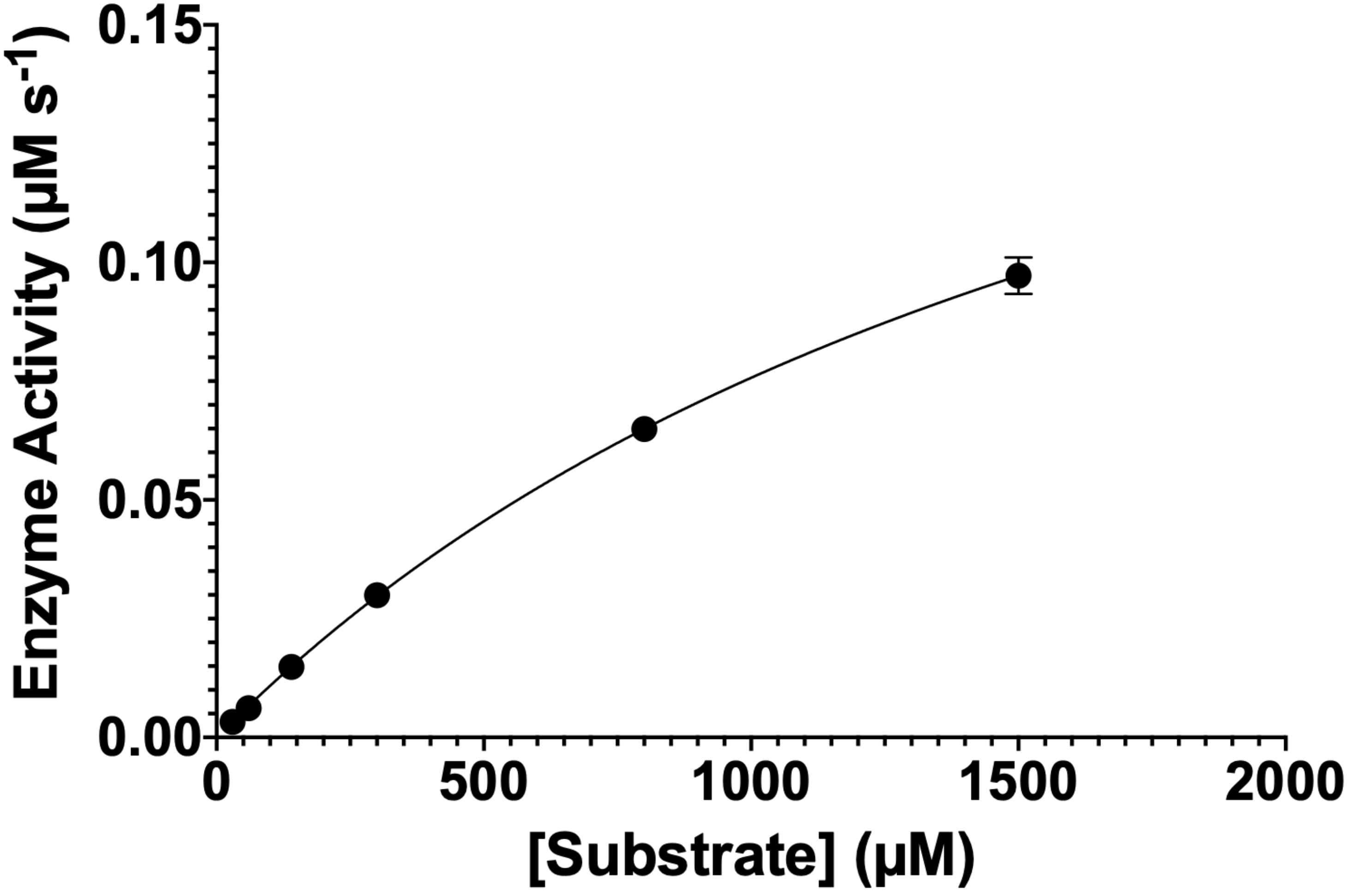


**Figure S18.** Michaelis-Menten plot for **10** in the presence of both chymotrypsin and trypsin. $k_{Cat} = 1.13x10^{-2}\ s^{-1}$, $K_M = 1973\ \mu M$, $k_{Cat}/K_M = 5.73x10^{-6}\ \mu M^{-1}s^{-1}$, and $V_{Max} = 0.225\ \mu M\ s^{-1}$. n = 3, some error bars are smaller than the markers.

**Michaelis-Menten plot of 11 (Ac-Lys[Tyr-Ala-Ala]-(2-(pNP-thionocarbamate)methyl)ethylamino trifluoroacetate) in the presence of chymotrypsin and trypsin**

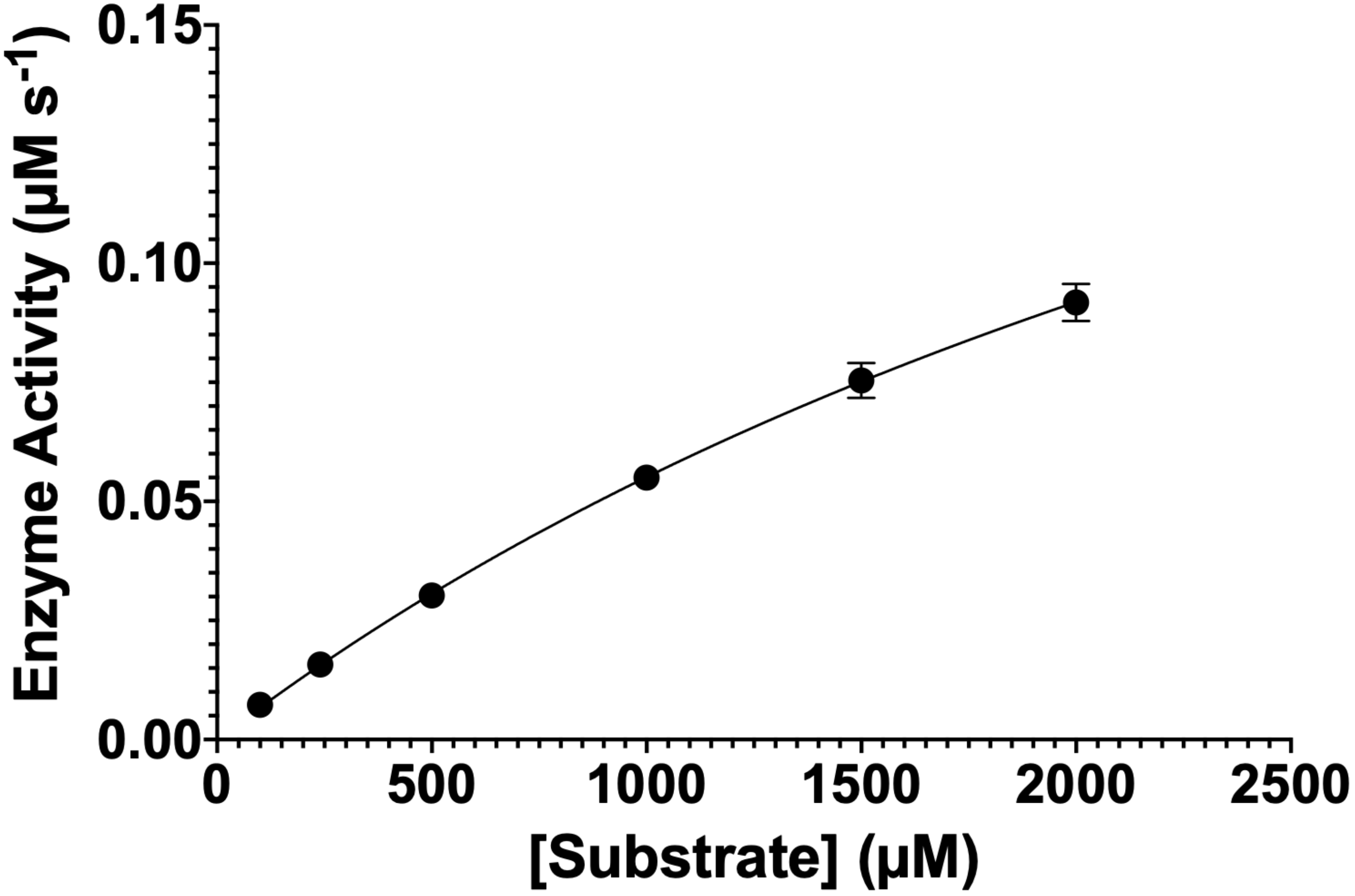


**Figure S19.** Michaelis-Menten plot for **11** in the presence of both chymotrypsin and trypsin. $k_{Cat}$ = 1.38x$10^{-2}$ $s^{-1}$, $K_M$ = 4003 µM, $k_{Cat}/K_M$ = 3.45x$10^{-6}$ µ$M^{-1}s^{-1}$, and $V_{Max}$ = 0.276 µM $s^{-1}$. n = 3, some error bars are smaller than the markers.

**Michaelis-Menten plot of 12 (Ac-Lys[Tyr-Ala-Ala-Ala]-(2-(*p*NP-thionocarbamate)methyl)ethylamino trifluoroacetate) in the presence of chymotrypsin and trypsin**

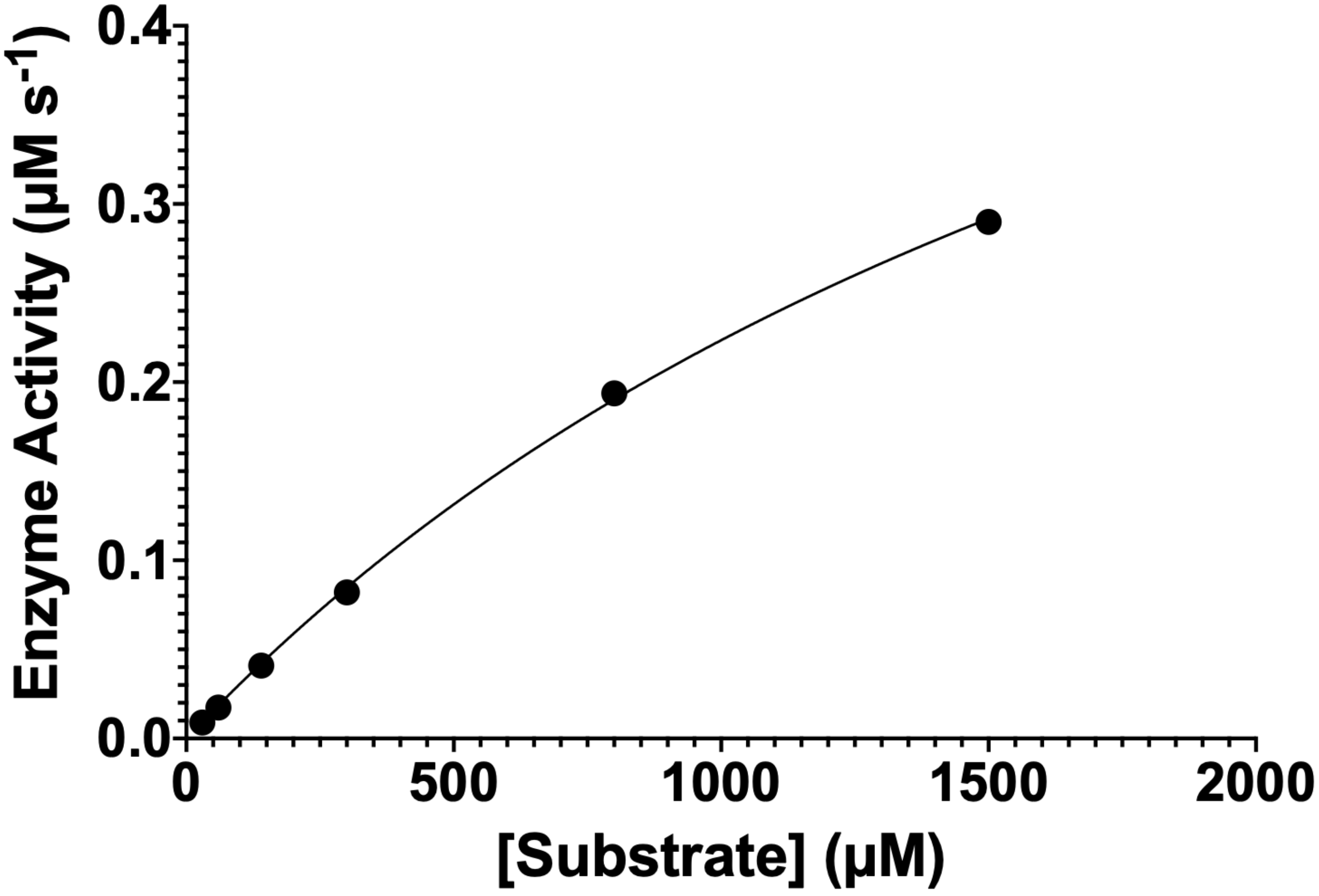


**Figure S20.** Michaelis-Menten plot for **12** in the presence of both chymotrypsin and trypsin. $k_{Cat}$ = 3.75x10$^{-2}$ s$^{-1}$, $K_M$ = 2351 µM, $k_{Cat}/K_M$ = 1.60x10$^{-5}$ µM$^{-1}$s$^{-1}$, and $V_{Max}$ = 0.749 µM s$^{-1}$. n = 3, some error bars are smaller than the markers.

**Michaelis-Menten plot of 13 (Ac-Ala-Lys[Tyr-Ala-Ala]-(2-(*p*NP-thionocarbamate)methyl)ethylamino trifluoroacetate) in the presence of chymotrypsin and trypsin**

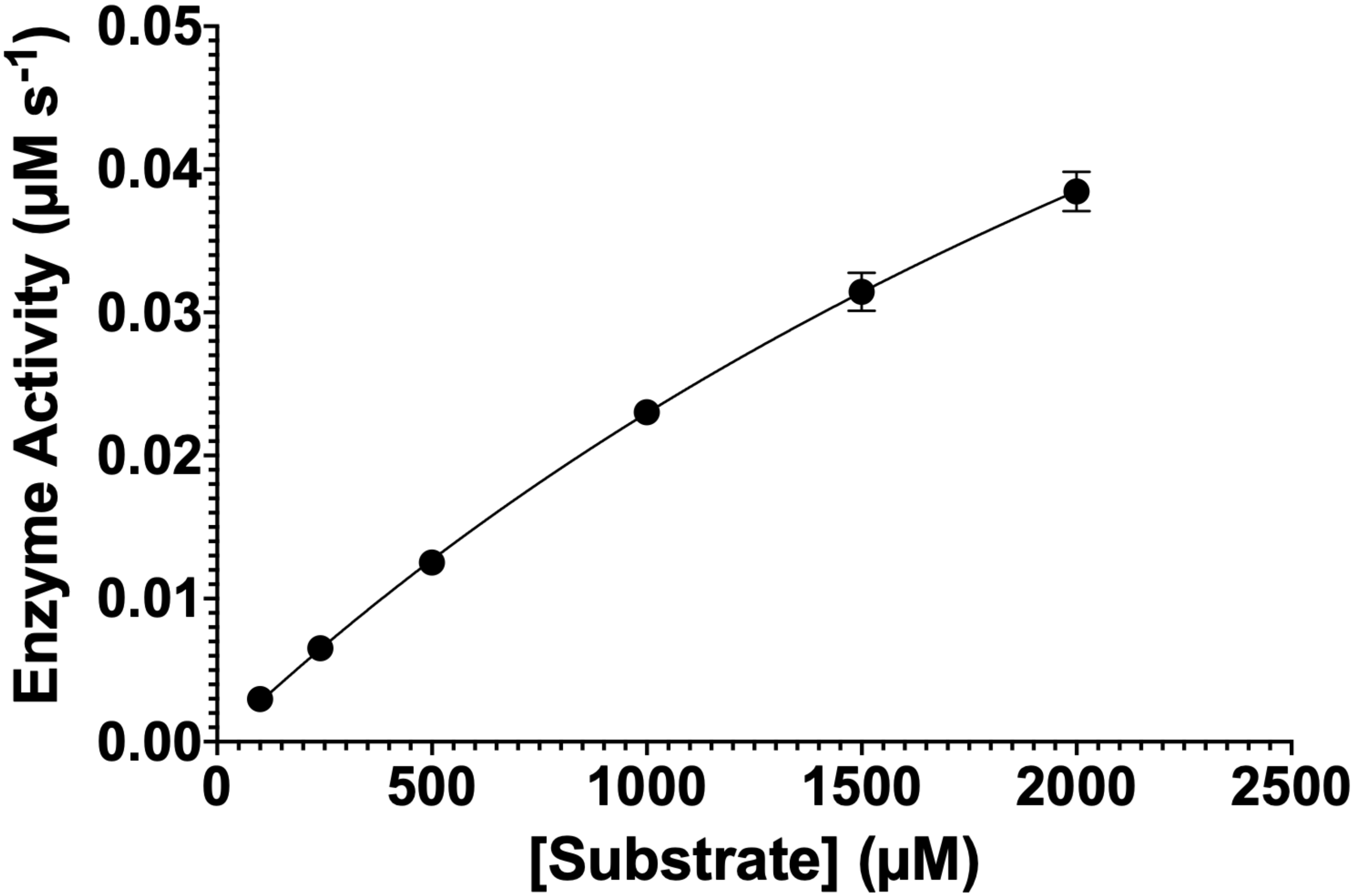


**Figure S21.** Michaelis-Menten plot for **13** in the presence of both chymotrypsin and trypsin. $k_{Cat} = 5.92x10^{-3}$ $s^{-1}$, $K_M = 4159$ µM, $k_{Cat}/K_M = 1.42x10^{-6}$ $\mu M^{-1}s^{-1}$, and $V_{Max} = 0.119$ µM $s^{-1}$. n = 3, some error bars are smaller than the markers.

**Release of *p*NP from 12 (Ac-Lys[Tyr-Ala-Ala-Ala]-(2-(*p*NP-thionocarbamate)methyl)ethylamino trifluoroacetate) in the presence of chymotrypsin and trypsin**

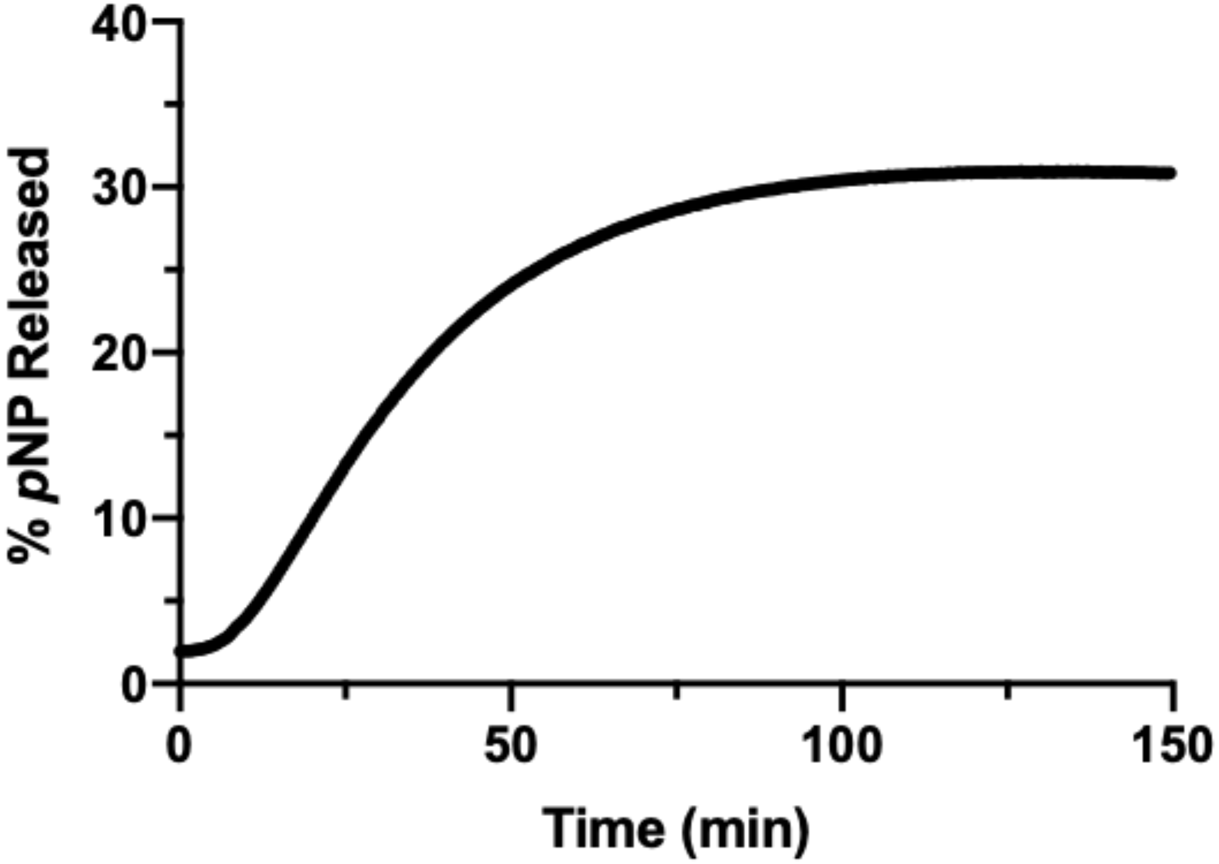


**Figure S22.** *p*NP release plot for **12** in the presence of both chymotrypsin and trypsin at a substrate concentration of 1.5 mM. n = 3, some error bars are smaller than the markers. This data is the same as shown in figure 3 of the manuscript, but it is enlarged for easier viewing.

**Release of *p*NP from 12 (Ac-Lys[Tyr-Ala-Ala-Ala]-(2-(*p*NP-thionocarbamate)methyl)ethylamino trifluoroacetate) in the presence of only trypsin**

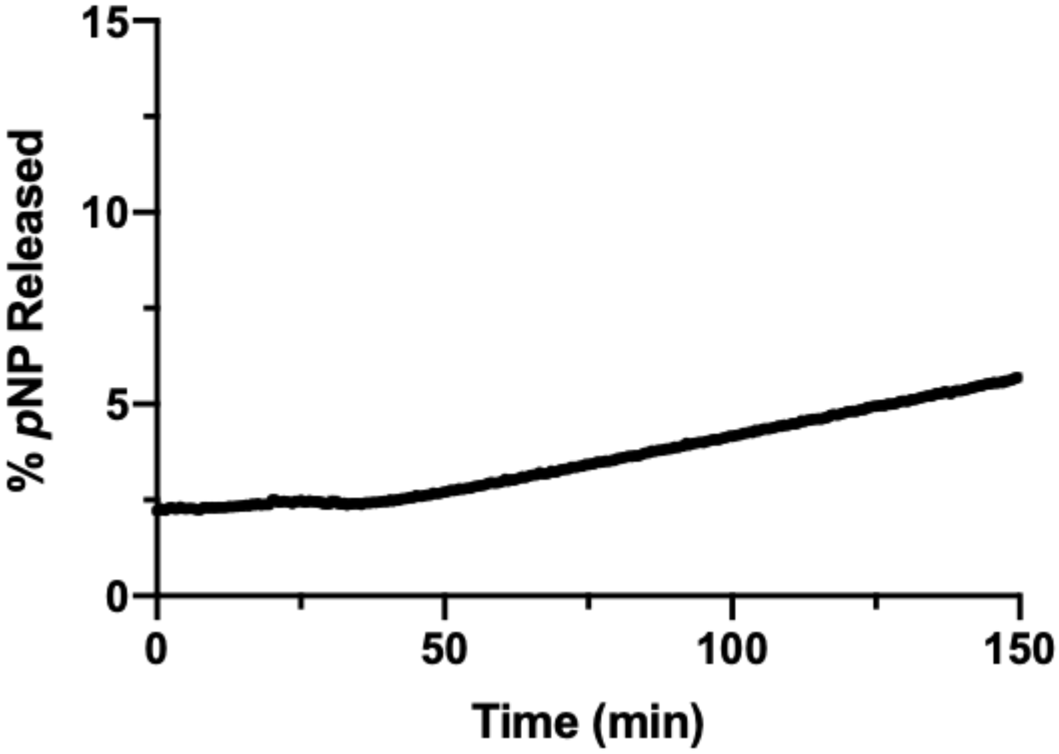


**Figure S23.** *p*NP release plot for **12** in the presence of only trypsin at a substrate concentration of 1.5 mM. n = 3, some error bars are smaller than the markers. This data is the same as shown in figure 3 of the manuscript, but it is enlarged for easier viewing.

**Release of *p*NP from 12 (Ac-Lys[Tyr-Ala-Ala-Ala]-(2-(*p*NP-thionocarbamate)methyl)ethylamino trifluoroacetate) in the presence of only chymotrypsin**

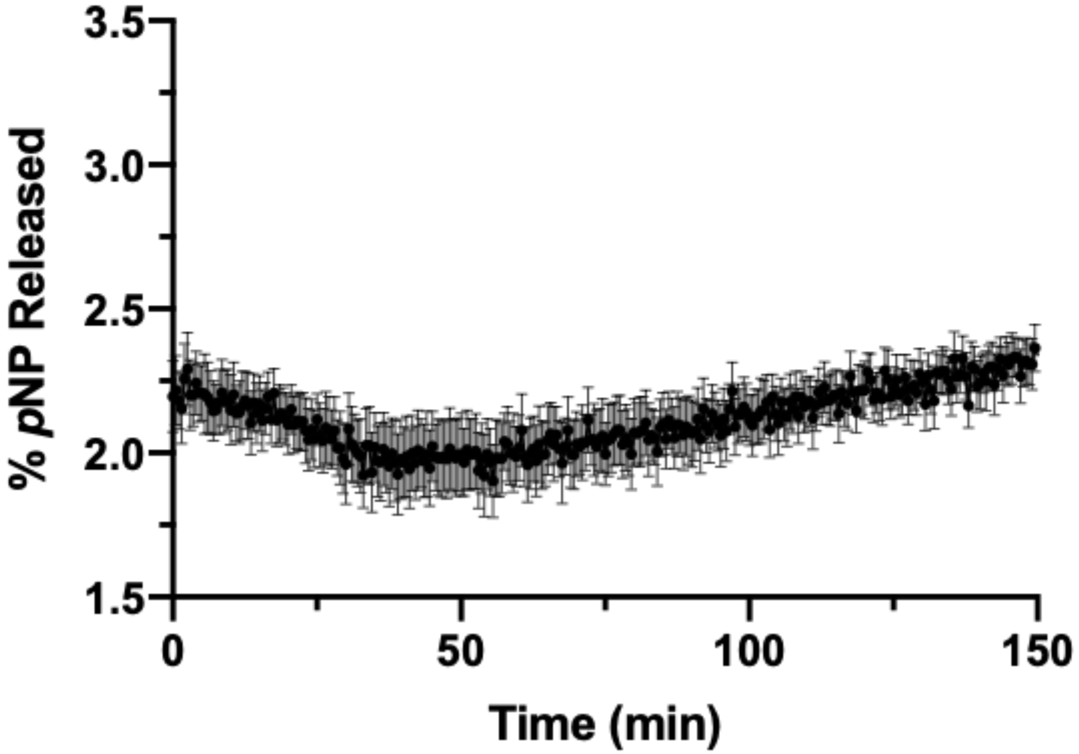


**Figure S24.** *p*NP release plot for **12** in the presence of only chymotrypsin at a substrate concentration of 1.5 mM. n = 3, some error bars are smaller than the markers. This data is the same as shown in figure 3 of the manuscript, but it is enlarged for easier viewing.

**Release of *p*NP from 14 (Ac-Lys[Tyr($^{t}$Bu)-Ala-Ala-Ala]-(2-(*p*NP-thionocarbamate)methyl)ethylamino trifluoroacetate) in the presence of chymotrypsin and trypsin**

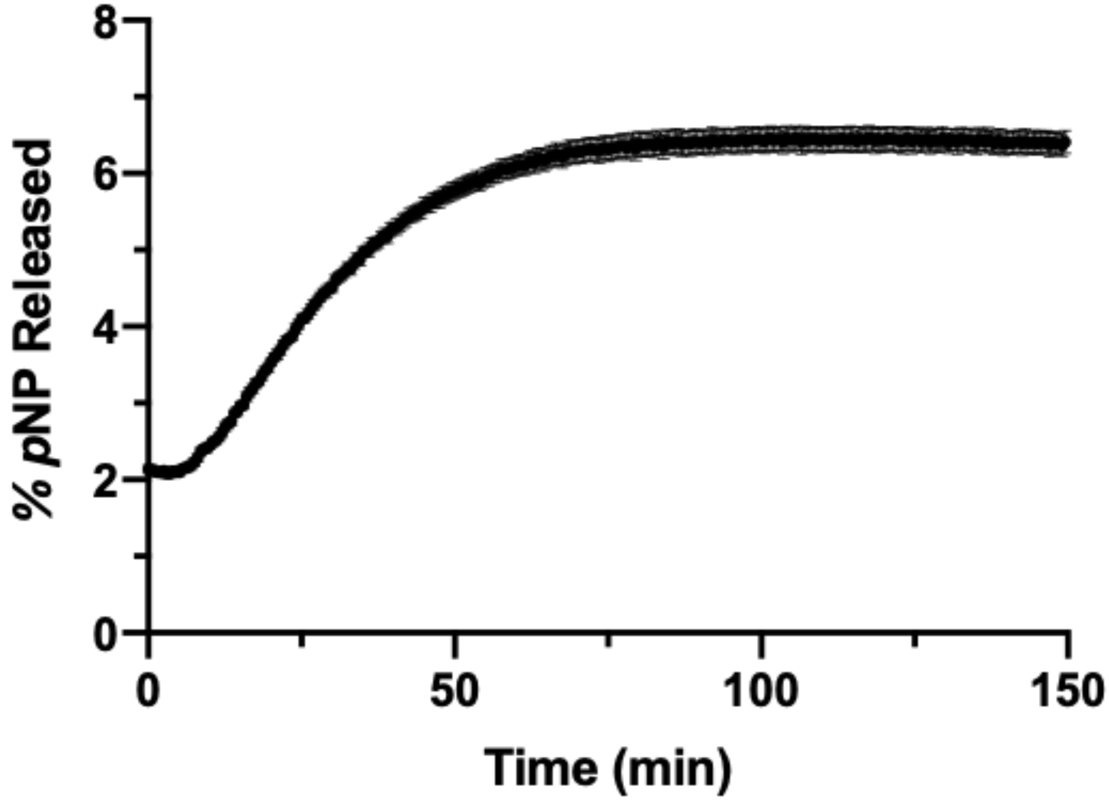


**Figure S25.** *p*NP release plot for **14** in the presence of both chymotrypsin and trypsin at a substrate concentration of 1.5 mM. n = 3, some error bars are smaller than the markers. This data is the same as shown in figure 3 of the manuscript, but it is enlarged for easier viewing.

**Release of *p*NP from 14 (Ac-Lys[Tyr(*t*Bu)-Ala-Ala-Ala]-(2-(*p*NP-thionocarbamate)methyl)ethylamino trifluoroacetate) in the presence of only trypsin**

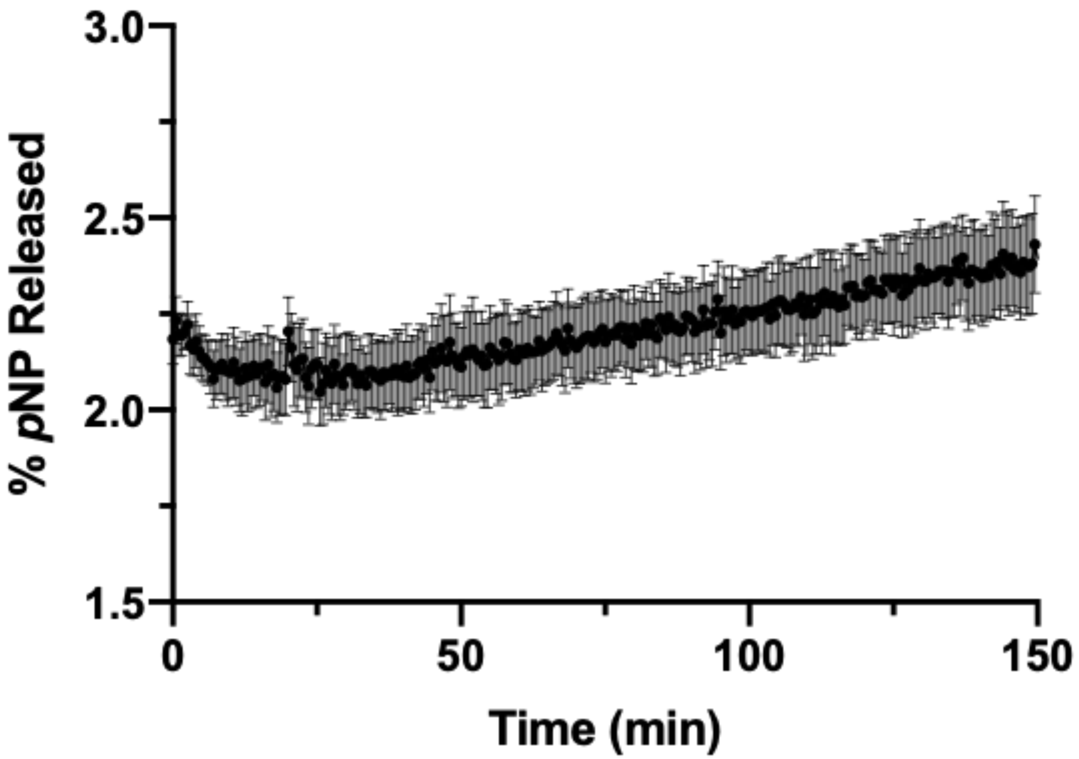


**Figure S26.** *p*NP release plot for **14** in the presence of only trypsin at a substrate concentration of 1.5 mM. n = 3, some error bars are smaller than the markers. This data is the same as shown in figure 3 of the manuscript, but it is enlarged for easier viewing.

**Release of *p*NP from 14 (Ac-Lys[Tyr($^t$Bu)-Ala-Ala-Ala]-(2-(*p*NP-thionocarbamate)methyl)ethylamino trifluoroacetate) in the presence of only chymotrypsin**

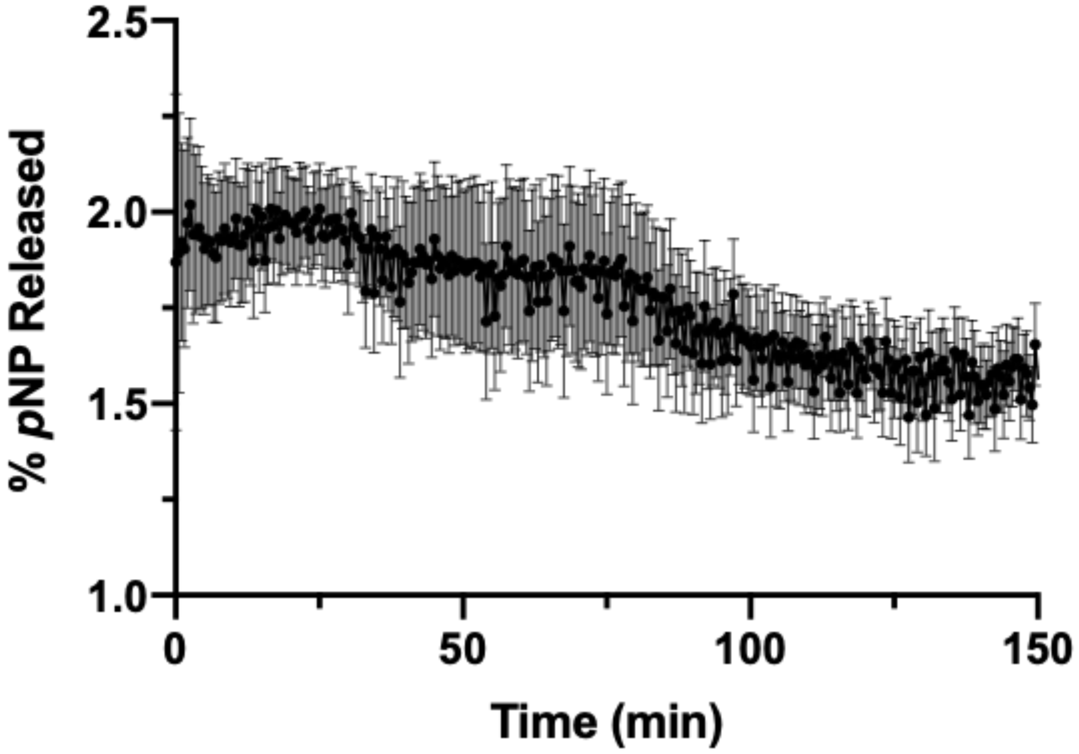


**Figure S27.** *p*NP release plot for **14** in the presence of only chymotrypsin at a substrate concentration of 1.5 mM. n = 3, some error bars are smaller than the markers. This data is the same as shown in figure 3 of the manuscript, but it is enlarged for easier viewing.

## Synthesis of Oxycodone-Containing Prodrugs

**Synthesis of Ac-Lys[Tyr($^t$Bu)-Ala-Ala-Ala]-(2-(oxycodone-thionocarbamate)methyl)ethylamino ditrifluoroacetate (18)**

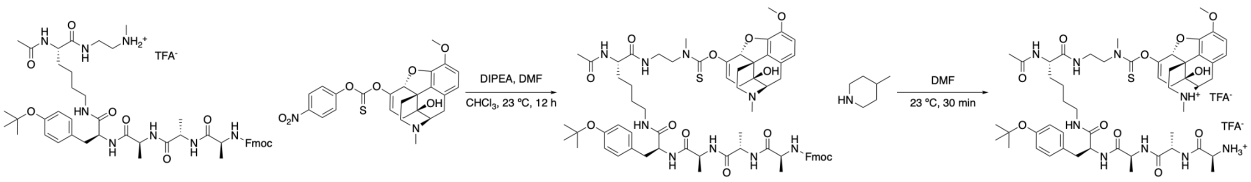


To a solution of **17** (34 mg, 1 Eq, 33.6 µmol) in $CHCl_3$ (2 mL) was added DIPEA (59 µL, 10 Eq, 336 µmol). This solution was stirred for five minutes before a solution of **5** (25.0 mg, 1.5 Eq, 50.3 µmol) in $CHCl_3$ (1.0 mL) was added. This solution was then vigorously stirred at 23 °C for 12 hours, then the reaction was concentrated under vacuum and precipitated into diethyl ether (45 mL) to afford the product as a pale yellow solid. This was carried forward without any further purification.

**HRMS (ESI/Q-TOF):** $[M+H]^+$ calculated for $C_{67}H_{86}N_9O_{13}S^+$ 1256.6060, observed 1256.6073.

The resulting peptide was dissolved in a 20% 4-methylpiperidine solution in DMF (v/v, 5 mL), which was stirred at 23 °C for 30 minutes and then concentrated under vacuum. The resulting crude product was triturated two times in 20 mL of diethyl ether, then the material was further purified *via* preparative HPLC (10-100% MeCN gradient against water, both with a 0.1% TFA additive) to afford the product (14.4 mg, 11.4 µmol, 34% yield) as a white solid.

**HRMS (ESI/Q-TOF):** $[M-2(TFA^-)-(H^+)]^+$ calculated for $C_{52}H_{76}N_9O_{11}S^+$ 1034.5380, observed 1034.5402.

**Synthesis of Ac-Lys[Tyr-Ala-Ala-Ala]-(2-(oxycodone-thionocarbamate)methyl)ethylamino ditrifluoroacetate (20)**

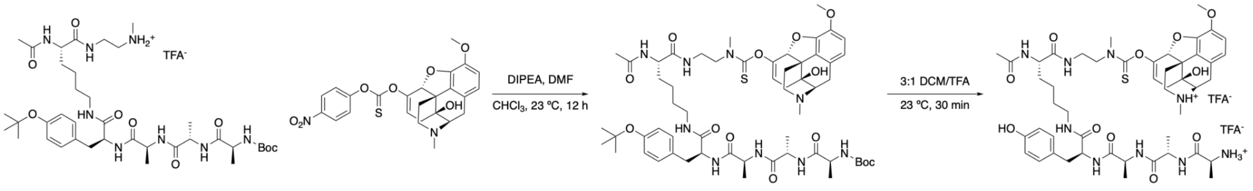


To a solution of **30** (76.0 mg, 1 Eq, 85.3 μmol) in $CHCl_3$ (2.5 mL) and DMF (0.5 mL) was added DIPEA (0.15 mL, 10 Eq, 853 μmol). This solution was stirred for five minutes before a solution of **5** (52.9 mg, 1.25 Eq, 107 μmol) in $CHCl_3$ (1.0 mL) was added. This solution was then vigorously stirred at 23 °C for 12 hours, then the reaction was concentrated under vacuum and precipitated into water (45 mL) to afford the intermediate as an off-white solid. This was carried forward without any further purification.

**HRMS (ESI/Q-TOF):** $[M+H]^+$ calculated for $C_{57}H_{84}N_9O_{13}S^+$ 1134.5909, observed 1134.5998.

The resulting peptide was dissolved in a 25% TFA solution in DCM (v/v, 10 mL). This was stirred at 23 °C for 30 minutes and then concentrated under vacuum. The resulting crude product was then purified *via* preparative HPLC (10-50% MeCN gradient against water, both with a 0.1% TFA additive) to afford the product (58.1 mg, 48.1 μmol, 56% yield) as a white solid.

**HRMS (ESI/Q-TOF):** $[M-2(TFA^-)-(H^+)]^+$ calculated for $C_{48}H_{68}N_9O_{11}S^+$ 978.4754, observed 978.4779.

# Misuse Deterrence of Prodrugs Against Common Chemicals

## General Procedure

A 2 mM sample of **18** was prepared in deionized water, then 25 µL of this solution was added to an Eppendorf tube. 75 µL of Coca-Cola®, vinegar, saturated sodium bicarbonate (Baking soda), or a pre-dissolved solution of a digestive enzyme kit was added to the Eppendorf tube.* This was left at 23 ºC for 24 hours. After 24 hours, an HPLC of the solution was taken to determine the amount of oxycodone release. Representative HPLC traces are shown at 254 nm, and these results were performed with n = 2.

*To prepare the digestive enzyme solution, one Enzymedica Digest Basic® caplet[1] was crushed with a mortar and pestle. The contents of the caplet were suspended in 5 mL of deionized water to generate the solution.

Alternatively, 2 mM sample of **18** was prepared in deionized water, then 25 µL of this solution was added to an Eppendorf tube. 75 µL of 100 mM citrate-phosphate buffer (Either pH 2.0, 4.0, 8.0, or 10.0) was added to the Eppendorf tube. This was left at 23 ºC for 24 hours. After 24 hours, an HPLC of the solution was taken to determine the amount of oxycodone release. Representative HPLC traces are shown at 254 nm, and these results were performed with n = 2.

### Household Chemical Manipulation Assay

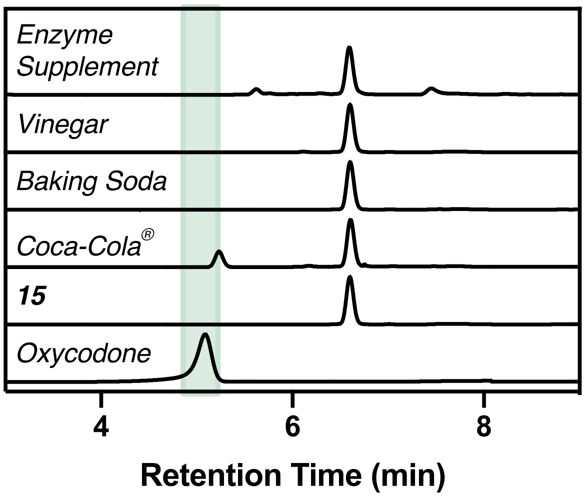


**Figure S28.** HPLC traces of **18** after subjecting to different household chemicals that can be used to degrade misuse deterrent prodrug formulations. Peak at 5.3 minutes in the Coca-Cola® run corresponds to aspartame. This data is the same as shown in figure 4 of the manuscript, but it is enlarged for easier viewing.

### pH Misuse Deterrence Assay

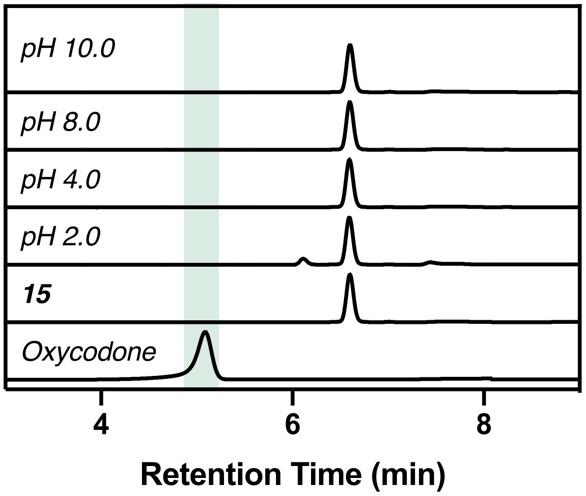


**Figure S29.** HPLC traces of **18** after subjecting to different pH buffers that can be used to degrade misuse deterrent prodrug formulations. HPLC traces are shown at 254 nm. This data is the same as shown in figure 4 of the manuscript, but it is enlarged for easier viewing.

## Synthesis of Tyrosine Containing Peptide Byproducts

### Synthesis of Ala-Ala-Ala-Tyr trifluoroacetate (19)

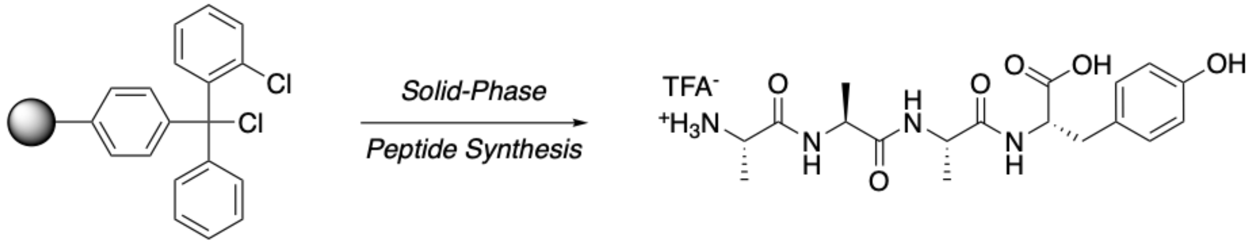


The peptide was synthesized via standard Fmoc Solid-Phase Peptide Synthesis conditions. The peptide was prepared using 1.000 g of 2-Chlorotrityl chloride resin (0.73 mmol/g ChemImpex). The resin was swelled in DCM for 60 minutes prior to any modifications. The initial tyrosine coupling was carried out using two equivalents of Fmoc-Tyrosine($^t$Bu)-OH and 6 equivalents of DIPEA. This was done in 12 mL of 1:1 DCM and NMP (v/v) mixture and shaken for 90 minutes. Each amino acid residue thereafter was loaded using a 3:3:6 equivalent ratio of Fmoc- or Boc-Amino Acid: HATU: DIPEA in NMP for 30 minutes. The couplings were followed by deprotection in 20% 4-methylpiperidine in DMF (v/v, ca. 8 mL) for 20 minutes. The third alanine residue was coupled as a Boc-alanine and left intact following the coupling. The resin was dried under vacuum for 120 minutes and then swelled in DCM for 40 minutes. The peptide was cleaved from the resin while simultaneously removing the Boc and *tert*-butyl protecting groups by using a 20% TFA in DCM (v/v) cleavage cocktail. Approximately 10 mL of the cleavage cocktail was added to the resin/peptide and mixed for two minutes. The resin was filtered and the flow through was collected, then the solvent was removed under vacuum. This process was repeated three additional times. After each removal of TFA and DCM under vacuum, the concentrate was precipitated into chilled diethyl ether (45 mL, ca. -20 °C) to afford the product (286 mg, 562 μmol, 77% yield) as a white solid.

**HRMS (ESI/Q-TOF):** $[M\text{-}TFA^-]^+$ calculated for $C_{18}H_{27}N_4O_6^+$ 395.1925, observed 395.2605.

## Competitive Inhibition Release Assay

### General Procedure

**12** was dissolved in DMSO and diluted using 35 mM HEPES buffer (pH 7.5) to a final substrate concentration of 2.0, 1.5, 1.0, 0.5, 0.24, and 0.1 mM, maintaining a constant concentration of DMSO across all conditions. **19** was added to yield a final concentration of either 1.6, 0.8, 0.4, or 0.2 mM. These solutions were then combined with trypsin and chymotrypsin to afford a final concentration of 0.02 mM for each enzyme. Immediately upon addition of the proteases, the 96-well plate was inserted into the plate reader and the absorbance at 405 nm was monitored over the course of 4 hours. All conditions were carried out in triplicate and reported as the average between the three replicates. Michaelis-Menten enzyme kinetic analysis was carried out using GraphPad Prism 8.4.3.

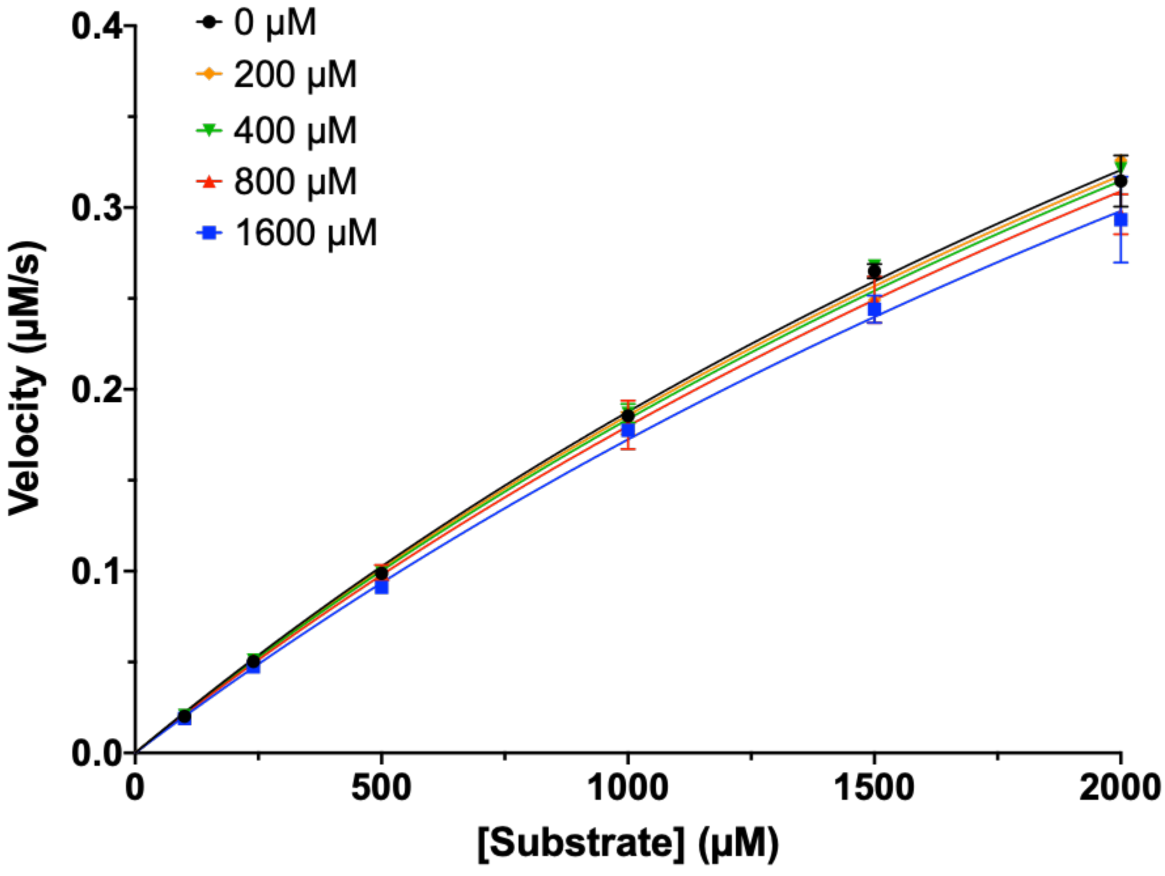


**Figure S30.** Michaelis-Menten competitive inhibition plot for **12** in the presence of chymotrypsin, trypsin, and the peptide-based inhibitor (**19**). n = 3, some error bars are smaller than the markers.

## *In Vivo* Studies of Analgesic Effects

### General Experimental Details

Male and female C57BL/6J mice were obtained (Jackson Laboratories) at 8 weeks of age. Mice were housed in groups of four per cage on a 12 h reverse light/dark cycle with food and water available *ad libitum*. Mice were allowed to habituate to their housing environments for 1 week before handling. Experiments were conducted in the dark phase between 09:00 and 16:00 h. To the extent possible, experimenters handling mice were blind to sex and drug treatment. Importantly, tests were performed by experimenters' blind to experimental conditions. Mice were assigned to experimental conditions in a randomized block design so that factors such as time of day were counterbalanced over the experimental conditions. All procedures were preapproved by the University of California, Los Angeles Chancellor's Animal Research Council.

### Hot Plate Testing

Adult male and female C57Bl/6 mice (8–10 weeks of age) were habituated for 30 minutes to the testing environment, handling, and equipment for three consecutive days prior to beginning of experimentation. Mice handling involved tail marking, cupping, weighing, and scruffing. Baseline testing to a 55 ºC hot plate was performed one day prior to drug administration. Mice were randomly assigned to three cohorts: an experimental group with the prodrug **20** (30 mg/kg oxycodone), oxycodone (3 mg/kg), or a vehicle control (Water). All drugs and controls were administered *via* oral gavage at 0.1 mL/10 g body weight. Separate cohorts also received the prodrug **20** (30 mg/kg) by intraperitoneal administration to determine the potential for antinociceptive effects that may be independent of gastrointestinal required drug release. The hot plate test (55 ºC) was used to test the thermal withdrawal threshold of each treatment group. Latency to paw withdrawal was measured in all mice, where a cut off of three times the average baseline latency was used to prevent tissue damage in the event of no response. Thermal threshold testing was determined prior to and every ten or twenty minutes after treatment until thresholds returned to baseline values. Data were expressed as means +/- SEM for n = 4–7 per group and percent maximal possible effect (%MPE) was determined for the time course data using the formula: ([Threshold Latency – Baseline Latency]/Baseline Latency * 100).

### Statistical Analysis

Data are expressed as scatter plots for raw data with bar graphs to show the mean +/- SEM. Data was analyzed by GraphPad Prism (V9.2). All behavioral data were shown to fit assumptions of a general linear model and data were subjected to a one-way ANOVA with Dunnett's post hoc analysis.

## *In Vivo* Plotted Data

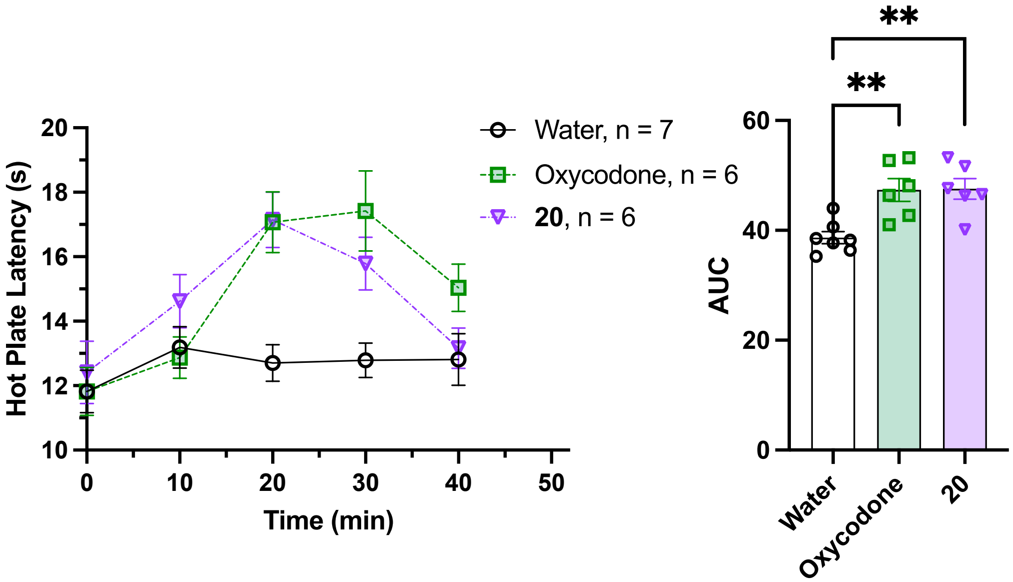


**Figure S31.** Hot plate latency data for PO gavage of **20** performed at 30 mg/kg oxycodone in prodrug and 3 mg/kg oxycodone along with the corresponding statistical analysis. ** = $p < 0.01$. This data is the same as shown in figure 5 of the manuscript, but it is enlarged for easier viewing.

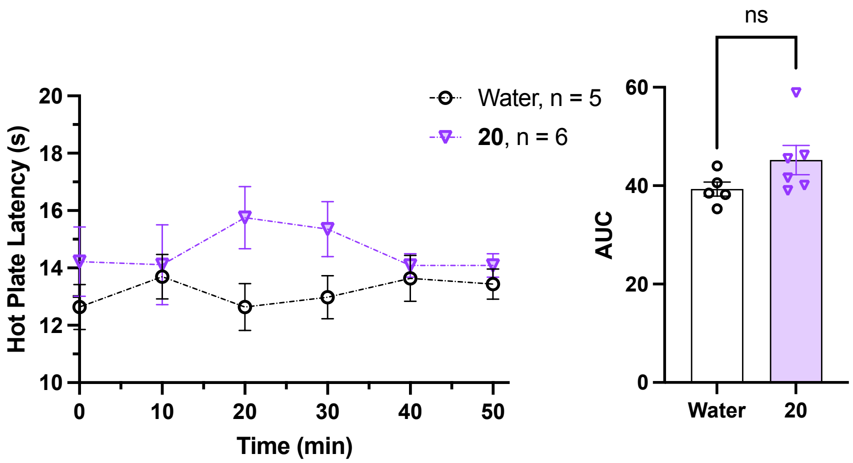


**Figure S32.** Hot plate latency data for IP injection of **20** performed at 30 mg/kg oxycodone in prodrug and water along with the corresponding statistical analysis. ns = not significant. This data is the same as shown in figure 5 of the manuscript, but it is enlarged for easier viewing.

## References

(1) Enzymedica Digest Basic. https://enzymedica.com/products/digest-basic-enzyme-digestion?variant=719909941#overview.
